# Comparative transcriptomic profiling of myxomatous mitral valve disease in the cavalier King Charles spaniel

**DOI:** 10.1186/s12917-020-02542-w

**Published:** 2020-09-23

**Authors:** G. R. Markby, V. E. Macrae, B. M. Corcoran, K. M. Summers

**Affiliations:** 1 The Roslin Institute, University of Edinburgh, Roslin Mid-Lothian, Roslin, Scotland, UK EH25 9RG; 2 Royal (Dick) School of Veterinary Studies, University of Edinburgh, Roslin Mid-Lothian, Roslin, Scotland, UK EH25 9RG; 3 Mater Research Institute-University of Queensland, 37 Kent St, Woolloongabba, QLD 4102 Australia

**Keywords:** Genes expression, Gene clustering, Gene networks, Myxomatous mitral valve disease, cavalier King Charles spaniel

## Abstract

**Background:**

Almost all elderly dogs develop myxomatous mitral valve disease by the end of their life, but the cavalier King Charles spaniel (CKCS) has a heightened susceptibility, frequently resulting in death at a young age and suggesting that there is a genetic component to the condition in this breed. Transcriptional profiling can reveal the impact of genetic variation through differences in gene expression levels. The aim of this study was to determine whether expression patterns were different in mitral valves showing myxomatous degeneration from CKCS dogs compared to valves from non-CKCS dogs.

**Results:**

Gene expression patterns in three groups of canine valves resulted in distinct separation of normal valves, diseased valves from CKCS and diseased valves from other breeds; the latter were more similar to the normal valves than were the valves from CKCS. Gene expression patterns in diseased valves from CKCS dogs were quite different from those in the valves from other dogs, both affected and normal. Patterns in all diseased valves (from CKCS and other breeds) were also somewhat different from normal non-diseased samples. Analysis of differentially expressed genes showed enrichment in GO terms relating to cardiac development and function and to calcium signalling canonical pathway in the genes down-regulated in the diseased valves from CKCS, compared to normal valves and to diseased valves from other breeds. F2 (prothrombin) (CKCS diseased valves compared to normal) and MEF2C pathway activation (CKCS diseased valves compared to non-CKCS diseased valves) had the strongest association with the gene changes. A large number of genes that were differentially expressed in the CKCS diseased valves compared with normal valves and diseased valves from other breeds were associated with cardiomyocytes including *CASQ2*, *TNNI3* and *RYR2*.

**Conclusion:**

Transcriptomic profiling identified gene expression changes in CKCS diseased valves that were not present in age and disease severity-matched non-CKCS valves. These genes are associated with cardiomyocytes, coagulation and extra-cellular matrix remodelling. Identification of genes that vary in the CKCS will allow exploration of genetic variation to understand the aetiology of the disease in this breed, and ultimately development of breeding strategies to eliminate this disease from the breed.

## Background

Myxomatous mitral valve disease (MMVD) is very common in elderly dogs of all genetic backgrounds [1, 2]. The cavalier King Charles spaniel (CKCS) breed has a particularly high prevalence of myxomatous mitral valve disease (MMVD), with earlier development and a more rapid progression to severe end-stage disease compared to most other dog breeds [3–6]. This breed association suggests that there is a genetic basis for MMVD in the CKCS. Interestingly, the CKCS breed exhibits unique physiological differences from other breeds such as defects in platelet function, macro-thrombocytopenia and elevated circulating 5-hydroxytryptamine (5HT, also known as serotonin) levels [7–11]. A rare familial macro-thrombocytopenia has been found to be associated with cardiac valvulopathies in children, and there is evidence to suggest dysregulation of serotonergic signalling might contribute to the aetiopathogenesis of MMVD in both the dog and human [12]. For example, rare instances of acquired valvulopathies have been reported in human patients with carcinoid syndrome (excessive circulating 5HT) and chronic usage of appetite suppressants and anti-Parkinsonian drugs that target 5HT receptors [13–15]. Furthermore, there is evidence of enhanced 5HT signalling in both human and canine MMVD valve tissue and valve interstitial cells (VIC) exposed to tensile strain, and genetic variation in the exons of the 5HT transporter gene (*SLC6A4*, also known as *SERT*) may be associated with MMVD development in the Maltese terrier [16, 17]. Changes in expression of the 5HT receptor gene are reported for the canine mitral valve transcriptome, and in the dog *HTR2B* expression is associated with disease progression [18–21]. However, 5HT does not induce disease phenotype in cultured VICs and expression of 5HT receptor genes is controlled by TGFβ1 [22]. Nevertheless, taken together, the evidence would suggest that the CKCS has a variant of MMVD that differs somewhat from other dogs, despite the end-stage valve pathology and outcome being the same [23].

For some time it has also been recognised that MMVD has a high degree of heritability in the CKCS and the success of breeding programmes increasing the age of onset support this [5]. One of the only two reported genome-wide association studies (GWAS) identified two loci with a total of 31 protein coding genes within these regions that could be affected, but follow up on these proteins has not been published [24, 25]. Attempts to find similar genetic mutations in CKCSs and Dachshunds to those reported for human familial MMVD have been unsuccessful [26]. While features of the condition show high heritability in certain breeds, it is unlikely that a single or small number of genetic variants are implicated.

There are limited tissue gene expression data for canine MMVD, but one study reported the transcriptomic profile of aged CKCS with advanced disease [18]. The analysis found that the most affected biologically relevant functions were inflammatory/immune response, cellular movement, cardiovascular development, extracellular matrix organization and epithelial-to-mesenchymal transition (EMT), not dissimilar to the one other canine report not involving CKCSs [18, 19]. Of particular note were changes in expression of a range of cathepsins, matrix metalloproteinases and ADAMTS (a disintegrin and metalloproteinase with thrombospondin motif) family members, important in extracellular matrix remodelling, and a range of genes involved in endothelial-to-mesenchymal transition (EndoMT) [18, 27]. More recently we have shown the canine MMVD valve transcriptome changes progressively as the disease worsens in a range of dog breeds, but also have identified by sample-to-sample network analysis that the cavalier King Charles spaniel (CKCS) valve transcriptome was distinct from that seen with non-CKCSs with similar disease severity (Markby et al., in press; doi: 10.3389/fgene.2020.00372).

While global transcriptomic profiling of all dogs can give insight into the possible signalling pathways involved in MMVD pathogenesis, it does not clarify any breed-specific changes. Considering the prevalence of MMVD in the CKCS, the physiological differences that might impact on MMVD pathogenesis and the apparent differential clustering of gene changes compared to non-CKCSs, the aim of this study was to compare the mitral valve transcriptome profile of the CKCS to non-CKCS with severe to very severe (Whitney grade 3–4) MMVD, and against the profile of normal non-CKCS young adult dogs.

## Results

### Characteristics of the dogs in the study

**Fig. 1:**
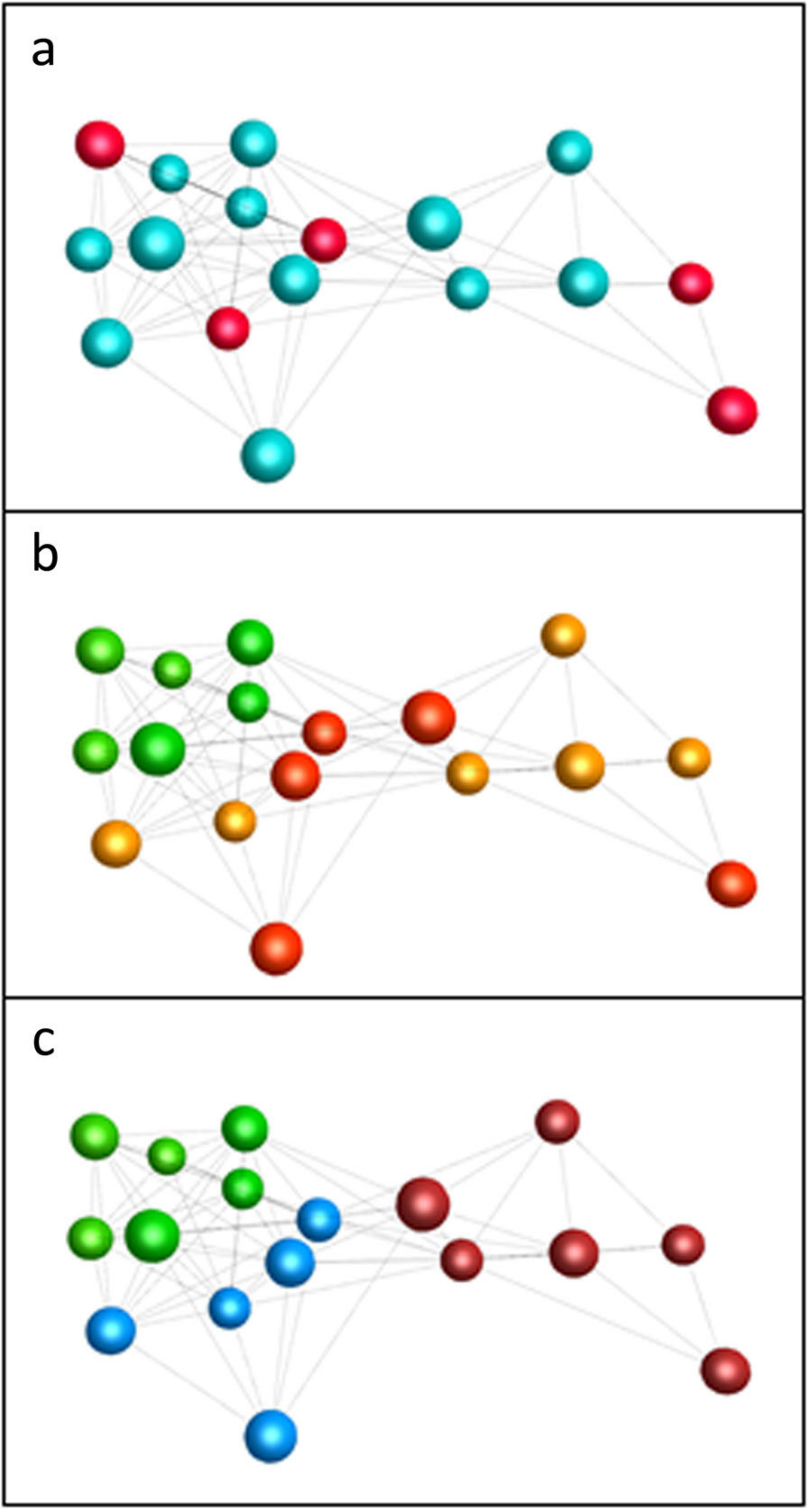
Sample-to-sample network analysis using BioLayout, showing relationships between gene expression patterns of mitral valve samples. Nodes (spheres) represent samples and edges (lines between samples) show a correlation between the expression profiles of samples of greater than 0.98. Similar samples are placed close together in the network. Each image shows the same network with nodes coloured based on different variables. **a**. Nodes are coloured by sex of the dog from which the valve was removed. Red – female; blue – male. **b**. Nodes are coloured by grade of MMVD disease found in the valve. Green − normal valves; Orange – Grade 3 diseased valves; red – Grade 4 diseased valves. **c**. Samples from CKCS diseased valves are separated from other samples. Green − normal valves; blue − non-CKCS diseased valves; dark red − CKCS diseased valves

Details of the animals used in the study are presented in Table 1. There was no significant difference in age between the two diseased groups, but both diseased groups were significantly older than the normal group (*P* < 0.001). Both sexes were represented in all groups and gender did not have an effect on gene expression, based on principal components analysis and network analysis (Fig. 1a). There were six valves with Grade 3 and 4 MMVD from CKCS and five Grade 3 and 4 valves were from other breeds (terrier breeds and Border Collies) (Fig. 1b and c). The normal dogs were Beagles and mixed breeds (Table 1).

**Table 1:** Metadata for valve samples analysed. Whitney gross pathology grade (0 normal to 4 very severe) was assigned independently by two of the authors (GRM and BMC).

Breed	Gender	Age	Whitney Grade
**Normal**			
Cross-terrier	Male	2 yrs	0
Cross-terrier	Male	3 yrs	0
Beagle	Male	3 yrs	0
Cross-Staffordshire bull terrier	Male	3 yrs	0
Beagle	Female	4 yrs	0
Beagle	Male	4 yrs	0
Mean Age +/−S.E.	3.1 yrs. +/−0.35		
**Non-CKCS**			
Cross-English bull terrier	Female	10 yrs	3
West Highland white terrier	Male	10 yrs	3
Jack Russel terrier	Female	11 yrs	4
Border collie	Male	13 yrs	4
Border collie	Male	13 yrs	4
Mean Age +/−S.E.	11.4 yrs. +/−0.27		
**CKCS**			
	Male	12 yrs	3
	Male	11 yrs	3
	Male	12 yrs	3
	Female	10 yrs	3
	Male	16 yrs	4
	Female	12 yrs	4
Mean Age +/−S.E.	12.5 yrs.+/−0.33		

There was no significant difference in age between the CKCS and non-CKCS group, but there was for both compared to normal group (*P* < 0.001)

### Network analysis of samples

To visualise the expression data, we used the network visualisation and analysis tool BioLayout (http://biolayout.org). Initially we wished to ascertain whether the samples could be grouped on the basis of disease status or breed. We therefore created a three dimensional sample-to-sample weighted network graph, based on a pairwise matrix of Pearson correlation coefficients. To minimise noise we filtered out low expression genes (normalised relative expression < 15). The graph was laid out at a Pearson correlation coefficient threshold of 0.98 (see Methods). This showed that expression patterns of all grade 3 and 4 samples were separated from the expression patterns of the normal samples (Fig. 1b), and there was also a clear separation of CKCS diseased valve samples away from the non-CKCS diseased samples, with the non-CKCS diseased valve samples closer to the normal valves in the network than were the CKCS diseased valves (Fig. 1c). This suggested that the diseased valves from non-CKCS and CKCS dogs had distinct patterns of gene expression.

**Fig. 2:**
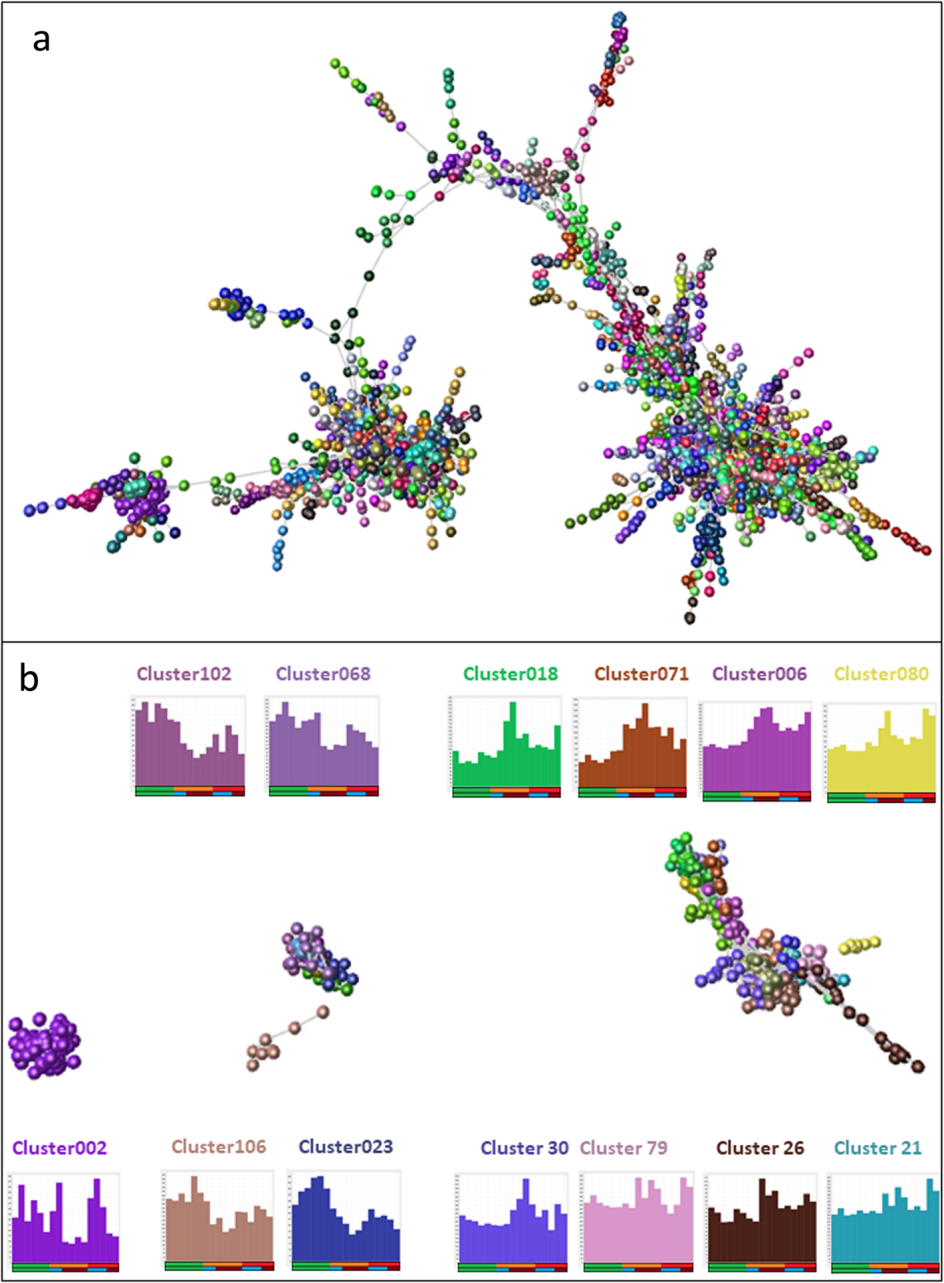
Gene-to-gene analysis using BioLayout, showing relationship between genes. Nodes (spheres) represent genes and edges (lines) show correlation of greater than 0.90 between gene expression patterns across all samples, allowing the similarity of gene expression patterns across all samples to be examined. **a**. The largest element in the graph created by BioLayout from the expression profiles of genes across the breeds and disease status. Nodes of the same colour were allocated to the same expression cluster by the MCL clustering algorithm (inflation value 1.7) because they have similar expression patterns in the samples. **b**. Clusters showing differential expression according to sample type. The network layout is the same as for Fig. 2a but only the apparent differentially expressed clusters are shown. Histograms surrounding the network graph are coloured the same way as the nodes of that cluster and show the average expression of genes in the cluster. X axis shows the disease status of the valve; upper bar shows the grade of disease (Green − normal valves; Orange – Grade 3 diseased valves; red – Grade 4 diseased valves); lower bar shows the breed and valve status (green − normal valves; blue − non-CKCS diseased valves; red − CKCS diseased valves). Y axis shows average expression. Gene lists for these clusters and enlarged images of the histograms are presented in Additional file 1

To explore the transcriptomic differences between diseased valves from CKCS and other breeds and from normal valves, we used BioLayout to construct a gene co-expression network (GCN) [28] from the same expression data, using a threshold correlation coefficient of 0.9. The analysis included 7247 genes making 20,612 edges. Nodes within the network were then clustered based on the similarity of expression pattern using the Markov Clustering (MCL) algorithm at an inflation value of 1.7, as implemented in BioLayout. This is a hypothesis-free approach, where the number of clusters is not constrained. In this analysis we reviewed all clusters with at least 5 nodes (probesets with expression patterns correlated at r ≥ 0.9). As we have seen in other studies [29, 30] there was considerable individual dog-specific variation and many clusters were driven by high expression of a subset of genes in a single or small number of individuals, independent of disease status. These clusters were not analysed further. However, there were also clusters showing overall high or low expression in the majority of diseased valves and a small number where expression was high or low only in CKCS. This was consistent with the sample-to-sample analysis and suggested that there were indeed sets of differentially expressed genes that distinguished the normal and diseased valves. The largest component of the network graph is shown in Fig. 2a and clusters that demonstrate differences between diseased and normal valves are shown in Fig. 2b. Histograms indicate the average expression of genes in the clusters; gene lists for these clusters and enlarged images of the histograms are presented in Additional file 1, Table S1.

Having identified clusters that showed expression patterns associated with disease status (Fig. 2b), we searched them for proliferation genes and found that there was no association of cell division markers [31] with disease or breed status, suggesting that perturbed patterns of cell division may not be a consistent feature of MMVD in either CKCS or other breeds. In addition, mitochondrial genes [32, 33] were spread amongst a number of clusters and showed no association with breed or disease status, suggesting that mitochondrial dysfunction does not make a major contribution to MMVD. Since immune response had been identified as altered in previous studies [18, 19] we also looked for clusters associated with changes in expression of immune/inflammatory genes [34]. Consistent with reports of an immune involvement in MMVD, a small number of macrophage and T-cell genes were in cluster011 (higher in all diseased valves; Additional file 1, Table S1). However, most of the genes associated with immune function were found in different clusters and showed no correlation with disease or breed status.

As mentioned above, the majority of the clusters had expression patterns driven by a single sample, and would not reveal functions likely to be informative for MMVD aetiology in general. We therefore subjected only the genes within the disease associated clusters shown in Fig. 2b to enrichment analysis using DAVID (see Methods). Cluster002, which was a large group of genes apparently down-regulated in CKCS, was analysed separately, while the genes in other down-regulated clusters and the up-regulated clusters were each pooled because they showed similar average expression patterns and were close to each other in the network. This ensured that the groups were of sufficient size for meaningful analysis **(**Additional file 1, Table S1).

For Cluster002, which contained genes that were lower in CKCS valves than those from other diseased dogs or normal dogs, there was enrichment of Biological Process (BP) terms related to muscle structure and activity, cardiac conduction and calcium ion release. For Cellular Component (CC), terms related to sarcolemma were enriched. The top 10 GO terms are presented in Additional file 1, Table S2. For the group of clusters in which genes were generally down-regulated in all diseased valves, DAVID GO enrichment analysis found enrichment for extracellular matrix and cell surface terms. For the clusters which contained genes that were up-regulated in diseased valves, there was enrichment of GO terms related to inflammatory response, monocyte chemotaxis and extracellular exosome.

### Comparison of CKCS diseased valve transcriptome with normal valve transcriptome

The BioLayout analysis identified groups of genes that appeared to be up- or down-regulated in diseased valves, as well as a cluster of genes that were down-regulated only in CKCS valves. The analysis revealed that there were many genes showing idiosyncratic sample-specific expression patterns, which may have concealed some meaningful differences and resulted in the paucity of significant GO term enhancement. We therefore generated lists of differentially expressed genes (DEG) with the Affymetrix Transcription Analysis Console, using a one-way between-subject ANOVA (unpaired), which takes into account variance among samples in calculating the *p*-value and determines a false discovery rate (FDR) to allow for multiple comparisons. A threshold FDR q-value of 0.05 was used in the analyses.

**Fig. 3:**
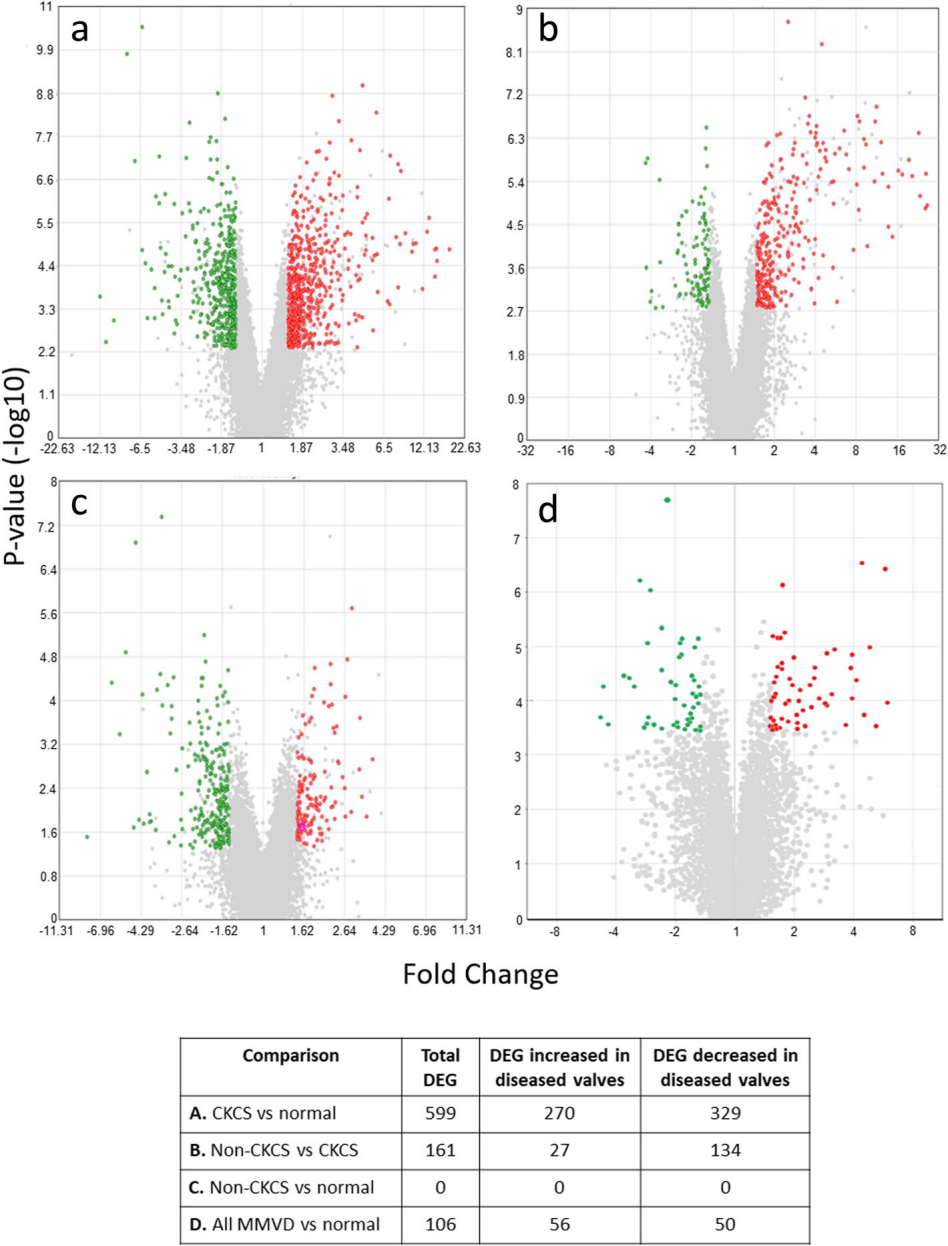
Volcano plots of differentially expressed genes comparing CKCS, non-CKCS, all diseased valves and normal dog valves. Red dots represent genes that show increased expression, green represent genes that show decreased expression and grey dots represent genes which did not pass the differential expression criteria. The X-axis shows fold change value and the Y-axis shows *p*-value. Central vertical line shows 0 fold change with negative fold changes on the left and positive fold changes on the right. Only fold changes of at least ±1.5 are shown. **a** CKCS vs Normal with FDR correction (q-value < 0.05). **b** non-CKCS vs CKCS with FDR correction (q-value < 0.05). **c** Non-CKCS vs Normal with no FDR correction (p-value < 0.05). **d** All diseased valves vs Normal with FDR correction (q-value < 0.05). The table shows numbers of DEG which met the stringent criteria (fold change ±1.5, FDR q-value < 0.05) are shown for each comparison

To confirm the findings of the GCN analysis, an initial comparison was made between CKCS diseased valves and normal valves. Using a fold change of at least 1.5 in either direction and an FDR q-value < 0.05, transcripts detected by 755 probesets were differentially expressed, representing 599 annotated genes and a number of unannotated probesets. Two hundred and seventy-one annotated genes were higher in CKCS (up-regulated) and 328 genes were lower in CKCS (down-regulated) (Fig. 3a, full list of genes in Additional file 2, Table S3). These 599 genes were analysed using DAVID GO enrichment analysis. For the down-regulated genes, there was enrichment of similar GO terms to those found for Cluster002 of the GCN analysis, as shown for the top 10 enriched GO terms in Additional file 2, Table S4. The enriched GO terms were related to cardiac muscle cell function and structure and calcium channel activity. For up-regulated genes terms related to immune response and ERK1/ERK2 activity were listed.

### Comparison of CKCS diseased valve transcriptome with transcriptome of diseased valves from other breeds

The sample-to-sample analysis suggested that gene expression in CKCS diseased valves was different from that in diseased valves from other breeds. Therefore we next compared the two sets of diseased valves. One hundred and sixty one annotated genes were differentially expressed, 27 with higher expression in CKCS valves than other diseased valves and 134 with lower expression in CKCS valves (Fig. 3b, Additional file 3, Table S5). For the genes that were lower in CKCS diseased valves than other breed diseased valves, the GO terms were similar to those distinguishing CKCS from normal valves, with an emphasis on cardiac muscle structure and function (Additional file 3, Table S6). A single GO term was enriched for the genes that were higher in CKCS valves than other breed diseased valves.

Eighty-one genes were downregulated in CKCS diseased valves compared both to normal valves and to diseased valves from other breeds, suggesting that these represent a breed-specific effect, which may or may not be related to the disease. Similar GO terms were enriched in this set of down-regulated genes, primarily terms associated with cardiac development and function (Additional file 4, Table S7 and Table S8). This suggests that CKCS have abnormalities of expression of genes involved in cardiac development and function which may be related to the early onset of MMVD. Nineteen genes were up-regulated in CKCS compared with both normal valves and diseased valves from other breeds. A single GO term was enriched, as for the comparison between CKCS and other breed diseased valves (Additional file 4, Table S8).

### Comparison of diseased valve transcriptome with normal valve transcriptome

The BioLayout sample-to-sample network showed that the non-CKCS diseased valves were close in gene expression pattern to the normal valves (Fig. 1c), and there were no significant DEGs when comparing these two groups at the stringency used for the other comparisons (FDR q-value < 0.05). Consistent with this, the volcano plot for this comparison showed that the fold changes were lower, the *p*-values were higher and the genes were less scattered than in the other plots, supporting the observation that the normal and non-CKCS diseased valves were closer in gene expression pattern than either were to the CKCS valves. Relaxing the stringency of the analysis (unadjusted *p* < 0.05, no FDR correction; Fig. 3c) showed that there were 278 differentially expressed genes, of which 166 were higher in the diseased valves and 112 were lower. For this lower stringency set, there were several enriched GO terms that overlapped with the set produced comparing CKCS and normal valves.

The network cluster analysis showed that there were a number of genes where the majority of diseased valves were different from the normal valves. To examine further whether there were genes that were differentially expressed in both sets of diseased valves, we generated a list of DEGs comparing all diseased valves with normal valves (FDR q-value < 0.05, fold change at least 1.5 in either direction) (Additional file 5, Table S9). One hundred and six genes were differentially expressed, 50 with lower expression and 56 with higher expression in diseased valves than normal valves (Fig. 3d). Enrichment of GO terms for the down- and up-regulated genes is shown in Additional file 5, Table S10. For the up-regulated genes, terms related to skeletal system and mesenchyme migration were found, consistent with the increased EndoMT in the diseased valve. For the downregulated genes, the term calcium ion binding was enriched, supporting the idea that calcium homeostasis is perturbed in MMVD.

### Pathway analysis of differentially expressed genes

The online Ingenuity Pathway Analysis tool (IPA) includes a database of genes/proteins in pathways built mostly from human and laboratory animal data. Analysis with IPA identified 77 canonical pathways when comparing the transcriptome of normal valves to CKCS valves, 28 when comparing CKCS valves to non-CKCS diseased valves and 56 when comparing all diseased valves with normal valves. The top three pathways for each comparison are shown in Table 2 and the top four upstream regulators for each dataset comparison, with their associated Z-score indicating predicted activation or inhibition, are shown in Table 3. Of particular note are changes in *calcium signaling* in the CKCS compared to both normal and non-CKCSs datasets (Tables 3 and 4). For *calcium signaling* there were 23 DEGs (17 lower in CKCS) comparing CKCS with normal, 10 DEGs (all lower in CKCS) comparing CKCS and non-CKCS and 5 DEGs (1 lower in diseased valves) comparing all diseased valves to normal. Shared DEGs included genes associated with calcium homeostasis, non-canonical TGFβ signalling pathways (ERK1/2, IP3, RhoGTPase), cytoskeleton, muscle contraction (including cardiomyocytes), and the calcineurin/NFAT (nuclear factor of activated T cells) pathway. IPA analysis also found that *hepatic fibrosis/hepatic stellate cell activation* was changed in CKCS and all diseased valves compared to normal. This association was also found in the lower stringency analysis of normal valves with non-CKCS diseased valves. In the *hepatic fibrosis/hepatic stellate cell activation* gene dataset DEGs included genes associated with collagen homeostasis, cytoskeleton and cell growth and differentiation. Fibroblasts from different organs have different phenotypes and the relevance to mitral VICs is unclear.

**Table 2:** Top three canonical pathways associated with each dataset. The number of genes altered in each pathway as well as the total number of genes changed in each pathway is shown

Analysis	Canonical Pathway	Up	Down	Gene changes in pathway	***p***-value
CKCS vs Normal	**Calcium signaling**	**6**	**17**	**23/179**	**7.943E-11**
	Hepatic fibrosis/Hepatic stellate cell activation	8	12	20/183	2.988E-08
	Axonal guidance signaling	11	16	27/452	1.995E-05
CKCS vs non-CKCS	**Calcium signaling**	**0**	**10**	**10/179**	**6.456E-06**
	LPS/IL-1 mediated inhibition of RXR function	2	5	7/168	0.0009
	Gluconeogenesis I	0	3	3/22	0.001
All diseased vs normal	Paxillin signaling	3	1	4/108	0.0006
	**Calcium signaling**	**4**	**1**	**5/179**	**0.0009**
	STAT3 pathway	4	0	4/135	0.001

Of note is change in calcium signalling comparing CKCS to the other two data sets highlighted in bold. The P-value score shows the strength of association of the gene list to the pathway

**Table 3:** The top four upstream regulators associated with the differentially expressed genes lists for each dataset

Analysis	Upstream regulator	Molecule type	Activation Z-score	P-value
CKCS vs Normal	F2	Peptidase	1.503	2.2E-12
	TNF	Cytokine	1.892	2.18E-11
	AGT	Growth factor	2.018	1.15E-10
	TGFB1	Growth factor	1.486	9.03E-10
CKCS vs non-CKCS	MEF2C	Transcription regulator	−3.087	1.11E-09
	MYOCD	Transcription regulator	−2.768	1.33E-07
	2,3 butanedione monoxime	Chemical drug	−1.4	3.71E-07
	DNMT3A	Enzyme	1.667	6.27E-07
All diseased vs Normal	NOTCH4	Transcription regulator	1.777	6.33E-10
	MED28	Other	−1.957	6.32E-09
	HEY1	Transcription regulator	−1.547	2.61E-08
	MYCOD	Transcription regulator	2.571	4.6E-08

For each upstream regulator, the molecule type, Z-score and P-value are given. The activation Z-score is used to infer likely activation states of upstream regulators based on comparison with a model that assigns random regulation directions. https://www.qiagenbioinformatics.com/products/ingenuity-pathway-analysis/

**Table 4:** Gene expression changes associated with calcium signalling and hepatic fibrosis/hepatic stellate cell activation canonical pathways

Datasets	Gene name
**Calcium Signalling**	
CKCS vs Normal	***ACTA1****, ACTA2, AKAP5,* ***ATP2A2****,* ***CASQ, GRIN2A****,* ***GRIA3,*** *HDAC9,* ***MYH7****,* ***MYH7B****, MYH1,* ***MYL4****,* ***NFATC1, RCAN2****,* ***RYR2****,* ***SLC8B1****,* ***TNNI3,*** *TPM2,* ***TRDN***
CKCS vs non-CKCS	***ACTA1****,* ***ATP2A2****,* ***CACNA1G****,* ***CACNA1H, CASQ2****,* ***MYH7, RCAN2****,* ***SLC8B1****,* ***TNNI3****,* ***TRDN***
All diseased vs Normal	*ACTA2, HDAC9, MYH11, TPM2,* ***TRPC5***
**Hepatic fibrosis/hepatic stellate cell activation**	
CKCS vs Normal	*ACTA2, CCL5,* ***COL6A3****,* ***EDNRA****, IL1RL1,* ***KDR, LAMA1****,* ***MYH7****, MYH11,* ***MYH7B****,* ***MYL4****,* ***NGFR****,* ***PDGFR****, SERPINE1,*
All diseased vs Normal	*ACTA2,* ***COL11A2,*** *MYH11, SERPINE1*

Down-regulated genes are shown in bold

A curious observation comparing CKCS to normal was IPA predicted up-stream regulator analysis tended to be activated (positive activation Z-score in IPA), but the predicted regulators tended to be inhibited in CKCS relative to non-CKCS (negative activation Z-score). However, these differences in themselves are not the main biological interest, but which regulators are predicted to be affected and whether activated or inhibited. For CKCSs compared to normal there were 1831 molecules with F2 (prothrombin) signalling pathway having the strongest association, but also TNF and TGFβ1 signalling featured in the top four upstream regulators. Comparing the two disease datasets, 377 molecules were associated, with the top upstream regulator being myocyte-specific factor 2c (MEF2C). The down-stream effects, as predicted by IPA, for TGFβ1, MEF2C and F2 (prothrombin) are shown in Additional file 6, Figure S1 A-C.

The top five disease and function annotations were assigned to the datasets by IPA and an illustrative graphical representation is shown in the Additional file 7, Table S11 and Figure S2. Comparing the datasets, these annotations generally matched to the same general themes, but identifying the subtle differences required examination of the graphic representations, with the illustrative example for *Skeletal and Muscular Disorders and Developmental Disorder and Hereditary Disorder*, comparing CKCS and non-CKCS shown in Additional file 7, Figure S2.

## Discussion

We have recently shown that the transcriptome of the mitral valve changes as MMVD advances from the normal valve to the severely diseased Whitney grade 4 valve [21]. We found that TGFβ was the dominant signalling pathway controlling pathogenesis, consistent with findings in cultured valve interstitial cells [22]. The current study has identified gene expression differences in mitral valves of CKCS with severe valve pathology compared to valves from age and disease severity-matched non-CKCS dogs. These differences may be associated with the earlier onset of severe MMVD in the CKCS or may be an unrelated feature of the breed. Given the high breed-specific prevalence of MMVD in CKCS and the involvement of many of the pathways identified in the development and function of the cardiovascular system, it is possible that these differences influence the aetiology of MMVD in CKCS, although the effects of these pathways on MMVD development will need to be confirmed with functional studies. However, there are several limitations to the study. Firstly, all diseased dogs were older than the normal dogs and differences could simply be due to an age effect unrelated to MMVD status. Secondly, the other breed diseased valves and the normal valves came from several breeds of dogs, including some mixed breed animals, and the differences from CKCS valves could reflect the greater genetic similarity of the CKCS in contrast to the greater admixture of the other dogs [35]. Examining gene expression in normal and diseased age-matched CKCS valves would be ideal, but obtaining healthy valves from elderly dogs is not feasible due to the ubiquity of the disease in older CKCS. Similarly, comparison of normal valves from young CKCS with diseased valves from older CKCS would be interesting, but our study relies on samples from client owned dogs, and young CKCS with healthy valves are rarely euthanased. Gene expression could also be examined in age matched valves of another MMVD-predisposed breed with a similar level of genetic diversity. However the early onset and high prevalence of MMVD in CKCS means that it would be difficult to find a matched breed. Nonetheless, it is possible to draw some conclusions from our study.

Among the genes that had lower expression in CKCS valves than non-CKCS diseased valves or normal dog valves were a range of cardiomyocyte-related genes including *CASQ2* (calsequestrin), *TNNI3* (troponin I type3) and *RYR2* (ryanodine receptor 2), and various myosin genes. A striking feature of the GO term analysis was the reduced expression of genes involved in cardiac development and function in the CKCS valves. There may be a more general effect on the cardiovascular system of the CKCS. There were clear similarities in gene enrichment analysis and GO terms comparing both diseased groups to normal, including genes recognised as hallmarks of canine MMVD, such as *ACTA2* (encoding α-SMA) and *HTR2B* (encoding 5HT2B receptor) [18, 27]. This likely reflects the global gene signature typical of MMVD where there is aberrant extra-cellular matrix remodelling as a consequence of changes in TGFβ signalling [18, 19, 21, 36]. The other shared DEGs, in particular those coding for the myosin heavy chains and growth factors, suggest the involvement of TGFβ non-canonical signalling pathways affecting down-stream signals including MEK, ERK1/2, IP3 and RhoGTPase [37]. All these pathways have regulatory roles in stress fibre formation, EndoMT and apoptosis, and can be induced by the TGFβs, 5HT and Ca^++^ signalling. TGFβ1 was one of the top upstream regulators in the CKCS valves, which would suggest TGFβ1 is an important driver of MMVD pathogenesis in dogs.

In the previously published study comparing diseased CKCS valves with normal valves using the Affymetrix Canine Gene 1.0ST Array, similar changes in gene ontology and gene networks were found, including regulation of EndoMT and caveolar-mediated endocytosis, but calcium signalling as a dysregulated canonical pathway was not reported [18]. In that study IPA identified only 33 canonical pathways compared to 77 in the current study.

The identification of the *hepatic fibrosis/hepatic stellate cell activation* pathway in diseased valves might appear problematic to explain given that it refers to hepatic pericytes and that fibroblast phenotypes differ between tissues. However, this term encompasses signalling pathways relevant to the broad interstitial/myofibroblast cell lineage, which activated myofibroblasts, and this observation fits with what is already known about MMVD and the role of activated myofibroblasts in ECM remodelling and disease pathogenesis [23, 38–40]. Of interest was the identification of GO terms and canonical pathways and upstream regulators exclusive to the CKCS group. These included the canonical pathway *calcium signalling* and upstream regulator F2 (prothrombin), and various cardiovascular and immune-related GO terms. The potential role of immunity in MMVD might be difficult to explain as there is currently no evidence of inflammatory cell involvement in canine MMVD [23, 38]. There is however, evidence for inflammatory changes in the human MMVD and the CKCS breed does appear to be pre-disposed to a range of inflammatory conditions [20, 23, 38, 41, 42]. Network analysis using IPA has identified genes associated with “inflammation”, but this likely refers to pathways shared with other functions such as cell signalling, cell migration and EndoMT, since many of the same genes can be associated with immune responses [18, 19, 27]. Furthermore, expression of interleukins, chemokines and growth factors can be affected by the same up-steam regulators that control TGFβ signalling pathways.

F2 (prothrombin) was identified as the top upstream regulator in diseased CKCS valves, compared to normal valves, and IPA predicted pathway activation, with effects on a relatively large range of genes in the extra-cellular space, plasma membrane, cytoplasm and nucleus, including *ACTA2* (Additional file 6, Figure S1 C). While typically associated with blood coagulation, prothrombin/thrombin also has a pro-inflammatory role and down-stream regulatory effects on endothelial, smooth muscle and fibroblast (interstitial) cells, and can both induce and inhibit TGFβ1 signalling in a context specific manner [43]. However, the reported CKCS predisposition to platelet dysfunction and macro-thrombocytopenia is well recognised, and macro-thrombocytopenia has been associated with early MMVD development in humans [7, 10, 12]. Considering these effects, and what is known about MMVD pathology, a potential role for prothrombin/thrombin in CKCS MMVD pathogenesis can be postulated, possibly through platelet interaction and enhanced 5HT signalling [15, 44, 45].

Changes in expression of cardiomyocyte-related genes were found in the CKCS dataset, including *MB* (myoglobin), *CASQ2* (calsequestrin2), *NEBL* (nebulette) and various actin and myosin genes. While the changes in calcium signalling and *MEF2C* expression might reflect global changes in cell signalling and ECM remodelling, they might also be due to changes in the cardiomyocyte content of the tissue samples. Care was taken to ensure the same dissection protocol, removing atrial myocardium and the annular attachment, was used for each valve. Nevertheless, in whole valve samples atrial cardiomyocytes can be found extending a variable distance into the valve, and this extent declines in diseased leaflets [46–49]. Morphological studies are needed to determine if there are differences in CKCS valve myocardium content compared to non-CKCS that may explain some of these transcriptomic differences. Indeed, there is emerging interest in valve contraction mechanics and how this is affected by or contributes to the diseased state, and in this context the relative contribution of valve cardiomyocytes needs to be considered [50].

The myocardial gene *MEF2C* (the transcription factor myocyte-specific factor 2c) was the top upstream regulator when CKCS were compared to non-CKCS diseased valves and IPA predicted pathway inhibition, with effects on a relatively small group of cytoplasmic and nucleus genes including *CASQ2* (Additional file 6, Figure S1 B). MEF2C is important in myogenesis and cardiomyocyte development, regulating cardiac alpha-actin and alpha myosin heavy chain [51]. However, as a transcription factor, it also regulates the expression of the ECM protein cartilage link protein 1 (*HAPLN1* gene) in VICs during valve development and has a wide range of other effects, including in the EGF/EGFR and the apelin signalling pathways, that contribute to non-canonical TGFβ1 signalling, proper development of megakaryocytes and platelets, and cell migration and differentiation [51].

*Calcium signalling* was identified as the top canonical pathway in the CKCS dataset, and transcription factors in the top upstream regulator list and GO terms related to cardiac muscle contraction, control and differentiation, were down-regulated in CKCS diseased valves. Understanding how down-regulation of cardiac muscle, smooth muscle-related and calcium binding pathways impact on CKCS MMVD may provide insight into the early-onset pathogenesis in CKCS. For example, 5HT signalling through Gq protein-coupled receptors (such as 5HTR2B) causes an influx of calcium into the cell as part of 5HT downstream signalling pathways, and there is consistent and significant increased *5HTR2B* expression in the canine mitral valve transcriptome [15, 18, 19, 27]. In the light of the data presented here, the recent identification of the beneficial effect of the calcium sensitizer and positive inotrope pimobendan in protecting against progression to heart failure is an interesting finding [52].

The results of this study provide hints about the genes in which variants might be related to the severe early onset of MMVD in CKCS. To validate these results a number of approaches could be taken. As part of on-going studies we are examining valve morphology to quantify the any differences in cardiomyocyte content in the CKCS. Since it is not possible to obtain samples from young/healthy client-owned dogs, further investigation of other breeds susceptible to MMVD could determine if changes seen are breed-specific. We have validated, and currently are using, a robust primary VIC cell culture system for which we have transcriptomic data for cells derived from non-CKCS, and this could be used to examine further CKCS-specific differences [22, 53]. Loss/gain of function studies for specific genes of interest could be undertaken to see if they affect the valvular cell phenotype. Lastly, more targeted examination of the calcium signalling and other pathways identified could be undertaken, examining both gene and protein expression in CKCS valve tissue and cell cultures.

The notable limitations of this study include the small sample size, the age disparity between normal and diseased animals and the genetic dissimilarity of the CKCS to the other dogs. Difficulties in age-matching are an accepted confounding factor for MMVD research considering the ubiquity of valve pathology changes in all elderly dogs, some of which may be associated with healthy aging. CKCS have a distinct genetic profile [35] and this may account, in part, for the disparity in DEG numbers comparing CKCS with the two other groups. Studies are needed to determine whether this differential gene expression underlies the susceptibility of the CKCS to develop the disease sooner and to progress more rapidly than other dogs, bearing in mind that they might not have life-long differential expression [24]. Lastly, the more stringent FDR analysis could not be applied to the normal and non-CKCS analysis. This likely reflects their genetic heterogeneity and difference in baseline expression of genes, and in that circumstance is an accepted limitation of genomic profiling, with similar problems found when examining human populations [20, 54]. Relaxing the strict FDR threshold revealed candidate genes and pathways that were enriched in this comparison, consistent with the observations of the higher stringency comparisons and supporting the need for further analysis of these candidates.

## Conclusions

This study has identified interesting differences in the transcriptomic profile of CKCS mitral valves compared with non-CKCS valves, all with severe MMVD. In particular we have identified differences in expression for genes associated with cardiomyocytes including CASQ2, TNNI3 and RYR2. Studies are needed to determine if these gene expression changes simply reflect differences in valve cardiomyocyte density, which might then affect valve mechanics, valve coaptation and pathology development. Alternatively, the gene expression changes may impact in a CKCS-specific manner on valve cell function and ECM synthesis and remodelling. These results provide the basis for further studies to examine the specific gene and signalling pathway changes and their contribution to disease pathogenesis, and any breed-specific susceptibility.

## Methods

### Aims, design and setting of the study

The aim of this study was to determine whether expression patterns were different in CKCS diseased mitral valves compared to non-CKCS dogs. Valve samples were collected from dogs were presented to the Hospital for Small Animals, Royal (Dick) School of Veterinary Studies, the University of Edinburgh. Euthanasia had been requested by the owners because of terminal conditions or intractable conditions which impacted severely on their pets welfare. No dogs were euthanased for the purpose of the study and all tissue was collected and used with full informed written owner consent, and with institutional ethical approval (Veterinary Ethics in Research Committee).

### Tissue samples

Details of the dogs used in this study are shown in Table 1. All valves were scored by two of the authors (GRM & BMC). Diseased valves had changes typical of Whitney grade 3 or 4 (severe to very severe disease) [2]. Normal dogs had no evidence of any disease, and mitral valves were considered to be normal on gross inspection. Valves were collected shortly after euthanasia as previously described (intra-venous pentobarbitone overdose) [18], washed gently in warm phosphate buffered saline (PBS), immediately placed in RNAlater (Invitrogen USA) and stored for future RNA extraction [18]. Any attached atrial myocardium, annulus and chordae were removed, and the remaining whole valve was used for RNA extraction. Dogs with advanced disease were on a combination of standard medication for the treatment of congestive heart failure and any effect this might have on valve gene expression is unknown.

### RNA extraction, quantification, quality control and transcriptomic profiling

RNA was extracted from the whole valve (anterior and posterior leaflet) following a standard protocol as previously described [18]. RNA extraction and DNA digestion were performed using the Qiagen RNeasy mini kit (Qiagen, Germany) according to the manufacturer’s instructions. Total RNA was eluted in 30 μl nuclease free water and stored at − 70 °C. Quantification of RNA was performed by spectrophotometry in a NanoDrop™1000 (Thermo Scientific), measuring absorbance at 260 nm wavelength in 1 μl of extracted RNA solution, and absorbance ratios of 260/280 and 260/230 were analysed to check for impurities. The ratio of ribosomal 28S to 18S RNA was measured to assess for degradation using the Agilent RNA Screentape system and Agilent 2200 tapestation analyser (Agilent Technologies, USA) according to the manufacturer’s instructions. RNA integrity number (RIN) was then calculated with RIN ≥7 being taken as optimal for transcriptomic analysis. The Affymetrix GeneChip™ Canine Gene 1.1 ST Array plate was used for transcriptomic profiling. Arrays were run by Edinburgh Genomics, University of Edinburgh, UK. The Affymetrix Expression Console (Build 1.4.1.46) was used to normalise the data from the generated .cel files and for quality control. An annotated file of expression results was generated and filtered for all genes with a maximum relative expression level of less than 15, consistent with the background level detected during the quality control process. This filtered set of genes was used for subsequent expression and enhancement analysis.

### Network analysis and functional clustering of canine valve samples

The network analysis tool BioLayout-3.4 (http://biolayout.org) [55] was designed for the visualisation and analysis of network graphs from large datasets. BioLayout clusters data based on similarity of gene expression pattern with nodes representing a data point and edges the relation between nodes, using the Fruchterman-Rheingold algorithm. BioLayout was used to examine expression of genes across the grades of valve disease. In a sample-to-sample analysis (similar to a principal components analysis) nodes represent samples and the network layout shows the similarity of samples based on the expression of all genes in the sample. For the sample-to-sample comparison of mitral valve transcriptomic data using the filtered set of genes (relative expression ≥15 in at least one sample), a Pearson correlation coefficient of r ≥ 0.98 was used as it was the highest that included all samples. Meta-data (grade of disease, age, sex, breed) associated with the dataset were then examined to see if any parameter accounted for the network layout.

Gene-to-gene analysis with BioLayout was used to generate a gene co-expression network (GCN) where nodes represent genes and edges the correlation between them at or above the chosen threshold. The network layout shows the similarity of gene expression patterns across all samples. For the network layout a high stringency threshold r value of 0.9 was used. Subsequent analysis using the Markov clustering algorithm (MCL) [56] identified groups of highly connected genes within the elements of the network. The inflation value was set at 1.7 to control granularity of the clusters.

### Differentially expressed genes

The Affymetrix transcriptome analysis console (TAC, version 3.1.0.5) was used to perform unpaired one-way analysis of variance and detect differentially expressed genes (DEG). DEG lists were created for genes with a fold change of > 1.5 or < − 1.5. A Benjamini-Hochberg false discovery rate (FDR) correction (Q-value < 0.05) was applied. Annotation information for un-annotated transcript probes was found in TAC through an interface with the Affymetrix online browser. Gene lists were then used for gene enrichment analysis.

Reverse transcriptase quantitative PCR (RT-qPCR) was undertaken using the Takyon 2X low Rox SYBR green mastermix dTTP blue (Eurogentec, Belgium) to validate the microarray data and included the following genes; *ACTA2*, *HTR2B*, *TAGLN*, *ACTG2*, *SLIT3*, *CDKN2A*, *SLC10A6*, *CILP*, *MMP12*, *ADAMTS5* and *ADAMTS19*. Primer sequences are shown in Table 5. RT-qPCR showed equivalent direction and magnitude to the results with the microarray for these genes.

**Table 5:** Primer sequences for selected genes used in RT-qPCR to validate the microarray data

Gene Symbol	Forward Primer Sequence	Reverse Primer Sequence
*ACTA2*	5’CGGCTACTCCTTTGTGACG3’	5’CGTGGCCATCTCGTTCTC3’
*HTR2B*	5’CCAATCCAGGCCAATCAAAG3’	5’CAGGTGATGTTGCTTGGGTT3’
*TAGLN*	5’GACATGTTCCAGACCGTCGA3’	5’CAATGACGTGCTTTCCCTCC3’
*ACTG2*	5’TGCCAACAATGTCCTTTCCG3’	5’GCCTCCAATCCAGACTGAGT3’
*SLIT3*	5’CTGACAAGGACAACGGCATC3’	5’CCCATCATTCACCGTCTCCA3’
*CDKN2A*	5’CATGTTGGCTCAGAATCGGG3’	5’CTCACGTCCAAGGCACAAAA3’
*SLC10A6*	5’GCTGTTGGATGGGTTTCTCA3’	5’TCCAAGAAAGCACCAGTCTCT3’
*CILP*	5’TGCTCCAATTATACCGTGCG3’	5’CAGAACACTTGCTCCAGGGA3’
*MMP12*	5’GACACAATTCATGGACCCTGG3’	5’TCAAATACGTCAGGTCCTTGGA3’
*ADAMTS5*	5’GTTCCCAAATATGCAGGCGT3’	5’AGCTTCGAACCAATGATGCC3’
*ADAMTS19*	5′ GGACGGTGAGGTGTACTAAC 3’	5’ACTGCATTCCTTTACCACAGG 3’

### Gene enrichment analysis

Gene lists from the network analysis and the differentially expressed gene lists were analysed with the Database for Annotation, Visualisation and Integrated Discovery (DAVID v6.8; http://www.david.ncifcrf.gov) [57, 58]. DAVID collates the biological processes that are associated with genes in a list using the gene ontology (GO) terms. Gene lists deriving from differential expression analysis or BioLayout clustering were uploaded to DAVID for analysis. GO terms were selected for biological processes (GOTERM_BP_DIRECT), cellular components (GOTERM_CC_DIRECT) and molecular function (GOTERM_MF_DIRECT). The reference list was from *Canis lupus familiaris* and enrichment was assessed using Fisher’s exact test.

In addition, differentially expressed gene lists were uploaded, with related fold change, to the online Ingenuity Pathway Analysis (IPA) server (Qiagen, Germany) where core analysis was performed. This compared the submitted genes to all published literature and assigned reported attributes and pathways to them. From these canonical pathways, upstream regulators (activated, inhibited or activation state unknown) disease and biological functions, as well as other factors, were inferred by the software. These results were generated based on the number of genes in the submitted list that matched the genes reported in the literature to be involved in a certain process, with a statistical association calculated. Core analysis was performed with default settings on filtered gene sets.

## Supplementary information

**Additional file 1 **Gene lists (**Table S1**) and GO term enrichment analysis (**Table S2**) for clusters detected by GCN analysis with BioLayout.

**Additional file 2 **Lists of differentially expressed genes (**Table S3**) and GO term enrichment analysis (**Table S4**) for comparisons between CKCS and normal valves.

**Additional file 3 **Lists of differentially expressed genes (**Table S5**) and GO term enrichment analysis (**Table S6**) for comparisons between CKCS and other breed diseased valves.

**Additional file 4 **List of genes (**Table S7**) that were down-regulated in CKCS compared with both other breed diseased valves and normal valves and GO enrichment analysis (**Table S8**).

**Additional file 5 **Lists of differentially expressed genes (**Table S9**) and GO term enrichment analysis (**Table S10**) for comparisons between all diseased valves and normal valves.

**Additional file 6 **Graphical representations of predicted down-stream regulator effects identified by IPA (**Figure S1**).

**Additional file 7 **Disease and function networks identified by IPA (**Table S11**) and illustrative graphical representation (**Figure S2**).

## Data Availability

The datasets generated and analysed during the current study are available in the University of Edinburgh Datashare repository, 10.7488/ds/2754.

## Abbreviations

FDR: False discovery rate
IPA: Ingenuity Pathway Analysis
MMVD: Myxomatous mitral valve disease
