# Supplementary material for: Comparative transcriptomic profiling of myxomatous mitral valve disease in the cavalier King Charles spaniel
Source: BMC Vet Res. 2020 Sep 23;16:350. doi: 10.1186/s12917-020-02542-w (PMC7509937; doi:10.1186/s12917-020-02542-w)
Supplement: Supplementary file 1 — Additional file 1 Gene lists (Table S1) and GO term enrichment analysis (Table S2) for clusters detected by GCN analysis with BioLayout. [file 12917_2020_2542_MOESM1_ESM.pdf]

**Table S1.** Gene lists for 17 clusters and histograms of average gene expression for each cluster detected by GCN analysis with Graphia Pro.

Graphs show average normalised expression of genes in the cluster. Bars on X axis show status. Upper bar shows valve disease: blue - normal valves; yellow - grade 3 diseased valves; grey - grade 4 diseased valves. Lower bar shows breed: red - Beagle; green - mixed breed; pink - West Highland white terrier; salmon - Jack Russell Terrier; turquoise – CKCS

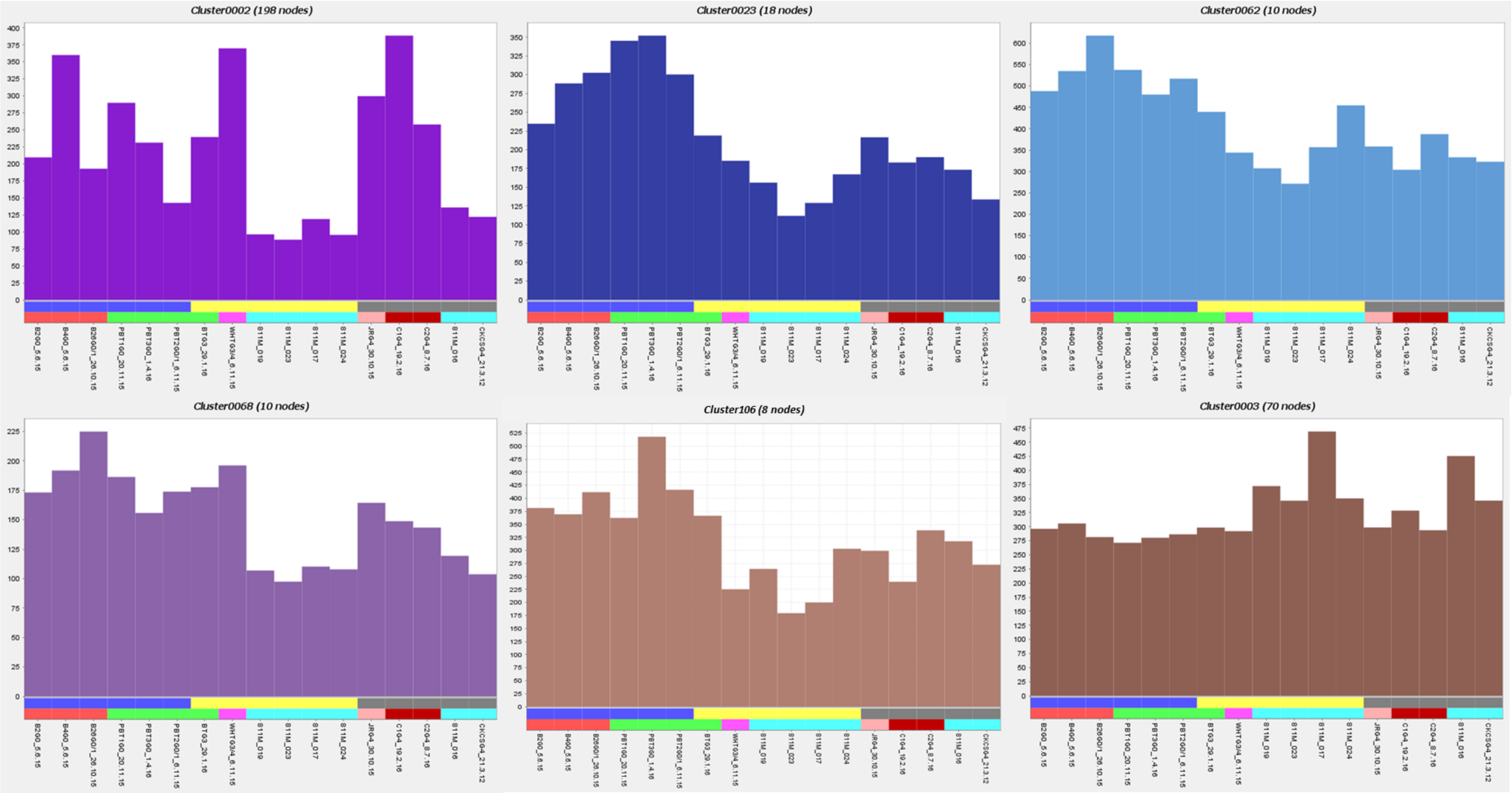

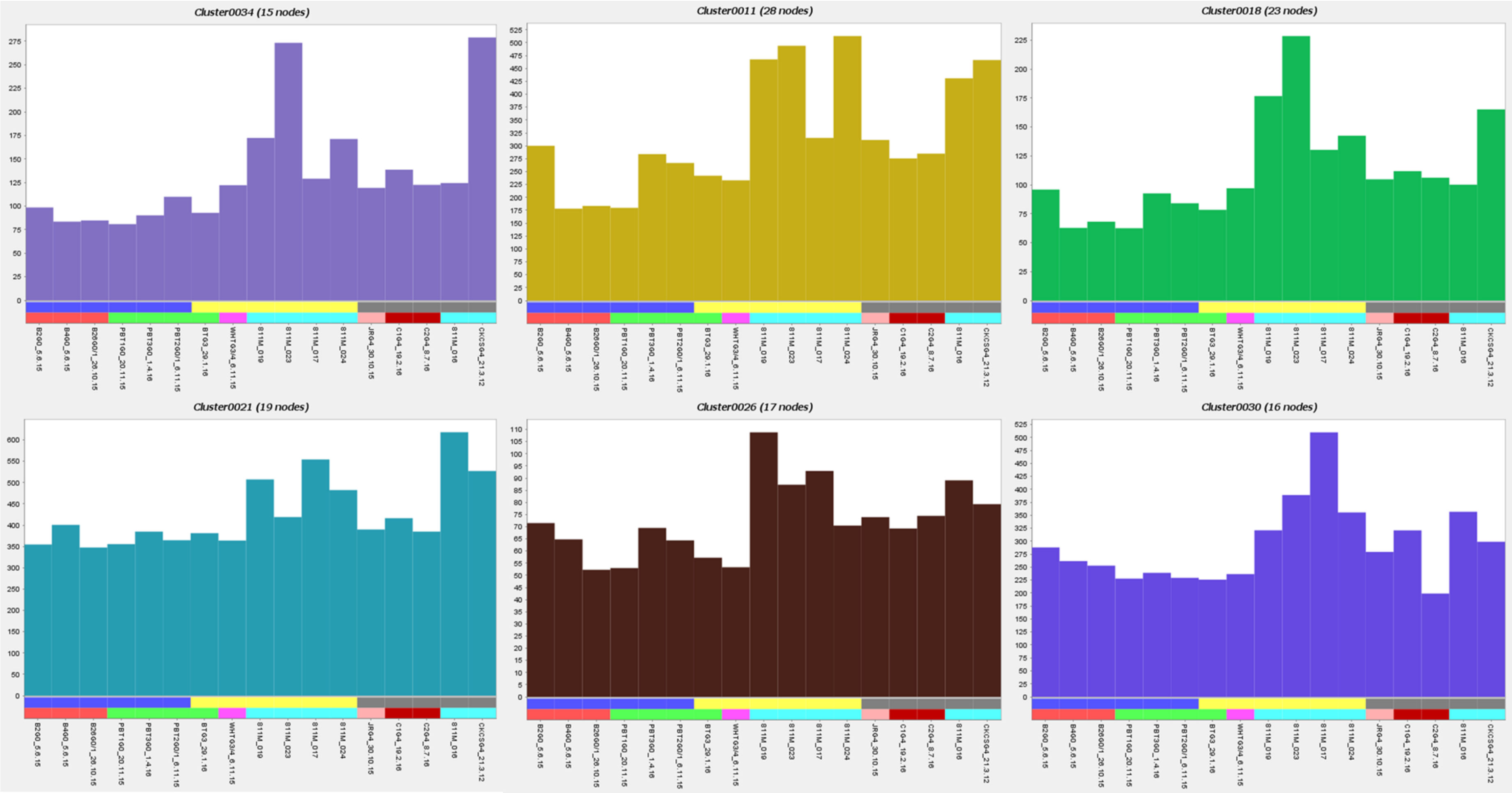

Cluster0034 (15 nodes)
Cluster0011 (28 nodes)
Cluster0018 (23 nodes)
Cluster0021 (19 nodes)
Cluster0026 (17 nodes)
Cluster0030 (16 nodes)

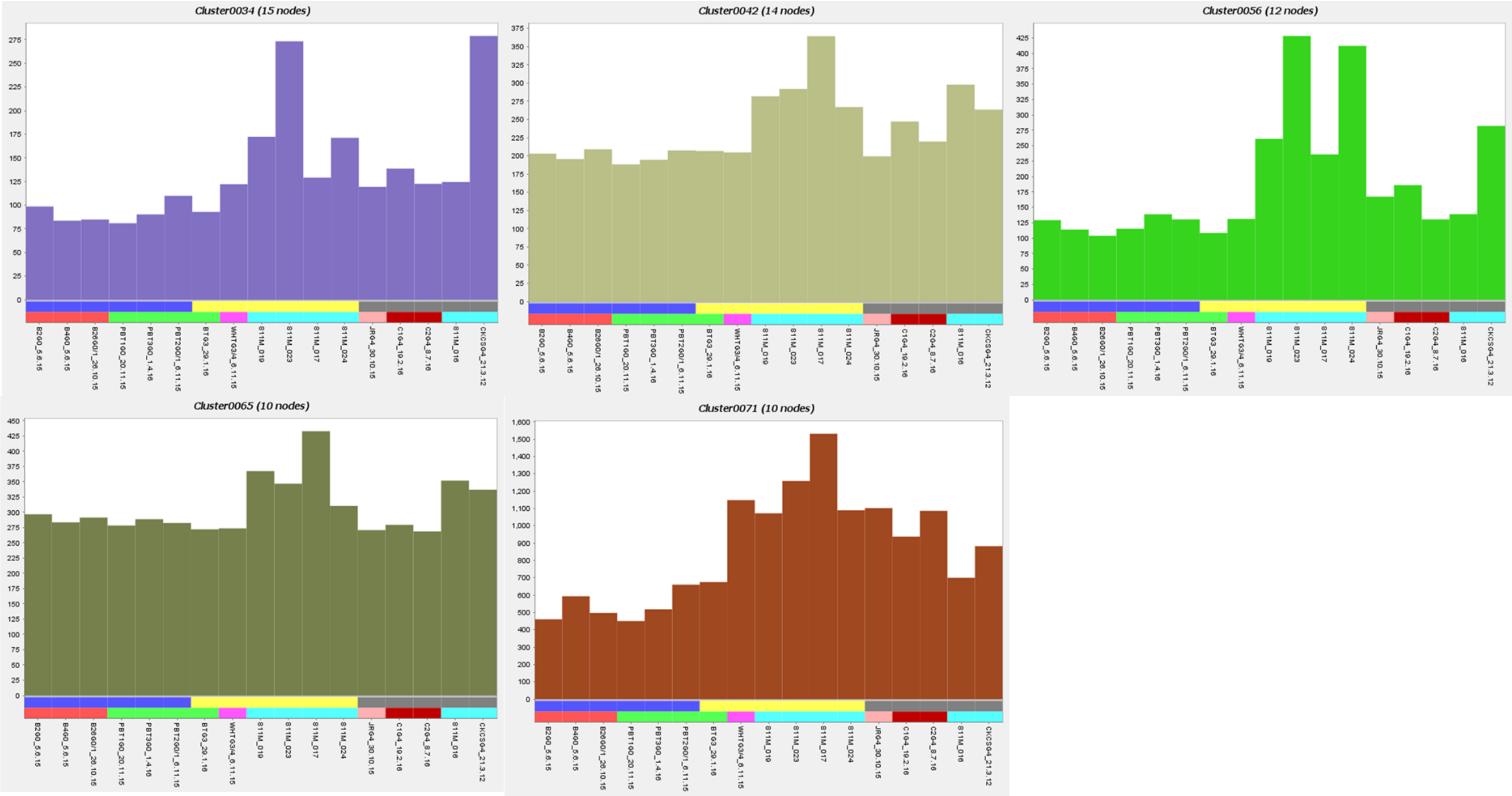
Cluster0034 (15 nodes)
Cluster0042 (14 nodes)
Cluster0056 (12 nodes)
Cluster0065 (10 nodes)
Cluster0071 (10 nodes)

| Low in CKCS only | | | | |
| --- | --- | --- | --- | --- |
| Name | Probeset | mRNA - Description | GO Biological Process Term | MCL_1.7_6 |
| --- | 14384960 | --- | --- | Cluster0002 |
| --- | 14390715 | adenylate cyclase 5 [gene_biotype:protein_coding transcript_biotype:protein_coding] | adenosine receptor signaling pathway // intracellular signal transduction // cAMP biosynthetic process // adenylate cyclase-activating G-protein coupled receptor signaling pathway // adenylate cyclase-activating dopamine receptor signaling pathway // adenylate cyclase-inhibiting dopamine receptor signaling pathway // locomotory behavior // cyclic nucleotide biosynthetic process // neuromuscular process controlling balance | Cluster0002 |
| --- | 14402907 | --- | --- | Cluster0002 |
| --- | 14402908 | --- | --- | Cluster0002 |
| --- | 14402909 | --- | --- | Cluster0002 |
| --- | 14429191 | Canis lupus familiaris troponin T type 2 (cardiac) (TNNT2), mRNA. | --- | Cluster0002 |
| --- | 14465648 | PREDICTED: Canis lupus familiaris actinin, alpha 2 (ACTN2), transcript variant X1, mRNA. | --- | Cluster0002 |
| --- | 14465658 | PREDICTED: Canis lupus familiaris actin, alpha 1, skeletal muscle (ACTA1), mRNA. | --- | Cluster0002 |
| --- | 14465660 | PREDICTED: Canis lupus familiaris actin, alpha 1, skeletal muscle (ACTA1), mRNA. | --- | Cluster0002 |
| --- | 14465662 | PREDICTED: Canis lupus familiaris actin, alpha 1, skeletal muscle (ACTA1), mRNA. | --- | Cluster0002 |
| --- | 14465664 | --- | --- | Cluster0002 |
| --- | 14467236 | PREDICTED: Canis lupus familiaris transmembrane protein 182 (TMEM182), mRNA. | --- | Cluster0002 |
| --- | 14467910 | PREDICTED: Canis lupus familiaris filamin C, gamma (FLNC), transcript variant X1, mRNA. | --- | Cluster0002 |
| --- | 14468132 | PREDICTED: Canis lupus familiaris solute carrier family 25 (mitochondrial carrier; phosphate carrier), member 3 (SLC25A3), transcript variant X1, mRNA. | --- | Cluster0002 |

| | | | | |
|---|---|---|---|---|
| --- | 14468504 | PREDICTED: Canis lupus familiaris catenin (cadherin-associated protein), alpha 2 (CTNNA2), mRNA. | --- | Cluster0002 |
| --- | 14468798 | PREDICTED: Canis lupus familiaris phosphorylase, glycogen, muscle (PYGM), mRNA. | --- | Cluster0002 |
| --- | 14469488 | PREDICTED: Canis lupus familiaris xin actin binding repeat containing 1 (XIRP1), transcript variant X1, mRNA. | --- | Cluster0002 |
| --- | 14469594 | PREDICTED: Canis lupus familiaris isocitrate dehydrogenase 3 (NAD+) beta (IDH3B), transcript variant X1, mRNA. | --- | Cluster0002 |
| --- | 14469596 | PREDICTED: Canis lupus familiaris isocitrate dehydrogenase 3 (NAD+) beta (IDH3B), transcript variant X1, mRNA. | --- | Cluster0002 |
| --- | 14469676 | PREDICTED: Canis lupus familiaris eukaryotic translation elongation factor 1 alpha 2 (EEF1A2), mRNA. | --- | Cluster0002 |
| --- | 14471058 | PREDICTED: Canis lupus familiaris titin (TTN), mRNA. | --- | Cluster0002 |
| --- | 14472784 | Canis lupus familiaris dystrophin (DMD), mRNA. | --- | Cluster0002 |
| --- | 14472786 | Canis lupus familiaris dystrophin (DMD), mRNA. | --- | Cluster0002 |
| --- | 14472788 | Canis lupus familiaris dystrophin (DMD), mRNA. | --- | Cluster0002 |
| --- | 14473090 | Canis lupus familiaris dystrophin (DMD), mRNA. | --- | Cluster0002 |
| --- | 14473092 | Canis lupus familiaris dystrophin (DMD), mRNA. | --- | Cluster0002 |
| --- | 14473250 | PREDICTED: Canis lupus familiaris FERM domain containing 3 (FRMD3), transcript variant X1, mRNA. | --- | Cluster0002 |
| --- | 14473252 | PREDICTED: Canis lupus familiaris FERM domain containing 3 (FRMD3), transcript variant X1, mRNA. | --- | Cluster0002 |
| --- | 14473278 | Canis lupus familiaris dystrophin (DMD), mRNA. | --- | Cluster0002 |
| --- | 14473656 | Canis lupus familiaris dystrophin (DMD), mRNA. | --- | Cluster0002 |
| --- | 14473658 | Canis lupus familiaris dystrophin (DMD), mRNA. | --- | Cluster0002 |
| --- | 14473660 | Canis lupus familiaris dystrophin (DMD), mRNA. | --- | Cluster0002 |

| | | | | |
|---|---|---|---|---|
| --- | 14474402 | PREDICTED: Canis lupus familiaris FERM domain containing 3 (FRMD3), transcript variant X1, mRNA. | --- | Cluster0002 |
| --- | 14474404 | PREDICTED: Canis lupus familiaris FERM domain containing 3 (FRMD3), transcript variant X1, mRNA. | --- | Cluster0002 |
| --- | 14474482 | PREDICTED: Canis lupus familiaris FERM domain containing 3 (FRMD3), transcript variant X1, mRNA. | --- | Cluster0002 |
| --- | 14474484 | PREDICTED: Canis lupus familiaris FERM domain containing 3 (FRMD3), transcript variant X1, mRNA. | --- | Cluster0002 |
| ABCC9 | 14363897 | PREDICTED: Canis lupus familiaris ATP-binding cassette, sub-family C (CFTR/MRP), member 9 (ABCC9), transcript variant X4, mRNA. | --- | Cluster0002 |
| ABRA | 14287760 | PREDICTED: Canis lupus familiaris actin binding Rho activating protein (ABRA), mRNA. | protein import into nucleus, translocation // positive regulation of Rho protein signal transduction // positive regulation of transcription, DNA-templated // positive regulation of transcription from RNA polymerase II promoter // positive regulation of sequence-specific DNA binding transcription factor activity | Cluster0002 |
| ACACB | 14361191 | PREDICTED: Canis lupus familiaris acetyl-CoA carboxylase beta (ACACB), transcript variant X1, mRNA. | acetyl-CoA metabolic process // fatty acid biosynthetic process // positive regulation of lipid storage // negative regulation of fatty acid oxidation // protein homotetramerization // positive regulation of heart growth | Cluster0002 |
| ACO2 | 14271370 | PREDICTED: Canis lupus familiaris aconitase 2, mitochondrial (ACO2), mRNA. | tricarboxylic acid cycle // citrate metabolic process // metabolic process | Cluster0002 |
| ACSM5 | 14426465 | PREDICTED: Canis lupus familiaris acyl-CoA synthetase medium-chain family member 5 (ACSM5), transcript variant X1, mRNA. | metabolic process | Cluster0002 |
| ACTA1 | 14402906 | PREDICTED: Canis lupus familiaris actin, alpha 1, skeletal muscle (ACTA1), mRNA. | --- | Cluster0002 |
| ACTC1 | 14381724 | PREDICTED: Canis lupus familiaris actin, alpha, cardiac muscle 1 (ACTC1), mRNA. | metabolic process // positive regulation of gene expression // actin filament-based movement // skeletal muscle thin filament assembly // actomyosin structure organization // actin-myosin filament sliding // negative regulation of apoptotic process // cardiac myofibril assembly // cardiac muscle tissue | Cluster0002 |

| | | | | |
|---|---|---|---|---|
| | | | morphogenesis // heart contraction // actin-mediated cell contraction // mesenchyme migration | |
| ACTN2 | 14405742 | PREDICTED: Canis lupus familiaris actinin, alpha 2 (ACTN2), transcript variant X2, mRNA. | --- | Cluster0002 |
| ADCK3 | 14434816 | PREDICTED: Canis lupus familiaris aarF domain containing kinase 3 (ADCK3), mRNA. | --- | Cluster0002 |
| ADGRL3 | 14287178 | PREDICTED: Canis lupus familiaris adhesion G protein-coupled receptor L3 (ADGRL3), transcript variant X4, mRNA. | --- | Cluster0002 |
| ADGRL3 | 14287184 | PREDICTED: Canis lupus familiaris adhesion G protein-coupled receptor L3 (ADGRL3), transcript variant X4, mRNA. | --- | Cluster0002 |
| ADPRHL1 | 14343934 | PREDICTED: Canis lupus familiaris ADP-ribosylhydrolase like 1 (ADPRHL1), transcript variant X1, mRNA. | --- | Cluster0002 |
| ALPK2 | 14255301 | PREDICTED: Canis lupus familiaris alpha-kinase 2 (ALPK2), transcript variant X6, mRNA. | --- | Cluster0002 |
| ANKRD1 | 14369962 | PREDICTED: Canis lupus familiaris ankyrin repeat domain 1 (cardiac muscle) (ANKRD1), mRNA. | regulation of transcription from RNA polymerase II promoter // cellular response to drug // skeletal muscle cell differentiation // response to muscle stretch // positive regulation of apoptotic process // positive regulation of DNA damage response, signal transduction by p53 class mediator // positive regulation of transcription from RNA polymerase II promoter // positive regulation of protein secretion // cardiac muscle tissue morphogenesis // cellular response to lipopolysaccharide // cellular response to mechanical stimulus // cellular response to interleukin-1 // cellular response to tumor necrosis factor // cellular response to transforming growth factor beta stimulus // negative regulation of nucleic acid-templated transcription // negative regulation of DNA biosynthetic process | Cluster0002 |
| APOBEC2 | 14280347 | PREDICTED: Canis lupus familiaris apolipoprotein B mRNA editing enzyme, catalytic polypeptide-like 2 (APOBEC2), mRNA. | mRNA modification // DNA demethylation | Cluster0002 |
| ART3 | 14386491 | PREDICTED: Canis lupus familiaris ADP-ribosyltransferase 3 (ART3), transcript variant X1, mRNA. | --- | Cluster0002 |

| | | | | |
|---|---|---|---|---|
| ASB12 | 14462588 | PREDICTED: Canis lupus familiaris ankyrin repeat and SOCS box containing 12 (ASB12), transcript variant X1, mRNA. | --- | Cluster0002 |
| ASB2 | 14442685 | PREDICTED: Canis lupus familiaris ankyrin repeat and SOCS box containing 2 (ASB2), transcript variant X2, mRNA. | --- | Cluster0002 |
| ASB5 | 14301073 | PREDICTED: Canis lupus familiaris ankyrin repeat and SOCS box containing 5 (ASB5), mRNA. | --- | Cluster0002 |
| ATP1A3 | 14259800 | PREDICTED: Canis lupus familiaris ATPase, Na+/K+ transporting, alpha 3 polypeptide (ATP1A3), transcript variant X2, mRNA. | --- | Cluster0002 |
| ATP2A2 | 14357744 | Canis lupus familiaris ATPase, Ca++ transporting, cardiac muscle, slow twitch 2 (ATP2A2), mRNA. | regulation of the force of heart contraction // cellular calcium ion homeostasis // ER-nucleus signaling pathway // organelle organization // metabolic process // regulation of cardiac muscle contraction by calcium ion signaling // transition between fast and slow fiber // positive regulation of endoplasmic reticulum calcium ion concentration // T-tubule organization // cellular response to oxidative stress // negative regulation of heart contraction // relaxation of cardiac muscle // sarcoplasmic reticulum calcium ion transport // calcium ion transmembrane transport // regulation of cardiac muscle cell membrane potential // regulation of calcium ion-dependent exocytosis of neurotransmitter // calcium ion transport from cytosol to endoplasmic reticulum // transport // ion transport // calcium ion transport // cellular calcium ion homeostasis // cellular calcium ion homeostasis // regulation of cardiac muscle contraction by calcium ion signaling // regulation of cardiac muscle contraction by calcium ion signaling // endoplasmic reticulum calcium ion homeostasis // calcium ion transmembrane transport // calcium ion transmembrane transport // regulation of cardiac muscle cell membrane potential | Cluster0002 |
| ATP5B | 14270159 | PREDICTED: Canis lupus familiaris ATP synthase, H+ transporting, mitochondrial F1 complex, beta polypeptide (ATP5B), mRNA. | proton transport // ATP metabolic process // ATP biosynthetic process // transport // ion transport // ATP synthesis coupled proton transport // ATP hydrolysis coupled proton transport | Cluster0002 |

| | | | | |
|---|---|---|---|---|
| B3GALT2 | 14400701 | PREDICTED: Canis lupus familiaris UDP-Gal:betaGlcNAc beta 1,3-galactosyltransferase, polypeptide 2 (B3GALT2), mRNA. | protein glycosylation // oligosaccharide biosynthetic process | Cluster0002 |
| BCHE | 14393431 | Canis lupus familiaris butyrylcholinesterase (BCHE), mRNA. | metabolic process | Cluster0002 |
| C28H10orf71 | 14367549 | PREDICTED: Canis lupus familiaris chromosome 28 open reading frame, human C10orf71 (C28H10orf71), transcript variant X1, mRNA. | --- | Cluster0002 |
| C28H10orf71 | 14367551 | PREDICTED: Canis lupus familiaris chromosome 28 open reading frame, human C10orf71 (C28H10orf71), transcript variant X1, mRNA. | --- | Cluster0002 |
| C8H14orf180 | 14439967 | PREDICTED: Canis lupus familiaris chromosome 8 open reading frame, human C14orf180 (C8H14orf180), transcript variant X2, mRNA. | --- | Cluster0002 |
| CA14 | 14306085 | PREDICTED: Canis lupus familiaris carbonic anhydrase XIV (CA14), transcript variant X1, mRNA. | --- | Cluster0002 |
| CACNA1H | 14427535 | PREDICTED: Canis lupus familiaris calcium channel, voltage-dependent, T type, alpha 1H subunit (CACNA1H), transcript variant X1, mRNA. | transport // ion transport // ion transmembrane transport // regulation of ion transmembrane transport // transmembrane transport // calcium ion import // calcium ion transmembrane transport | Cluster0002 |
| CASQ2 | 14308968 | Canis lupus familiaris calsequestrin 2 (cardiac muscle) (CASQ2), mRNA. | regulation of release of sequestered calcium ion into cytosol by sarcoplasmic reticulum // negative regulation of ryanodine-sensitive calcium-release channel activity | Cluster0002 |
| CASQ2 | 14308970 | Canis lupus familiaris calsequestrin 2 (cardiac muscle) (CASQ2), mRNA. | regulation of release of sequestered calcium ion into cytosol by sarcoplasmic reticulum // negative regulation of ryanodine-sensitive calcium-release channel activity | Cluster0002 |
| CAV3 | 14333316 | Canis lupus familiaris caveolin 3 (CAV3), mRNA. | regulation of signaling // muscle cell cellular homeostasis // caveola assembly // plasma membrane repair // regulation of heart rate // negative regulation of protein kinase activity // triglyceride metabolic process // plasma membrane organization // actin filament organization // myoblast fusion // protein localization // negative regulation of cardiac muscle hypertrophy // positive regulation of myotube differentiation // regulation of skeletal muscle contraction // myotube differentiation // regulation of transforming growth factor beta receptor signaling pathway // cytoplasmic microtubule organization // membrane raft organization // detection of | Cluster0002 |

| | | | | |
|---|---|---|---|---|
| | | | muscle stretch // regulation of signal transduction by receptor internalization // regulation of membrane potential // glucose homeostasis // cholesterol homeostasis // negative regulation of MAP kinase activity // negative regulation of MAPK cascade // negative regulation of cell size // negative regulation of nitric-oxide synthase activity // regulation of nerve growth factor receptor activity // nucleus localization // regulation of protein kinase B signaling // regulation of calcium ion transport // negative regulation of calcium ion transport // cardiac muscle cell development // regulation of cardiac muscle contraction // negative regulation of sarcomere organization // regulation of ventricular cardiac muscle cell membrane repolarization // heart trabecula formation // regulation of ventricular cardiac muscle cell membrane depolarization // regulation of branching involved in mammary gland duct morphogenesis // protein localization to plasma membrane // establishment of protein localization to plasma membrane // regulation of calcium ion import // regulation of p38MAPK cascade // regulation of membrane depolarization during cardiac muscle cell action potential // negative regulation of membrane depolarization during cardiac muscle cell action potential // regulation of calcium ion transmembrane transporter activity // regulation of sodium ion transmembrane transporter activity | |
| CCDC85A | 14269516 | PREDICTED: Canis lupus familiaris coiled-coil domain containing 85A (CCDC85A), mRNA. | --- | Cluster0002 |
| CDH7 | 14260856 | PREDICTED: Canis lupus familiaris cadherin 7, type 2 (CDH7), mRNA. | --- | Cluster0002 |
| CFL2 | 14440902 | PREDICTED: Canis lupus familiaris cofilin 2 (muscle) (CFL2), mRNA. | actin filament organization // actin filament depolymerization // positive regulation of actin filament depolymerization // sarcomere organization // muscle cell cellular homeostasis | Cluster0002 |
| CHRM2 | 14301705 | PREDICTED: Canis lupus familiaris cholinergic receptor, muscarinic 2 (CHRM2), transcript variant X1, mRNA. | G-protein coupled receptor signaling pathway // G-protein coupled acetylcholine receptor signaling pathway // regulation of heart contraction // regulation of smooth muscle contraction // signal transduction // adenylate cyclase-inhibiting G-protein coupled acetylcholine receptor signaling pathway // phospholipase C-activating G-protein coupled acetylcholine receptor signaling pathway // synaptic transmission, cholinergic // sensory perception of chemical stimulus // regulation of heart | Cluster0002 |

| | | | | |
|---|---|---|---|---|
| | | | contraction // response to virus // positive regulation of adenylate cyclase activity involved in G-protein coupled receptor signaling pathway | |
| CKM | 14259494 | Canis lupus familiaris creatine kinase, muscle (CKM), mRNA. | response to heat // phosphorylation | Cluster0002 |
| CKMT2 | 14376771 | Canis lupus familiaris creatine kinase, mitochondrial 2 (sarcomeric) (CKMT2), mRNA. | phosphorylation | Cluster0002 |
| CMYA5 | 14376879 | PREDICTED: Canis lupus familiaris cardiomyopathy associated 5 (CMYA5), mRNA. | --- | Cluster0002 |
| CORIN | 14289187 | PREDICTED: Canis lupus familiaris corin, serine peptidase (CORIN), mRNA. | --- | Cluster0002 |
| COX6A2 | 14420721 | PREDICTED: Canis lupus familiaris cytochrome c oxidase subunit VIa polypeptide 2 (COX6A2), mRNA. | hydrogen ion transmembrane transport | Cluster0002 |
| CPT1B | 14267458 | PREDICTED: Canis lupus familiaris carnitine palmitoyltransferase 1B (muscle) (CPT1B), transcript variant X1, mRNA. | --- | Cluster0002 |
| CRHR2 | 14293240 | PREDICTED: Canis lupus familiaris corticotropin releasing hormone receptor 2 (CRHR2), transcript variant X1, mRNA. | signal transduction // cell surface receptor signaling pathway // G-protein coupled receptor signaling pathway // negative regulation of angiogenesis // positive regulation of cAMP-mediated signaling // negative regulation of cAMP-mediated signaling // cellular response to corticotropin-releasing hormone stimulus | Cluster0002 |
| CSRP3 | 14341457 | PREDICTED: Canis lupus familiaris cysteine and glycine-rich protein 3 (cardiac LIM protein) (CSRP3), mRNA. | regulation of the force of heart contraction // cardiac muscle hypertrophy // cellular calcium ion homeostasis // protein localization to organelle // detection of muscle stretch // positive regulation of transcription from RNA polymerase II promoter // cardiac muscle tissue development // cardiac myofibril assembly // cardiac muscle contraction | Cluster0002 |
| CTNNA2 | 14308480 | PREDICTED: Canis lupus familiaris catenin (cadherin-associated protein), alpha 2 (CTNNA2), mRNA. | --- | Cluster0002 |
| CTNNA3 | 14406173 | PREDICTED: Canis lupus familiaris catenin (cadherin-associated protein), alpha 3 (CTNNA3), transcript variant X1, mRNA. | --- | Cluster0002 |
| CXADR | 14384493 | PREDICTED: Canis lupus familiaris coxsackie virus and adenovirus receptor (CXADR), transcript variant X1, mRNA. | --- | Cluster0002 |

| | | | | |
|---|---|---|---|---|
| DES | 14398871 | Canis lupus familiaris desmin (DES), mRNA. | --- | Cluster0002 |
| DMD | 14461657 | Canis lupus familiaris dystrophin (DMD), mRNA. | muscle attachment | Cluster0002 |
| DSC2 | 14432404 | PREDICTED: Canis lupus familiaris desmocollin 2 (DSC2), transcript variant X2, mRNA. | cell adhesion // homophilic cell adhesion via plasma membrane adhesion molecules | Cluster0002 |
| DSP | 14393883 | PREDICTED: Canis lupus familiaris desmoplakin (DSP), mRNA. | --- | Cluster0002 |
| DTNA | 14435723 | PREDICTED: Canis lupus familiaris dystrobrevin, alpha (DTNA), transcript variant X25, mRNA. | --- | Cluster0002 |
| DUSP27 | 14434625 | PREDICTED: Canis lupus familiaris dual specificity phosphatase 27 (putative) (DUSP27), transcript variant X2, mRNA. | --- | Cluster0002 |
| DYSF | 14308693 | PREDICTED: Canis lupus familiaris dysferlin (DYSF), transcript variant X6, mRNA. | --- | Cluster0002 |
| ENO3 | 14415584 | PREDICTED: Canis lupus familiaris enolase 3 (beta, muscle) (ENO3), transcript variant X2, mRNA. | glycolytic process | Cluster0002 |
| ESRRG | 14401810 | PREDICTED: Canis lupus familiaris estrogen-related receptor gamma (ESRRG), transcript variant X2, mRNA. | --- | Cluster0002 |
| FABP3 | 14321832 | PREDICTED: Canis lupus familiaris fatty acid binding protein 3, muscle and heart (FABP3), mRNA. | transport | Cluster0002 |
| FBXO40 | 14389519 | PREDICTED: Canis lupus familiaris F-box protein 40 (FBXO40), mRNA. | protein ubiquitination | Cluster0002 |
| FHL2 | 14268768 | PREDICTED: Canis lupus familiaris four and a half LIM domains 2 (FHL2), transcript variant X2, mRNA. | negative regulation of transcription from RNA polymerase II promoter // osteoblast differentiation // regulation of transcription from RNA polymerase II promoter // response to hormone // negative regulation of apoptotic process // negative regulation of transcription, DNA-templated // atrial cardiac muscle cell development // ventricular cardiac muscle cell development // heart trabecula formation | Cluster0002 |
| FHOD3 | 14435631 | PREDICTED: Canis lupus familiaris formin homology 2 domain containing 3 (FHOD3), mRNA. | --- | Cluster0002 |
| FHOD3 | 14435651 | PREDICTED: Canis lupus familiaris formin homology 2 domain containing 3 (FHOD3), mRNA. | --- | Cluster0002 |

| | | | | |
|---|---|---|---|---|
| FITM1 | 14437001 | PREDICTED: Canis lupus familiaris fat storage-inducing transmembrane protein 1 (FITM1), mRNA. | phospholipid biosynthetic process // positive regulation of sequestering of triglyceride // lipid storage // lipid storage // lipid particle organization // lipid particle organization | Cluster0002 |
| FITM2 | 14351773 | PREDICTED: Canis lupus familiaris fat storage-inducing transmembrane protein 2 (FITM2), mRNA. | --- | Cluster0002 |
| FLNC | 14292196 | PREDICTED: Canis lupus familiaris filamin C, gamma (FLNC), transcript variant X1, mRNA. | muscle fiber development | Cluster0002 |
| FPGT | 14428957 | Canis lupus familiaris fucose-1-phosphate guanylyltransferase (FPGT), mRNA. | metabolic process | Cluster0002 |
| FRAS1 | 14386565 | PREDICTED: Canis lupus familiaris Fraser extracellular matrix complex subunit 1 (FRAS1), mRNA. | cell communication | Cluster0002 |
| FRAS1 | 14386568 | PREDICTED: Canis lupus familiaris Fraser extracellular matrix complex subunit 1 (FRAS1), mRNA. | cell communication | Cluster0002 |
| FSD2 | 14378062 | PREDICTED: Canis lupus familiaris fibronectin type III and SPRY domain containing 2 (FSD2), transcript variant X1, mRNA. | --- | Cluster0002 |
| FXR2 | 14415887 | PREDICTED: Canis lupus familiaris fragile X mental retardation, autosomal homolog 2 (FXR2), transcript variant X2, mRNA. | --- | Cluster0002 |
| GMPR | 14394074 | PREDICTED: Canis lupus familiaris guanosine monophosphate reductase (GMPR), mRNA. | nucleotide metabolic process // oxidation-reduction process | Cluster0002 |
| GNAO1 | 14325359 | PREDICTED: Canis lupus familiaris guanine nucleotide binding protein (G protein), alpha activating activity polypeptide O (GNAO1), transcript variant X1, mRNA. | signal transduction // G-protein coupled receptor signaling pathway // adenylate cyclase-modulating G-protein coupled receptor signaling pathway // adenylate cyclase-modulating G-protein coupled receptor signaling pathway // dopamine receptor signaling pathway // dopamine receptor signaling pathway // locomotory behavior // regulation of heart contraction // metabolic process // cellular process | Cluster0002 |
| GOT1 | 14370464 | Canis lupus familiaris glutamic-oxaloacetic transaminase 1, soluble (GOT1), mRNA. | oxaloacetate metabolic process // glycerol biosynthetic process // cellular amino acid metabolic process // aspartate biosynthetic process // aspartate catabolic process // Notch signaling pathway // biosynthetic process // glutamate catabolic process to aspartate // glutamate catabolic process to 2-oxoglutarate // cellular response to insulin stimulus // response to glucocorticoid // fatty acid homeostasis | Cluster0002 |

| | | | | |
|---|---|---|---|---|
| GPX3 | 14407751 | Canis lupus familiaris glutathione peroxidase 3 (GPX3), mRNA. | --- | Cluster0002 |
| HACD1 | 14319474 | Canis lupus familiaris protein tyrosine phosphatase-like (proline instead of catalytic arginine), member A (HACD1), transcript variant 1, mRNA. | sphingolipid biosynthetic process // fatty acid elongation // very long-chain fatty acid biosynthetic process // negative regulation of catalytic activity // lipid metabolic process // fatty acid metabolic process // fatty acid biosynthetic process | Cluster0002 |
| HAO2 | 14305852 | hydroxyacid oxidase 2 (long chain) [gene_biotype:protein_coding transcript_biotype:protein_coding] | oxidation-reduction process // glutamate biosynthetic process // fatty acid oxidation | Cluster0002 |
| HHATL | 14346363 | PREDICTED: Canis lupus familiaris hedgehog acyltransferase-like (HHATL), transcript variant X5, mRNA. | --- | Cluster0002 |
| HPGDS | 14388191 | Canis lupus familiaris hematopoietic prostaglandin D synthase (HPGDS), mRNA. | --- | Cluster0002 |
| HRC | 14258937 | PREDICTED: Canis lupus familiaris histidine rich calcium binding protein (HRC), mRNA. | --- | Cluster0002 |
| HSPB3 | 14408002 | PREDICTED: Canis lupus familiaris heat shock 27kDa protein 3 (HSPB3), mRNA. | --- | Cluster0002 |
| HSPB7 | 14322948 | PREDICTED: Canis lupus familiaris heat shock 27kDa protein family, member 7 (cardiovascular) (HSPB7), mRNA. | --- | Cluster0002 |
| IDH3A | 14375310 | PREDICTED: Canis lupus familiaris isocitrate dehydrogenase 3 (NAD+) alpha (IDH3A), transcript variant X3, mRNA. | --- | Cluster0002 |
| ITGB1BP2 | 14458986 | PREDICTED: Canis lupus familiaris integrin beta 1 binding protein (melusin) 2 (ITGB1BP2), transcript variant X2, mRNA. | --- | Cluster0002 |
| ITGB6 | 14396390 | PREDICTED: Canis lupus familiaris integrin, beta 6 (ITGB6), transcript variant X4, mRNA. | --- | Cluster0002 |
| KCNJ3 | 14395279 | PREDICTED: Canis lupus familiaris potassium channel, inwardly rectifying subfamily J, member 3 (KCNJ3), transcript variant X3, mRNA. | --- | Cluster0002 |
| KCNJ3 | 14395285 | PREDICTED: Canis lupus familiaris potassium channel, inwardly rectifying subfamily J, member 3 (KCNJ3), transcript variant X1, mRNA. | transport // ion transport // potassium ion transport // potassium ion import // regulation of ion transmembrane transport // regulation of ion transmembrane transport | Cluster0002 |
| KCNJ5 | 14413968 | PREDICTED: Canis lupus familiaris potassium channel, inwardly rectifying subfamily J, member 5 (KCNJ5), mRNA. | transport // ion transport // potassium ion transport // potassium ion import // regulation of ion transmembrane transport // regulation of ion transmembrane transport | Cluster0002 |

| | | | | |
|---|---|---|---|---|
| KLHL31 | 14284068 | PREDICTED: Canis lupus familiaris kelch-like family member 31 (KLHL31), transcript variant X2, mRNA. | regulation of transcription, DNA-templated // skeletal muscle tissue development // protein ubiquitination | Cluster0002 |
| KLHL41 | 14395543 | PREDICTED: Canis lupus familiaris kelch-like family member 41 (KLHL41), mRNA. | --- | Cluster0002 |
| LDB3 | 14403793 | PREDICTED: Canis lupus familiaris LIM domain binding 3 (LDB3), transcript variant X1, mRNA. | --- | Cluster0002 |
| LMO3 | 14364028 | PREDICTED: Canis lupus familiaris LIM domain only 3 (rhombotin-like 2) (LMO3), transcript variant X3, mRNA. | --- | Cluster0002 |
| LMOD2 | 14292333 | PREDICTED: Canis lupus familiaris leiomodin 2 (cardiac) (LMOD2), mRNA. | --- | Cluster0002 |
| LOC479934 | 14423291 | PREDICTED: Canis lupus familiaris lipid phosphate phosphatase-related protein type 5 (LOC479934), mRNA. | --- | Cluster0002 |
| LOC491446 | 14456630 | PREDICTED: Canis lupus familiaris myomesin-2 (LOC491446), partial mRNA. | --- | Cluster0002 |
| LOC608697 | 14344974 | PREDICTED: Canis lupus familiaris 2-hydroxyacyl-CoA lyase 1 (LOC608697), transcript variant X1, mRNA. | --- | Cluster0002 |
| LRRC10 | 14270645 | PREDICTED: Canis lupus familiaris leucine rich repeat containing 10 (LRRC10), mRNA. | cardiac muscle cell development | Cluster0002 |
| LRRC2 | 14329589 | PREDICTED: Canis lupus familiaris leucine rich repeat containing 2 (LRRC2), transcript variant X2, mRNA. | --- | Cluster0002 |
| MBOAT2 | 14306657 | PREDICTED: Canis lupus familiaris membrane bound O-acyltransferase domain containing 2 (MBOAT2), mRNA. | --- | Cluster0002 |
| MDH2 | 14419785 | PREDICTED: Canis lupus familiaris malate dehydrogenase 2, NAD (mitochondrial) (MDH2), mRNA. | carbohydrate metabolic process // tricarboxylic acid cycle // malate metabolic process // carboxylic acid metabolic process // oxidation-reduction process | Cluster0002 |
| MLIP | 14281076 | PREDICTED: Canis lupus familiaris muscular LMNA-interacting protein (MLIP), transcript variant X1, mRNA. | negative regulation of transcription from RNA polymerase II promoter // negative regulation of cardiac muscle hypertrophy | Cluster0002 |
| MTUS2 | 14355280 | PREDICTED: Canis lupus familiaris microtubule associated tumor suppressor candidate 2 (MTUS2), transcript variant X7, mRNA. | --- | Cluster0002 |

| | | | | |
|---|---|---|---|---|
| MURC | 14275836 | PREDICTED: Canis lupus familiaris muscle-related coiled-coil protein (MURC), mRNA. | regulation of gene expression // positive regulation of transcription from RNA polymerase II promoter | Cluster0002 |
| MYBPC3 | 14311489 | Canis lupus familiaris myosin binding protein C, cardiac (MYBPC3), mRNA. | --- | Cluster0002 |
| MYH7 | 14440427 | Canis lupus familiaris myosin, heavy chain 7, cardiac muscle, beta (MYH7), mRNA. | metabolic process | Cluster0002 |
| MYL4 | 14450312 | PREDICTED: Canis lupus familiaris myosin, light chain 4, alkali; atrial, embryonic (MYL4), mRNA. | --- | Cluster0002 |
| MYLK3 | 14294279 | PREDICTED: Canis lupus familiaris myosin light chain kinase 3 (MYLK3), mRNA. | regulation of vascular permeability involved in acute inflammatory response // protein phosphorylation // phosphorylation // sarcomere organization // cellular response to interleukin-1 | Cluster0002 |
| MYO18B | 14358506 | PREDICTED: Canis lupus familiaris myosin XVIIIB (MYO18B), transcript variant X1, mRNA. | --- | Cluster0002 |
| MYOM2 | 14301122 | PREDICTED: Canis lupus familiaris myomesin 2 (MYOM2), mRNA. | --- | Cluster0002 |
| MYOM3 | 14322225 | PREDICTED: Canis lupus familiaris myomesin 3 (MYOM3), mRNA. | --- | Cluster0002 |
| MYOZ1 | 14406587 | Canis lupus familiaris myozenin 1 (MYOZ1), mRNA. | --- | Cluster0002 |
| MYPN | 14403050 | PREDICTED: Canis lupus familiaris myopalladin (MYPN), transcript variant X1, mRNA. | --- | Cluster0002 |
| MYZAP | 14380649 | PREDICTED: Canis lupus familiaris myocardial zonula adherens protein (MYZAP), transcript variant X1, mRNA. | intracellular signal transduction // intracellular signal transduction | Cluster0002 |
| NEBL | 14319308 | PREDICTED: Canis lupus familiaris nebulette (NEBL), transcript variant X2, mRNA. | --- | Cluster0002 |
| NPPA | 14323010 | Canis lupus familiaris natriuretic peptide A (NPPA), mRNA. | cGMP biosynthetic process // receptor guanylyl cyclase signaling pathway // neuropeptide signaling pathway // female pregnancy // regulation of blood pressure // positive regulation of heart rate // cardiac muscle hypertrophy in response to stress // regulation of blood vessel size // regulation of atrial cardiac muscle cell membrane repolarization // positive regulation of cardiac muscle contraction // positive regulation of delayed rectifier potassium channel activity // positive regulation of potassium ion export across plasma membrane | Cluster0002 |
| NPR3 | 14408494 | PREDICTED: Canis lupus familiaris natriuretic peptide receptor 3 (NPR3), mRNA. | --- | Cluster0002 |

| | | | | |
|---|---|---|---|---|
| NRAP | 14371098 | PREDICTED: Canis lupus familiaris nebulin-related anchoring protein (NRAP), transcript variant X1, mRNA. | --- | Cluster0002 |
| NT5C1A | 14293865 | PREDICTED: Canis lupus familiaris 5-nucleotidase, cytosolic IA (NT5C1A), mRNA. | nucleotide metabolic process // purine nucleoside monophosphate catabolic process // dephosphorylation | Cluster0002 |
| PALLD | 14353398 | PREDICTED: Canis lupus familiaris palladin, cytoskeletal associated protein (PALLD), transcript variant X2, mRNA. | --- | Cluster0002 |
| PCLO | 14313855 | PREDICTED: Canis lupus familiaris piccolo presynaptic cytomatrix protein (PCLO), mRNA. | --- | Cluster0002 |
| PDHA1 | 14457570 | PREDICTED: Canis lupus familiaris pyruvate dehydrogenase (lipoamide) alpha 1 (PDHA1), mRNA. | acetyl-CoA biosynthetic process from pyruvate // tricarboxylic acid cycle // metabolic process // oxidation-reduction process | Cluster0002 |
| PEBP4 | 14353830 | PREDICTED: Canis lupus familiaris phosphatidylethanolamine-binding protein 4 (PEBP4), transcript variant X2, mRNA. | --- | Cluster0002 |
| PGAM2 | 14455723 | PREDICTED: Canis lupus familiaris phosphoglycerate mutase 2 (muscle) (PGAM2), mRNA. | --- | Cluster0002 |
| PLA2G4E | 14382055 | PREDICTED: Canis lupus familiaris phospholipase A2, group IVE (PLA2G4E), transcript variant X1, mRNA. | --- | Cluster0002 |
| PLA2G5 | 14326594 | PREDICTED: Canis lupus familiaris phospholipase A2, group V (PLA2G5), transcript variant X5, mRNA. | lipid metabolic process // phospholipid metabolic process // positive regulation of phospholipase activity // lipid catabolic process // positive regulation of ERK1 and ERK2 cascade | Cluster0002 |
| PLN | 14256644 | Canis lupus familiaris phospholamban (PLN), mRNA. | calcium ion transport // cellular calcium ion homeostasis // Notch signaling pathway // negative regulation of heart rate // regulation of cardiac muscle contraction by regulation of the release of sequestered calcium ion // negative regulation of ATPase activity // cardiac muscle tissue development // regulation of calcium ion transport // regulation of ryanodine-sensitive calcium-release channel activity // regulation of cardiac muscle cell contraction // adrenergic receptor signaling pathway involved in heart process // regulation of the force of heart contraction by cardiac conduction // negative regulation of calcium ion transmembrane transporter activity // regulation of relaxation of muscle // negative regulation of calcium-transporting ATPase activity // negative regulation of calcium | Cluster0002 |

| | | | | |
|---|---|---|---|---|
| | | | ion import into sarcoplasmic reticulum // regulation of the force of heart contraction // regulation of heart contraction // regulation of release of sequestered calcium ion into cytosol by sarcoplasmic reticulum // negative regulation of heart contraction // negative regulation of calcium ion transport // regulation of calcium ion import // regulation of calcium-transporting ATPase activity | |
| PPARGC1A | 14376136 | PREDICTED: Canis lupus familiaris peroxisome proliferator-activated receptor gamma, coactivator 1 alpha (PPARGC1A), transcript variant X1, mRNA. | --- | Cluster0002 |
| PPP1R12B | 14433201 | PREDICTED: Canis lupus familiaris protein phosphatase 1, regulatory subunit 12B (PPP1R12B), transcript variant X10, mRNA. | --- | Cluster0002 |
| PPP1R3A | 14293498 | PREDICTED: Canis lupus familiaris protein phosphatase 1, regulatory subunit 3A (PPP1R3A), transcript variant X3, mRNA. | --- | Cluster0002 |
| PRKAA2 | 14417135 | PREDICTED: Canis lupus familiaris protein kinase, AMP-activated, alpha 2 catalytic subunit (PRKAA2), mRNA. | --- | Cluster0002 |
| PRR32 | 14460092 | PREDICTED: Canis lupus familiaris proline rich 32 (PRR32), mRNA. | --- | Cluster0002 |
| PTP4A3 | 14286396 | PREDICTED: Canis lupus familiaris protein tyrosine phosphatase type IVA, member 3 (PTP4A3), transcript variant X5, mRNA. | --- | Cluster0002 |
| PYGM | 14312707 | PREDICTED: Canis lupus familiaris phosphorylase, glycogen, muscle (PYGM), mRNA. | carbohydrate metabolic process // glycogen catabolic process // glycogen catabolic process | Cluster0002 |
| RBM20 | 14368775 | PREDICTED: Canis lupus familiaris RNA binding motif protein 20 (RBM20), transcript variant X5, mRNA. | --- | Cluster0002 |
| RBPMS2 | 14383583 | PREDICTED: Canis lupus familiaris RNA binding protein with multiple splicing 2 (RBPMS2), transcript variant X2, mRNA. | --- | Cluster0002 |
| RGS6 | 14438528 | PREDICTED: Canis lupus familiaris regulator of G-protein signaling 6 (RGS6), transcript variant X1, mRNA. | --- | Cluster0002 |

| | | | | |
|---|---|---|---|---|
| RGS7BP | 14320679 | PREDICTED: Canis lupus familiaris regulator of G-protein signaling 7 binding protein (RGS7BP), transcript variant X3, mRNA. | --- | Cluster0002 |
| RPL3L | 14422516 | PREDICTED: Canis lupus familiaris ribosomal protein L3-like (RPL3L), mRNA. | --- | Cluster0002 |
| RYR2 | 14405620 | PREDICTED: Canis lupus familiaris ryanodine receptor 2 (cardiac) (RYR2), transcript variant X7, mRNA. | calcium ion transport // calcium ion transmembrane transport | Cluster0002 |
| SCNN1A | 14364723 | PREDICTED: Canis lupus familiaris sodium channel, non voltage gated 1 alpha subunit (SCNN1A), transcript variant X1, mRNA. | transport // ion transport // sodium ion transport // sodium ion transmembrane transport | Cluster0002 |
| SDHA | 14392782 | PREDICTED: Canis lupus familiaris succinate dehydrogenase complex, subunit A, flavoprotein (Fp) (SDHA), transcript variant X1, mRNA. | --- | Cluster0002 |
| SGCA | 14446295 | PREDICTED: Canis lupus familiaris sarcoglycan, alpha (50kDa dystrophin-associated glycoprotein) (SGCA), transcript variant X1, mRNA. | --- | Cluster0002 |
| SLC22A2 | 14262029 | Canis lupus familiaris solute carrier family 22 (organic cation transporter), member 2 (SLC22A2), mRNA. | transmembrane transport // cation transport // organic cation transport // organic cation transport // quaternary ammonium group transport // choline transport // dopamine transport // ammonium transmembrane transport | Cluster0002 |
| SLC22A3 | 14256364 | PREDICTED: Canis lupus familiaris solute carrier family 22 (organic cation transporter), member 3 (SLC22A3), transcript variant X2, mRNA. | --- | Cluster0002 |
| SLC25A4 | 14303122 | PREDICTED: Canis lupus familiaris solute carrier family 25 (mitochondrial carrier; adenine nucleotide translocator), member 4 (SLC25A4), mRNA. | --- | Cluster0002 |
| SMPX | 14461553 | PREDICTED: Canis lupus familiaris small muscle protein, X-linked (SMPX), mRNA. | --- | Cluster0002 |
| SMYD1 | 14308230 | PREDICTED: Canis lupus familiaris SET and MYND domain containing 1 (SMYD1), transcript variant X2, mRNA. | --- | Cluster0002 |
| SRL | 14422144 | PREDICTED: Canis lupus familiaris sarcalumenin (SRL), transcript variant X2, mRNA. | --- | Cluster0002 |
| SYNPO2L | 14406593 | PREDICTED: Canis lupus familiaris synaptopodin 2-like (SYNPO2L), transcript variant X1, mRNA. | --- | Cluster0002 |

| | | | | |
|---|---|---|---|---|
| TBC1D8 | 14268832 | PREDICTED: Canis lupus familiaris TBC1 domain family, member 8 (with GRAM domain) (TBC1D8), transcript variant X1, mRNA. | --- | Cluster0002 |
| TCAP | 14451593 | Canis lupus familiaris titin-cap (TCAP), mRNA. | somitogenesis // skeletal muscle contraction // cardiac muscle hypertrophy // adult heart development // cardiac muscle hypertrophy in response to stress // skeletal muscle thin filament assembly // skeletal muscle myosin thick filament assembly // otic vesicle formation // detection of muscle stretch // cardiac muscle fiber development // sarcomerogenesis // cardiac myofibril assembly // cardiac muscle tissue morphogenesis // cardiac muscle contraction | Cluster0002 |
| TECRL | 14289516 | PREDICTED: Canis lupus familiaris trans-2,3-enoyl-CoA reductase-like (TECRL), transcript variant X2, mRNA. | --- | Cluster0002 |
| THAP4 | 14357093 | PREDICTED: Canis lupus familiaris THAP domain containing 4 (THAP4), mRNA. | --- | Cluster0002 |
| TMEM163 | 14318615 | PREDICTED: Canis lupus familiaris transmembrane protein 163 (TMEM163), mRNA. | --- | Cluster0002 |
| TNNC1 | 14328564 | PREDICTED: Canis lupus familiaris troponin C type 1 (slow) (TNNC1), mRNA. | regulation of muscle contraction // transition between fast and slow fiber // regulation of muscle filament sliding speed // regulation of ATPase activity // ventricular cardiac muscle tissue morphogenesis // cardiac muscle contraction | Cluster0002 |
| TNNI3 | 14258257 | Canis lupus familiaris troponin I type 3 (cardiac) (TNNI3), mRNA. | vasculogenesis // regulation of systemic arterial blood pressure by ischemic conditions // skeletal muscle contraction // cellular calcium ion homeostasis // regulation of muscle contraction // negative regulation of ATPase activity // ventricular cardiac muscle tissue morphogenesis // cardiac muscle contraction // skeletal muscle contraction // striated muscle contraction // heart development // heart contraction // cardiac muscle contraction // cardiac muscle contraction | Cluster0002 |
| TRDN | 14262448 | Canis lupus familiaris triadin (TRDN), mRNA. | regulation of release of sequestered calcium ion into cytosol by sarcoplasmic reticulum // positive regulation of ryanodine-sensitive calcium-release channel activity // regulation of cardiac muscle cell membrane potential // positive regulation of cell communication by electrical coupling involved in cardiac conduction | Cluster0002 |
| TSHB | 14305716 | Canis lupus familiaris thyroid stimulating hormone, beta (TSHB), mRNA. | --- | Cluster0002 |

| | | | | |
|---|---|---|---|---|
| TTN | 14397065 | PREDICTED: Canis lupus familiaris titin (TTN), mRNA. | --- | Cluster0002 |
| TXLNB | 14261539 | PREDICTED: Canis lupus familiaris taxilin beta (TXLNB), mRNA. | --- | Cluster0002 |
| UNC45B | 14452655 | PREDICTED: Canis lupus familiaris unc-45 myosin chaperone B (UNC45B), transcript variant X1, mRNA. | chaperone-mediated protein folding | Cluster0002 |
| USP13 | 14391511 | PREDICTED: Canis lupus familiaris ubiquitin specific peptidase 13 (isopeptidase T-3) (USP13), mRNA. | regulation of transcription, DNA-templated // proteolysis // ubiquitin-dependent protein catabolic process // cell proliferation // regulation of autophagy // regulation of autophagy // protein deubiquitination // protein deubiquitination // proteasome-mediated ubiquitin-dependent protein catabolic process // protein stabilization // regulation of proteasomal protein catabolic process // protein K63-linked deubiquitination // positive regulation of ERAD pathway // maintenance of unfolded protein involved in ERAD pathway | Cluster0002 |
| XIRP2 | 14395456 | PREDICTED: Canis lupus familiaris xin actin binding repeat containing 2 (XIRP2), transcript variant X1, mRNA. | heart development // actin cytoskeleton organization | Cluster0002 |
| ZNF106 | 14382162 | PREDICTED: Canis lupus familiaris zinc finger protein 106 (ZNF106), transcript variant X1, mRNA. | --- | Cluster0002 |
| | | | | |
| **Low in all MMVD** | | | | |
| **Name** | | **mRNA - Description** | **GO Biological Process Term** | **MCL_1.7_6** |
| --- | 14345868 | --- | --- | Cluster0023 |
| --- | 14465586 | PREDICTED: Canis lupus familiaris neuronal regeneration related protein (NREP), mRNA. | --- | Cluster0023 |
| ADCY2 | 14392600 | PREDICTED: Canis lupus familiaris adenylate cyclase 2 (brain) (ADCY2), mRNA. | --- | Cluster0023 |
| ADCY2 | 14392602 | PREDICTED: Canis lupus familiaris adenylate cyclase 2 (brain) (ADCY2), mRNA. | --- | Cluster0023 |
| FSTL4 | 14276971 | PREDICTED: Canis lupus familiaris follistatin-like 4 (FSTL4), transcript variant X2, mRNA. | --- | Cluster0023 |
| FSTL4 | 14276984 | PREDICTED: Canis lupus familiaris follistatin-like 4 (FSTL4), transcript variant X2, mRNA. | --- | Cluster0023 |

| | | | | |
|---|---|---|---|---|
| GRIN2A | 14421898 | PREDICTED: Canis lupus familiaris glutamate receptor, ionotropic, N-methyl D-aspartate 2A (GRIN2A), transcript variant X2, mRNA. | --- | Cluster0023 |
| IGSF3 | 14308988 | PREDICTED: Canis lupus familiaris immunoglobulin superfamily, member 3 (IGSF3), transcript variant X1, mRNA. | --- | Cluster0023 |
| ITGA2 | 14408032 | PREDICTED: Canis lupus familiaris integrin, alpha 2 (CD49B, alpha 2 subunit of VLA-2 receptor) (ITGA2), mRNA. | substrate-dependent cell migration // cell adhesion // cell-matrix adhesion // integrin-mediated signaling pathway // cell-substrate adhesion // cell adhesion mediated by integrin // collagen-activated signaling pathway // mesodermal cell differentiation | Cluster0023 |
| LOC474938 | 14284002 | Canis lupus familiaris glutathione S-transferase A2 (LOC474938), mRNA. | metabolic process | Cluster0023 |
| LPAR1 | 14278691 | PREDICTED: Canis lupus familiaris lysophosphatidic acid receptor 1 (LPAR1), transcript variant X1, mRNA. | signal transduction // G-protein coupled receptor signaling pathway | Cluster0023 |
| NKAIN2 | 14256708 | PREDICTED: Canis lupus familiaris Na+/K+ transporting ATPase interacting 2 (NKAIN2), transcript variant X1, mRNA. | --- | Cluster0023 |
| PCSK6 | 14374628 | PREDICTED: Canis lupus familiaris proprotein convertase subtilisin/kexin type 6 (PCSK6), transcript variant X1, mRNA. | --- | Cluster0023 |
| RASGRF2 | 14376784 | PREDICTED: Canis lupus familiaris Ras protein-specific guanine nucleotide-releasing factor 2 (RASGRF2), mRNA. | --- | Cluster0023 |
| SLCO5A1 | 14373210 | PREDICTED: Canis lupus familiaris solute carrier organic anion transporter family, member 5A1 (SLCO5A1), transcript variant X2, mRNA. | --- | Cluster0023 |
| TNMD | 14459309 | PREDICTED: Canis lupus familiaris tenomodulin (TNMD), mRNA. | endothelial cell morphogenesis // negative regulation of endothelial cell proliferation // negative regulation of angiogenesis | Cluster0023 |
| TRPM3 | 14257412 | PREDICTED: Canis lupus familiaris transient receptor potential cation channel, subfamily M, member 3 (TRPM3), transcript variant X8, mRNA. | --- | Cluster0023 |
| ZBTB14 | 14436325 | PREDICTED: Canis lupus familiaris zinc finger and BTB domain containing 14 (ZBTB14), transcript variant X3, mRNA. | --- | Cluster0023 |

| | | | | |
|---|---|---|---|---|
| --- | 14466268 | PREDICTED: Canis lupus familiaris transforming growth factor, beta receptor III (TGFBR3), transcript variant X1, mRNA. | --- | Cluster0062 |
| AURKB | 14416022 | PREDICTED: Canis lupus familiaris aurora kinase B (AURKB), transcript variant X3, mRNA. | negative regulation of transcription from RNA polymerase II promoter // negative regulation of B cell apoptotic process // protein phosphorylation // cell cycle // mitotic spindle organization // mitotic nuclear division // spindle checkpoint // negative regulation of protein binding // regulation of cytokinesis // positive regulation of cytokinesis // protein localization to kinetochore // cellular response to UV // cleavage furrow formation // spindle stabilization // histone H3-S28 phosphorylation // mitotic spindle midzone assembly | Cluster0062 |
| CCDC149 | 14376104 | PREDICTED: Canis lupus familiaris coiled-coil domain containing 149 (CCDC149), mRNA. | --- | Cluster0062 |
| CRISPLD2 | 14418313 | PREDICTED: Canis lupus familiaris cysteine-rich secretory protein LCCL domain containing 2 (CRISPLD2), mRNA. | extracellular matrix organization // face morphogenesis | Cluster0062 |
| GCNT4 | 14374421 | PREDICTED: Canis lupus familiaris glucosaminyl (N-acetyl) transferase 4, core 2 (GCNT4), transcript variant X1, mRNA. | inter-male aggressive behavior // thyroid hormone metabolic process // tissue morphogenesis // homeostasis of number of cells // kidney morphogenesis | Cluster0062 |
| IFT122 | 14332918 | PREDICTED: Canis lupus familiaris intraflagellar transport 122 (IFT122), transcript variant X2, mRNA. | --- | Cluster0062 |
| PDGFRL | 14300780 | PREDICTED: Canis lupus familiaris platelet-derived growth factor receptor-like (PDGFRL), transcript variant X1, mRNA. | --- | Cluster0062 |
| SCARA5 | 14353689 | PREDICTED: Canis lupus familiaris scavenger receptor class A, member 5 (SCARA5), transcript variant X1, mRNA. | --- | Cluster0062 |
| TGFBR3 | 14423505 | PREDICTED: Canis lupus familiaris transforming growth factor, beta receptor III (TGFBR3), transcript variant X1, mRNA. | --- | Cluster0062 |
| TNFRSF19 | 14355424 | PREDICTED: Canis lupus familiaris tumor necrosis factor receptor superfamily, member 19 (TNFRSF19), transcript variant X2, mRNA. | --- | Cluster0062 |
| ABI2 | 14398172 | PREDICTED: Canis lupus familiaris abl-interactor 2 (ABI2), transcript variant X10, mRNA. | --- | Cluster0068 |

| | | | | |
|---|---|---|---|---|
| ANK2 | 14387456 | PREDICTED: Canis lupus familiaris ankyrin 2, neuronal (ANK2), transcript variant X1, mRNA. | --- | Cluster0068 |
| LAMA2 | 14256795 | PREDICTED: Canis lupus familiaris laminin, alpha 2 (LAMA2), mRNA. | --- | Cluster0068 |
| LAMA2 | 14256801 | PREDICTED: Canis lupus familiaris laminin, alpha 2 (LAMA2), mRNA. | --- | Cluster0068 |
| LOC479922 | 14456388 | PREDICTED: Canis lupus familiaris pancreatic alpha-amylase (LOC479922), mRNA. | carbohydrate metabolic process // metabolic process | Cluster0068 |
| LOC607276 | 14455969 | PREDICTED: Canis lupus familiaris pancreatic alpha-amylase-like (LOC607276), mRNA. | --- | Cluster0068 |
| LOC607460 | 14456239 | PREDICTED: Canis lupus familiaris pancreatic alpha-amylase (LOC607460), transcript variant X2, mRNA. | carbohydrate metabolic process // metabolic process | Cluster0068 |
| LOC612019 | 14455986 | PREDICTED: Canis lupus familiaris pancreatic alpha-amylase (LOC612019), mRNA. | --- | Cluster0068 |
| MASP1 | 14393185 | PREDICTED: Canis lupus familiaris mannan-binding lectin serine peptidase 1 (C4/C2 activating component of Ra-reactive factor) (MASP1), transcript variant X1, mRNA. | --- | Cluster0068 |
| RNF207 | 14412226 | PREDICTED: Canis lupus familiaris ring finger protein 207 (RNF207), transcript variant X2, mRNA. | --- | Cluster0068 |
| --- | 14409021 | --- | --- | Cluster0102 |
| --- | 14409022 | --- | --- | Cluster0102 |
| --- | 14409023 | --- | --- | Cluster0102 |
| --- | 14467210 | PREDICTED: Canis lupus familiaris apolipoprotein L, 5 (APOL5), transcript variant X1, mRNA. | --- | Cluster0102 |
| ISOC1 | 14274280 | PREDICTED: Canis lupus familiaris isochorismatase domain containing 1 (ISOC1), mRNA. | --- | Cluster0102 |
| LRRTM4 | 14305325 | PREDICTED: Canis lupus familiaris leucine rich repeat transmembrane neuronal 4 (LRRTM4), transcript variant X1, mRNA. | --- | Cluster0102 |
| SCN3B | 14409018 | PREDICTED: Canis lupus familiaris sodium channel, voltage gated, type III beta subunit (SCN3B), transcript variant X1, mRNA. | sodium ion transport // positive regulation of heart rate // positive regulation of sodium ion transport // sodium ion transmembrane transport // membrane depolarization // cardiac | Cluster0102 |

| | | | | |
|---|---|---|---|---|
| | | | muscle contraction // regulation of atrial cardiac muscle cell membrane depolarization // regulation of ventricular cardiac muscle cell membrane depolarization // cardiac conduction // protein localization to plasma membrane // cardiac muscle cell action potential involved in contraction // ventricular cardiac muscle cell action potential // membrane depolarization during action potential // membrane depolarization during cardiac muscle cell action potential // atrial cardiac muscle cell action potential // SA node cell action potential // regulation of heart rate by cardiac conduction // regulation of sodium ion transmembrane transporter activity | |
| TCAF2 | 14299276 | PREDICTED: Canis lupus familiaris TRPM8 channel-associated factor 2 (TCAF2), mRNA. | hematopoietic progenitor cell differentiation // negative regulation of anion channel activity // positive regulation of cell migration // positive regulation of protein targeting to membrane | Cluster0102 |
| --- | 14424020 | cdna:genscan chromosome:CanFam3.1:6:74930796:74955668:1 transcript_biotype:protein_coding | --- | Cluster0106 |
| --- | 14476391 | --- | --- | Cluster0106 |
| C4H10orf54 | 14406378 | PREDICTED: Canis lupus familiaris chromosome 4 open reading frame, human C10orf54 (C4H10orf54), mRNA. | --- | Cluster0106 |
| CFH | 14401648 | PREDICTED: Canis lupus familiaris complement factor H (CFH), mRNA. | --- | Cluster0106 |
| GATSL3 | 14361524 | PREDICTED: Canis lupus familiaris GATS protein-like 3 (GATSL3), mRNA. | --- | Cluster0106 |
| MMP16 | 14373535 | PREDICTED: Canis lupus familiaris matrix metallopeptidase 16 (membrane-inserted) (MMP16), mRNA. | ossification // endochondral ossification // proteolysis // collagen catabolic process // chondrocyte proliferation // embryonic cranial skeleton morphogenesis // bone development // craniofacial suture morphogenesis | Cluster0106 |
| PI15 | 14372233 | PREDICTED: Canis lupus familiaris peptidase inhibitor 15 (PI15), transcript variant X1, mRNA. | --- | Cluster0106 |
| TMEFF2 | 14399182 | PREDICTED: Canis lupus familiaris transmembrane protein with EGF-like and two follistatin-like domains 2 (TMEFF2), mRNA. | --- | Cluster0106 |
| BRD9 | 14391405 | PREDICTED: Canis lupus familiaris bromodomain containing 9 (BRD9), mRNA. | --- | Cluster0120 |

| | | | | |
|---|---|---|---|---|
| FAM189A2 | 14263024 | PREDICTED: Canis lupus familiaris family with sequence similarity 189, member A2 (FAM189A2), transcript variant X1, mRNA. | --- | Cluster0120 |
| LOC488818 | 14375699 | PREDICTED: Canis lupus familiaris fibroblast growth factor-binding protein 1 (LOC488818), mRNA. | positive regulation of defense response to virus by host // positive regulation of cell proliferation // positive regulation of fibroblast growth factor receptor signaling pathway // mitophagy in response to mitochondrial depolarization | Cluster0120 |
| PNMT | 14451589 | PREDICTED: Canis lupus familiaris phenylethanolamine N-methyltransferase (PNMT), mRNA. | --- | Cluster0120 |
| PRPSAP1 | 14443799 | PREDICTED: Canis lupus familiaris phosphoribosyl pyrophosphate synthetase-associated protein 1 (PRPSAP1), mRNA. | nucleotide biosynthetic process | Cluster0120 |
| SMO | 14292160 | PREDICTED: Canis lupus familiaris smoothened, frizzled class receptor (SMO), mRNA. | negative regulation of transcription from RNA polymerase II promoter // ossification // vasculogenesis // osteoblast differentiation // in utero embryonic development // cell fate specification // neural crest cell migration // heart looping // positive regulation of neuroblast proliferation // positive regulation of mesenchymal cell proliferation // heart morphogenesis // determination of left/right asymmetry in lateral mesoderm // type B pancreatic cell development // cell surface receptor signaling pathway // G-protein coupled receptor signaling pathway // smoothened signaling pathway // smoothened signaling pathway // positive regulation of hh target transcription factor activity // determination of left/right symmetry // ventral midline determination // pattern specification process // central nervous system development // midgut development // positive regulation of cell proliferation // anterior/posterior pattern specification // dorsal/ventral pattern formation // regulation of gene expression // positive regulation of gene expression // negative regulation of gene expression // dentate gyrus development // cerebellar cortex morphogenesis // thalamus development // dorsal/ventral neural tube patterning // smoothened signaling pathway involved in ventral spinal cord patterning // smoothened signaling pathway involved in regulation of cerebellar granule cell precursor cell proliferation // central nervous system neuron differentiation // cerebral cortex development // negative | Cluster0120 |

| | | | | |
|---|---|---|---|---|
| | | | regulation of epithelial cell differentiation // hair follicle morphogenesis // protein localization to nucleus // multicellular organism growth // non-canonical Wnt signaling pathway // positive regulation of multicellular organism growth // positive regulation of protein import into nucleus // odontogenesis of dentin-containing tooth // negative regulation of apoptotic process // negative regulation of DNA binding // positive regulation of smoothened signaling pathway // negative regulation of transcription, DNA-templated // positive regulation of transcription, DNA-templated // positive regulation of transcription from RNA polymerase II promoter // positive regulation of organ growth // astrocyte activation // cell development // digestive tract development // embryonic organ development // developmental growth // skeletal muscle fiber development // forebrain morphogenesis // homeostasis of number of cells within a tissue // positive regulation of epithelial cell proliferation // protein stabilization // myoblast migration // negative regulation of hair follicle development // canonical Wnt signaling pathway // detection of cell density by contact stimulus involved in contact inhibition // atrial septum morphogenesis // mammary gland epithelial cell differentiation // epithelial-mesenchymal cell signaling // somite development // pancreas morphogenesis // left/right axis specification // cellular response to cholesterol // renal system development // mesenchymal to epithelial transition involved in metanephric renal vesicle formation // positive regulation of branching involved in ureteric bud morphogenesis // regulation of stem cell population maintenance // regulation of heart morphogenesis | |
| TMEM98 | 14452742 | PREDICTED: Canis lupus familiaris transmembrane protein 98 (TMEM98), mRNA. | --- | Cluster0120 |
| --- | 14465690 | cdna:genscan chromosome:CanFam3.1:4:13244792:13352365:1 transcript_biotype:protein_coding | --- | Cluster0132 |
| ALDH5A1 | 14394183 | PREDICTED: Canis lupus familiaris aldehyde dehydrogenase 5 family, member A1 (ALDH5A1), transcript variant X1, mRNA. | --- | Cluster0132 |

| | | | | |
|---|---|---|---|---|
| ENPP2 | 14287965 | PREDICTED: Canis lupus familiaris ectonucleotide pyrophosphatase/phosphodiesterase 2 (ENPP2), transcript variant X1, mRNA. | --- | Cluster0132 |
| LOC102152842 | 14304217 | PREDICTED: Canis lupus familiaris zinc finger protein 512-like (LOC102152842), mRNA. | --- | Cluster0132 |
| MPP6 | 14291147 | PREDICTED: Canis lupus familiaris membrane protein, palmitoylated 6 (MAGUK p55 subfamily member 6) (MPP6), transcript variant X1, mRNA. | --- | Cluster0132 |
| SERINC2 | 14325844 | PREDICTED: Canis lupus familiaris serine incorporator 2 (SERINC2), mRNA. | --- | Cluster0132 |
| SLC2A12 | 14261338 | PREDICTED: Canis lupus familiaris solute carrier family 2 (facilitated glucose transporter), member 12 (SLC2A12), transcript variant X2, mRNA. | --- | Cluster0132 |
| ADAMTS15 | 14413896 | PREDICTED: Canis lupus familiaris ADAM metallopeptidase with thrombospondin type 1 motif, 15 (ADAMTS15), mRNA. | --- | Cluster0133 |
| CILP | 14383643 | PREDICTED: Canis lupus familiaris cartilage intermediate layer protein, nucleotide pyrophosphohydrolase (CILP), transcript variant X2, mRNA. | --- | Cluster0133 |
| KCNQ5 | 14281354 | PREDICTED: Canis lupus familiaris potassium channel, voltage gated KQT-like subfamily Q, member 5 (KCNQ5), transcript variant X3, mRNA. | --- | Cluster0133 |
| MYOC | 14430593 | Canis lupus familiaris myocilin, trabecular meshwork inducible glucocorticoid response (MYOC), mRNA. | osteoblast differentiation // negative regulation of cell-matrix adhesion // positive regulation of phosphatidylinositol 3-kinase signaling // skeletal muscle hypertrophy // myelination in peripheral nervous system // positive regulation of cell migration // neuron projection development // negative regulation of Rho protein signal transduction // non-canonical Wnt signaling pathway via JNK cascade // ERBB2-ERBB3 signaling pathway // regulation of MAPK cascade // clustering of voltage-gated sodium channels // positive regulation of stress fiber assembly // negative regulation of stress fiber assembly // positive regulation of focal adhesion assembly // positive regulation of protein kinase B signaling // positive regulation of mitochondrial depolarization // bone development // positive regulation of substrate adhesion-dependent cell spreading // | Cluster0133 |

| | | | | |
|---|---|---|---|---|
| | | | osteoblast differentiation // regulation of cell-matrix adhesion // negative regulation of cell-matrix adhesion // positive regulation of phosphatidylinositol 3-kinase signaling // skeletal muscle hypertrophy // myelination in peripheral nervous system // positive regulation of cell migration // neuron projection development // negative regulation of Rho protein signal transduction // non-canonical Wnt signaling pathway via JNK cascade // ERBB2-ERBB3 signaling pathway // regulation of MAPK cascade // clustering of voltage-gated sodium channels // regulation of stress fiber assembly // positive regulation of stress fiber assembly // negative regulation of stress fiber assembly // positive regulation of focal adhesion assembly // positive regulation of protein kinase B signaling // positive regulation of mitochondrial depolarization // bone development // positive regulation of substrate adhesion-dependent cell spreading | |
| PDE3B | 14339116 | PREDICTED: Canis lupus familiaris phosphodiesterase 3B, cGMP-inhibited (PDE3B), mRNA. | --- | Cluster0133 |
| PTGFR | 14428802 | Canis lupus familiaris prostaglandin F receptor (FP) (PTGFR), mRNA. | signal transduction // G-protein coupled receptor signaling pathway // positive regulation of cell proliferation // positive regulation of gene expression // response to estradiol // response to lipopolysaccharide // calcium-mediated signaling using intracellular calcium source // negative regulation of apoptotic process // cellular response to prostaglandin D stimulus | Cluster0133 |
| SCNN1B | 14426126 | Canis lupus familiaris sodium channel, non voltage gated 1 beta subunit (SCNN1B), mRNA. | sodium ion transmembrane transport // multicellular organismal water homeostasis // response to stimulus // sensory perception of taste // sodium ion homeostasis | Cluster0133 |
| | | | | |
| **High in all MMVD** | | | | |
| **Name** | | **mRNA - Description** | **GO Biological Process Term** | **MCL_1.7_6** |
| --- | 14259561 | PREDICTED: Canis lupus familiaris zinc finger protein 404 (ZNF404), transcript variant X1, mRNA. | --- | Cluster0003 |

| | | | | |
|---|---|---|---|---|
| --- | 14377323 | Small nucleolar RNA SNORD116 [gene_biotype:snoRNA transcript_biotype:snoRNA] | --- | Cluster0003 |
| --- | 14459085 | cdna:genscan chromosome:CanFam3.1:X:57469224:57470501:1 transcript_biotype:protein_coding | --- | Cluster0003 |
| --- | 14465464 | Canis lupus familiaris H2A histone family, member Z (H2AFZ), mRNA. | --- | Cluster0003 |
| --- | 14466418 | PREDICTED: Canis lupus familiaris SET nuclear proto-oncogene (SET), mRNA. | --- | Cluster0003 |
| --- | 14466980 | Canis lupus familiaris prefoldin subunit 6 (PFDN6), mRNA. | --- | Cluster0003 |
| --- | 14467408 | Canis lupus familiaris prefoldin subunit 1 (PFDN1), mRNA. | --- | Cluster0003 |
| --- | 14467590 | Canis lupus familiaris chloride intracellular channel 1 (CLIC1), mRNA. | --- | Cluster0003 |
| --- | 14467592 | Canis lupus familiaris chloride intracellular channel 1 (CLIC1), mRNA. | --- | Cluster0003 |
| --- | 14468598 | Canis lupus familiaris prefoldin subunit 1 (PFDN1), mRNA. | --- | Cluster0003 |
| --- | 14469012 | ensembl:novel chromosome:CanFam3.1:20:41386620:41387110:1 gene:ENSCAFG00000004560 gene_biotype:pseudogene transcript_biotype:pseudogene | --- | Cluster0003 |
| --- | 14471498 | PREDICTED: Canis lupus familiaris UPF3 regulator of nonsense transcripts homolog B (yeast) (UPF3B), transcript variant X2, mRNA. | --- | Cluster0003 |
| --- | 14472630 | Canis lupus familiaris ATM serine/threonine kinase (ATM), mRNA. | --- | Cluster0003 |
| --- | 14473436 | Canis lupus familiaris ATM serine/threonine kinase (ATM), mRNA. | --- | Cluster0003 |
| --- | 14473748 | Canis lupus familiaris ATM serine/threonine kinase (ATM), mRNA. | --- | Cluster0003 |
| AP3B1 | 14374308 | Canis lupus familiaris adaptor-related protein complex 3, beta 1 subunit (AP3B1), mRNA. | intracellular protein transport // anterograde axon cargo transport // anterograde synaptic vesicle transport // transport // protein transport // vesicle-mediated transport | Cluster0003 |

| | | | | |
|---|---|---|---|---|
| ATRAID | 14304082 | PREDICTED: Canis lupus familiaris all-trans retinoic acid-induced differentiation factor (ATRAID), mRNA. | regulation of gene expression // positive regulation of bone mineralization // negative regulation of osteoblast proliferation // positive regulation of osteoblast differentiation // negative regulation of cyclin catabolic process | Cluster0003 |
| BIVM | 14342479 | Canis lupus familiaris basic, immunoglobulin-like variable motif containing (BIVM), mRNA. | --- | Cluster0003 |
| C21H11orf73 | 14339931 | PREDICTED: Canis lupus familiaris chromosome 21 open reading frame, human C11orf73 (C21H11orf73), transcript variant X1, mRNA. | protein import into nucleus // Golgi organization // protein transport // lung development // cellular response to heat | Cluster0003 |
| CCT6A | 14419526 | PREDICTED: Canis lupus familiaris chaperonin containing TCP1, subunit 6A (zeta 1) (CCT6A), mRNA. | --- | Cluster0003 |
| CHMP2A | 14257953 | PREDICTED: Canis lupus familiaris charged multivesicular body protein 2A (CHMP2A), transcript variant X2, mRNA. | --- | Cluster0003 |
| CHMP5 | 14275185 | PREDICTED: Canis lupus familiaris charged multivesicular body protein 5 (CHMP5), mRNA. | cell separation after cytokinesis // regulation of receptor recycling // nucleus organization // vacuolar transport // lysosome organization // mitotic metaphase plate congression // endosome to lysosome transport // regulation of centrosome duplication // viral budding // multivesicular body sorting pathway // regulation of mitotic spindle assembly | Cluster0003 |
| CHORDC1 | 14337606 | PREDICTED: Canis lupus familiaris cysteine and histidine-rich domain (CHORD) containing 1 (CHORDC1), mRNA. | regulation of centrosome duplication // chaperone-mediated protein folding // regulation of cellular response to heat // negative regulation of Rho-dependent protein serine/threonine kinase activity | Cluster0003 |
| CNIH1 | 14441461 | PREDICTED: Canis lupus familiaris cornichon family AMPA receptor auxiliary protein 1 (CNIH1), transcript variant X2, mRNA. | --- | Cluster0003 |
| CS | 14270051 | PREDICTED: Canis lupus familiaris citrate synthase (CS), transcript variant X1, mRNA. | --- | Cluster0003 |
| EDF1 | 14448127 | PREDICTED: Canis lupus familiaris endothelial differentiation-related factor 1 (EDF1), mRNA. | --- | Cluster0003 |
| EEA1 | 14298273 | PREDICTED: Canis lupus familiaris early endosome antigen 1 (EEA1), transcript variant X1, mRNA. | --- | Cluster0003 |
| EIF2AK3 | 14305105 | PREDICTED: Canis lupus familiaris eukaryotic translation initiation factor 2-alpha kinase 3 (EIF2AK3), transcript variant X2, mRNA. | --- | Cluster0003 |

| | | | | |
|---|---|---|---|---|
| FAM204A | 14371277 | PREDICTED: Canis lupus familiaris family with sequence similarity 204, member A (FAM204A), transcript variant X2, mRNA. | --- | Cluster0003 |
| FAM98A | 14307675 | PREDICTED: Canis lupus familiaris family with sequence similarity 98, member A (FAM98A), mRNA. | --- | Cluster0003 |
| FCF1 | 14438702 | PREDICTED: Canis lupus familiaris FCF1 rRNA-processing protein (FCF1), mRNA. | --- | Cluster0003 |
| GOLGA4 | 14344261 | PREDICTED: Canis lupus familiaris golgin A4 (GOLGA4), mRNA. | --- | Cluster0003 |
| GPR180 | 14342275 | PREDICTED: Canis lupus familiaris G protein-coupled receptor 180 (GPR180), mRNA. | --- | Cluster0003 |
| GPR89A | 14305980 | PREDICTED: Canis lupus familiaris G protein-coupled receptor 89A (GPR89A), transcript variant X1, mRNA. | --- | Cluster0003 |
| IPO7 | 14338847 | PREDICTED: Canis lupus familiaris importin 7 (IPO7), mRNA. | protein import into nucleus // protein import into nucleus // intracellular protein transport // signal transduction // innate immune response | Cluster0003 |
| KTN1 | 14437694 | PREDICTED: Canis lupus familiaris kinectin 1 (kinesin receptor) (KTN1), transcript variant X1, mRNA. | --- | Cluster0003 |
| LLPH | 14270564 | PREDICTED: Canis lupus familiaris LLP homolog, long-term synaptic facilitation (Aplysia) (LLPH), mRNA. | --- | Cluster0003 |
| LOC102151205 | 14377325 | PREDICTED: Canis lupus familiaris uncharacterized LOC102151205 (LOC102151205), transcript variant X18, ncRNA. | --- | Cluster0003 |
| LOC102155476 | 14270515 | PREDICTED: Canis lupus familiaris COX assembly mitochondrial protein 2 homolog (LOC102155476), mRNA. | --- | Cluster0003 |
| LOC480934 | 14462512 | PREDICTED: Canis lupus familiaris melanoma-associated antigen D2 (LOC480934), transcript variant X3, mRNA. | --- | Cluster0003 |
| MANF | 14334256 | PREDICTED: Canis lupus familiaris mesencephalic astrocyte-derived neurotrophic factor (MANF), mRNA. | --- | Cluster0003 |
| MNF1 | 14282987 | PREDICTED: Canis lupus familiaris mitochondrial nucleoid factor 1 (MNF1), mRNA. | regulation of oxidative phosphorylation // mitochondrial respiratory chain complex III assembly // regulation of insulin | Cluster0003 |

| | | | | |
|---|---|---|---|---|
| | | | secretion // positive regulation of mitochondrial translation // positive regulation of cellular protein catabolic process // regulation of skeletal muscle cell differentiation | |
| MTX2 | 14395875 | PREDICTED: Canis lupus familiaris metaxin 2 (MTX2), transcript variant X1, mRNA. | --- | Cluster0003 |
| NARS | 14255337 | PREDICTED: Canis lupus familiaris asparaginyl-tRNA synthetase (NARS), transcript variant X2, mRNA. | --- | Cluster0003 |
| PAM16 | 14422129 | Canis lupus familiaris presequence translocase-associated motor 16 homolog (S. cerevisiae) (PAM16), mRNA. | ossification // protein import into mitochondrial matrix // protein import into mitochondrial matrix // negative regulation of ATPase activity | Cluster0003 |
| PDCD2 | 14285574 | PREDICTED: Canis lupus familiaris programmed cell death 2 (PDCD2), mRNA. | activation of cysteine-type endopeptidase activity involved in apoptotic process // positive regulation of apoptotic process // regulation of hematopoietic progenitor cell differentiation // positive regulation of hematopoietic stem cell proliferation | Cluster0003 |
| PLA2G4A | 14430287 | PREDICTED: Canis lupus familiaris phospholipase A2, group IVA (cytosolic, calcium-dependent) (PLA2G4A), transcript variant X5, mRNA. | lipid metabolic process // metabolic process // phospholipid catabolic process // lipid catabolic process // regulation of cell proliferation // icosanoid biosynthetic process // arachidonic acid secretion // cellular response to antibiotic | Cluster0003 |
| PRDM5 | 14317109 | PREDICTED: Canis lupus familiaris PR domain containing 5 (PRDM5), mRNA. | --- | Cluster0003 |
| RAD23B | 14276016 | PREDICTED: Canis lupus familiaris RAD23 homolog B, nucleotide excision repair protein (RAD23B), mRNA. | nucleotide-excision repair, DNA damage recognition // nucleotide-excision repair // cellular response to DNA damage stimulus // spermatogenesis // regulation of proteasomal ubiquitin-dependent protein catabolic process // proteasome-mediated ubiquitin-dependent protein catabolic process | Cluster0003 |
| RNASEH2C | 14312426 | PREDICTED: Canis lupus familiaris ribonuclease H2, subunit C (RNASEH2C), mRNA. | RNA catabolic process // RNA catabolic process // RNA phosphodiester bond hydrolysis, endonucleolytic | Cluster0003 |
| RPA3 | 14276477 | replication protein A3, 14kDa [gene_biotype:protein_coding transcript_biotype:protein_coding] | DNA replication // double-strand break repair via homologous recombination // DNA repair // base-excision repair // nucleotide-excision repair // mismatch repair // DNA recombination // regulation of mitotic cell cycle // regulation of cell proliferation | Cluster0003 |
| RPF1 | 14428717 | PREDICTED: Canis lupus familiaris ribosome production factor 1 homolog (RPF1), mRNA. | ribosomal large subunit assembly // maturation of 5.8S rRNA // maturation of LSU-rRNA | Cluster0003 |
| RPL36AL | 14441140 | Canis lupus familiaris ribosomal protein L36a-like (RPL36AL), mRNA. | translation | Cluster0003 |

| | | | | |
|---|---|---|---|---|
| RPS27L | 14383429 | PREDICTED: Canis lupus familiaris ribosomal protein S27-like (RPS27L), mRNA. | --- | Cluster0003 |
| SCYL2 | 14295592 | PREDICTED: Canis lupus familiaris SCY1-like, kinase-like 2 (SCYL2), transcript variant X1, mRNA. | --- | Cluster0003 |
| SKA2 | 14452355 | PREDICTED: Canis lupus familiaris spindle and kinetochore associated complex subunit 2 (SKA2), mRNA. | chromosome segregation // mitotic nuclear division // regulation of microtubule polymerization or depolymerization // cell division | Cluster0003 |
| SKP1 | 14276994 | Canis lupus familiaris S-phase kinase-associated protein 1 (SKP1), mRNA. | --- | Cluster0003 |
| SNRPE | 14400491 | PREDICTED: Canis lupus familiaris small nuclear ribonucleoprotein polypeptide E (SNRPE), mRNA. | --- | Cluster0003 |
| SNX4 | 14390864 | PREDICTED: Canis lupus familiaris sorting nexin 4 (SNX4), mRNA. | endocytosis // protein transport // vesicle organization // endocytic recycling // positive regulation of histamine secretion by mast cell | Cluster0003 |
| SRP54 | 14437259 | Canis lupus familiaris signal recognition particle 54kDa (SRP54), mRNA. | SRP-dependent cotranslational protein targeting to membrane, translocation // SRP-dependent cotranslational protein targeting to membrane, signal sequence recognition // metabolic process // response to drug // protein targeting to ER // protein targeting to ER // SRP-dependent cotranslational protein targeting to membrane // response to drug // protein targeting to ER | Cluster0003 |
| SS18 | 14432470 | PREDICTED: Canis lupus familiaris synovial sarcoma translocation, chromosome 18 (SS18), transcript variant X7, mRNA. | --- | Cluster0003 |
| STX5 | 14312905 | PREDICTED: Canis lupus familiaris syntaxin 5 (STX5), transcript variant X6, mRNA. | intracellular protein transport // vesicle-mediated transport // early endosome to Golgi transport // retrograde transport, endosome to Golgi // positive regulation of protein catabolic process // Golgi disassembly // regulation of Golgi organization | Cluster0003 |
| TARS | 14408473 | PREDICTED: Canis lupus familiaris threonyl-tRNA synthetase (TARS), mRNA. | tRNA aminoacylation for protein translation // threonyl-tRNA aminoacylation // tRNA aminoacylation | Cluster0003 |
| TMEM258 | 14428778 | transmembrane protein 258 [gene_biotype:protein_coding transcript_biotype:protein_coding] | --- | Cluster0003 |
| TMF1 | 14327937 | PREDICTED: Canis lupus familiaris TATA element modulatory factor 1 (TMF1), mRNA. | --- | Cluster0003 |

| | | | | |
|---|---|---|---|---|
| TOMM6 | 14280422 | PREDICTED: Canis lupus familiaris translocase of outer mitochondrial membrane 6 homolog (yeast) (TOMM6), transcript variant X2, mRNA. | protein targeting to mitochondrion | Cluster0003 |
| TPR | 14433934 | PREDICTED: Canis lupus familiaris translocated promoter region, nuclear basket protein (TPR), mRNA. | --- | Cluster0003 |
| UBQLN1 | 14257187 | PREDICTED: Canis lupus familiaris ubiquilin 1 (UBQLN1), transcript variant X1, mRNA. | regulation of macroautophagy // ER-associated ubiquitin-dependent protein catabolic process // regulation of protein ubiquitination // positive regulation of protein ubiquitination // negative regulation of toll-like receptor 3 signaling pathway // response to endoplasmic reticulum stress // aggrephagy // cellular response to hypoxia // negative regulation of autophagosome maturation // negative regulation of store-operated calcium channel activity // regulation of oxidative stress-induced intrinsic apoptotic signaling pathway // positive regulation of ER-associated ubiquitin-dependent protein catabolic process // regulation of autophagosome assembly | Cluster0003 |
| USP12 | 14353145 | PREDICTED: Canis lupus familiaris ubiquitin specific peptidase 12 (USP12), transcript variant X2, mRNA. | --- | Cluster0003 |
| ZFYVE9 | 14297084 | PREDICTED: Canis lupus familiaris zinc finger, FYVE domain containing 9 (ZFYVE9), transcript variant X3, mRNA. | --- | Cluster0003 |
| --- | 14468060 | PREDICTED: Canis lupus familiaris CAP, adenylate cyclase-associated protein 1 (yeast) (CAP1), transcript variant X2, mRNA. | --- | Cluster0006 |
| --- | 14468062 | PREDICTED: Canis lupus familiaris CAP, adenylate cyclase-associated protein 1 (yeast) (CAP1), transcript variant X2, mRNA. | --- | Cluster0006 |
| --- | 14468064 | PREDICTED: Canis lupus familiaris CAP, adenylate cyclase-associated protein 1 (yeast) (CAP1), transcript variant X2, mRNA. | --- | Cluster0006 |
| --- | 14468066 | PREDICTED: Canis lupus familiaris CAP, adenylate cyclase-associated protein 1 (yeast) (CAP1), transcript variant X2, mRNA. | --- | Cluster0006 |
| --- | 14472396 | PREDICTED: Canis lupus familiaris thyroid hormone receptor, beta (THRB), transcript variant X1, mRNA. | --- | Cluster0006 |

| | | | | |
|---|---|---|---|---|
| --- | 14472956 | PREDICTED: Canis lupus familiaris thyroid hormone receptor, beta (THRB), transcript variant X1, mRNA. | --- | Cluster0006 |
| --- | 14474168 | PREDICTED: Canis lupus familiaris thyroid hormone receptor, beta (THRB), transcript variant X1, mRNA. | --- | Cluster0006 |
| ALCAM | 14389102 | Canis lupus familiaris activated leukocyte cell adhesion molecule (ALCAM), mRNA. | cell adhesion // heterophilic cell-cell adhesion via plasma membrane cell adhesion molecules // retinal ganglion cell axon guidance // axon extension involved in axon guidance // neuron projection extension // cell adhesion | Cluster0006 |
| ALDH1L2 | 14268673 | PREDICTED: Canis lupus familiaris aldehyde dehydrogenase 1 family, member L2 (ALDH1L2), mRNA. | one-carbon metabolic process // metabolic process // biosynthetic process // 10-formyltetrahydrofolate catabolic process // methylation // oxidation-reduction process | Cluster0006 |
| ANGPT1 | 14287764 | Canis lupus familiaris angiopoietin 1 (ANGPT1), mRNA. | angiogenesis // cell differentiation // Tie signaling pathway // multicellular organismal development | Cluster0006 |
| ARAP2 | 14376000 | PREDICTED: Canis lupus familiaris ArfGAP with RhoGAP domain, ankyrin repeat and PH domain 2 (ARAP2), transcript variant X1, mRNA. | --- | Cluster0006 |
| ARNTL2 | 14366241 | PREDICTED: Canis lupus familiaris aryl hydrocarbon receptor nuclear translocator-like 2 (ARNTL2), transcript variant X1, mRNA. | --- | Cluster0006 |
| CCDC115 | 14353391 | PREDICTED: Canis lupus familiaris coiled-coil domain containing 115 (CCDC115), transcript variant X1, mRNA. | --- | Cluster0006 |
| CDKN2A | 14277602 | Uncharacterized protein [gene_biotype:protein_coding transcript_biotype:protein_coding] | phosphorylation | Cluster0006 |
| CDKN2A | 14277604 | Canis lupus familiaris cyclin-dependent kinase inhibitor 2A mRNA, partial cds. | apoptotic process // cell cycle arrest // negative regulation of cell proliferation // phosphorylation | Cluster0006 |
| DHRS7 | 14441661 | PREDICTED: Canis lupus familiaris dehydrogenase/reductase (SDR family) member 7 (DHRS7), mRNA. | metabolic process // oxidation-reduction process | Cluster0006 |
| DSTN | 14350497 | PREDICTED: Canis lupus familiaris destrin (actin depolymerizing factor) (DSTN), mRNA. | --- | Cluster0006 |
| DZIP1 | 14343496 | Canis lupus familiaris DAZ interacting zinc finger protein 1 (DZIP1), mRNA. | --- | Cluster0006 |

| | | | | |
|---|---|---|---|---|
| FAM118B | 14414017 | PREDICTED: Canis lupus familiaris family with sequence similarity 118, member B (FAM118B), transcript variant X2, mRNA. | --- | Cluster0006 |
| KCNMB1 | 14404160 | Canis lupus familiaris potassium channel subfamily M regulatory beta subunit 1 (KCNMB1), mRNA. | potassium ion transmembrane transport // transport // ion transport // potassium ion transport | Cluster0006 |
| LRRN1 | 14333421 | PREDICTED: Canis lupus familiaris leucine rich repeat neuronal 1 (LRRN1), transcript variant X1, mRNA. | axonogenesis // positive regulation of synapse assembly | Cluster0006 |
| MRPL37 | 14417211 | PREDICTED: Canis lupus familiaris mitochondrial ribosomal protein L37 (MRPL37), mRNA. | translation | Cluster0006 |
| NHP2 | 14273910 | PREDICTED: Canis lupus familiaris NHP2 ribonucleoprotein (NHP2), transcript variant X1, mRNA. | --- | Cluster0006 |
| NME1 | 14446478 | Canis lupus familiaris non-metastatic cells 1, protein (NM23A) expressed in (NME1), mRNA. | purine nucleotide metabolic process // nucleoside diphosphate phosphorylation // GTP biosynthetic process // pyrimidine nucleotide metabolic process // UTP biosynthetic process // CTP biosynthetic process // endocytosis // nervous system development // nucleoside triphosphate biosynthetic process // cell differentiation // regulation of apoptotic process // purine nucleotide metabolic process // pyrimidine nucleotide metabolic process // nucleotide metabolic process // nucleoside triphosphate biosynthetic process // phosphorylation // regulation of apoptotic process | Cluster0006 |
| NOV | 14285991 | PREDICTED: Canis lupus familiaris nephroblastoma overexpressed (NOV), mRNA. | regulation of cell growth // cell adhesion // signal transduction // cell-cell signaling // negative regulation of NF-kappaB import into nucleus // negative regulation of inflammatory response // negative regulation of cell death // negative regulation of monocyte chemotaxis | Cluster0006 |
| PARP8 | 14408100 | PREDICTED: Canis lupus familiaris poly (ADP-ribose) polymerase family, member 8 (PARP8), transcript variant X1, mRNA. | metabolic process | Cluster0006 |
| PLCB1 | 14350654 | PREDICTED: Canis lupus familiaris phospholipase C, beta 1 (phosphoinositide-specific) (PLCB1), mRNA. | --- | Cluster0006 |

| | | | | |
|---|---|---|---|---|
| PLCB1 | 14350681 | PREDICTED: Canis lupus familiaris phospholipase C, beta 1 (phosphoinositide-specific) (PLCB1), mRNA. | --- | Cluster0006 |
| PTPRO | 14366538 | PREDICTED: Canis lupus familiaris protein tyrosine phosphatase, receptor type, O (PTPRO), transcript variant X1, mRNA. | cell morphogenesis // cell morphogenesis // monocyte chemotaxis // monocyte chemotaxis // regulation of glomerular filtration // regulation of glomerular filtration // negative regulation of glomerular filtration // protein dephosphorylation // axon guidance // axon guidance // dephosphorylation // lamellipodium assembly // lamellipodium assembly // glomerulus development // glomerulus development // peptidyl-tyrosine dephosphorylation // peptidyl-tyrosine dephosphorylation // slit diaphragm assembly // slit diaphragm assembly // glomerular visceral epithelial cell differentiation // glomerular visceral epithelial cell differentiation // negative regulation of canonical Wnt signaling pathway // negative regulation of canonical Wnt signaling pathway | Cluster0006 |
| SERPINE1 | 14424545 | Canis lupus familiaris serpin peptidase inhibitor, clade E (nexin, plasminogen activator inhibitor type 1), member 1 (SERPINE1), mRNA. | chronological cell aging // angiogenesis // regulation of receptor activity // negative regulation of plasminogen activation // negative regulation of endopeptidase activity // negative regulation of endopeptidase activity // negative regulation of smooth muscle cell migration // positive regulation of blood coagulation // negative regulation of cell migration // positive regulation of interleukin-8 production // negative regulation of cell adhesion mediated by integrin // positive regulation of leukotriene production involved in inflammatory response // positive regulation of angiogenesis // positive regulation of receptor-mediated endocytosis // positive regulation of inflammatory response // defense response to Gram-negative bacterium // negative regulation of fibrinolysis // negative regulation of vascular wound healing // cellular response to lipopolysaccharide // positive regulation of monocyte chemotaxis // negative regulation of extrinsic apoptotic signaling pathway via death domain receptors // negative regulation of smooth muscle cell-matrix adhesion // negative regulation of endothelial cell apoptotic process | Cluster0006 |
| SLC26A2 | 14407861 | PREDICTED: Canis lupus familiaris solute carrier family 26 (anion exchanger), member 2 (SLC26A2), mRNA. | ossification // transport // ion transport // sulfate transport // bicarbonate transport // oxalate transport // regulation of membrane potential // regulation of intracellular pH // transmembrane transport // sulfate transmembrane transport // | Cluster0006 |

| | | | | |
|---|---|---|---|---|
| | | | sulfate transmembrane transport // chloride transmembrane transport | |
| SLC45A1 | 14412351 | PREDICTED: Canis lupus familiaris solute carrier family 45, member 1 (SLC45A1), transcript variant X1, mRNA. | --- | Cluster0006 |
| SYNC | 14321695 | PREDICTED: Canis lupus familiaris syncoilin, intermediate filament protein (SYNC), mRNA. | --- | Cluster0006 |
| SYNDIG1 | 14344008 | PREDICTED: Canis lupus familiaris synapse differentiation inducing 1 (SYNDIG1), transcript variant X1, mRNA. | intracellular protein transport // response to biotic stimulus // positive regulation of synapse assembly | Cluster0006 |
| SYTL2 | 14337697 | PREDICTED: Canis lupus familiaris synaptotagmin-like 2 (SYTL2), transcript variant X11, mRNA. | --- | Cluster0006 |
| THRB | 14344821 | PREDICTED: Canis lupus familiaris thyroid hormone receptor, beta (THRB), transcript variant X1, mRNA. | negative regulation of transcription from RNA polymerase II promoter // transcription, DNA-templated // regulation of transcription, DNA-templated // regulation of transcription from RNA polymerase II promoter // sensory perception of sound // negative regulation of female receptivity // regulation of heart contraction // female courtship behavior // organ morphogenesis // intracellular receptor signaling pathway // steroid hormone mediated signaling pathway // negative regulation of transcription, DNA-templated // positive regulation of transcription from RNA polymerase II promoter // Type I pneumocyte differentiation | Cluster0006 |
| --- | 14279408 | leukocyte specific transcript 1 [gene_biotype:protein_coding transcript_biotype:protein_coding] | cell morphogenesis // immune response // dendrite development // regulation of lymphocyte proliferation | Cluster0011 |
| --- | 14383532 | cdna:genscan chromosome:CanFam3.1:30:28583237:28597671:-1 transcript_biotype:protein_coding | --- | Cluster0011 |
| --- | 14466994 | PREDICTED: Canis lupus familiaris regakine-1-like (LOC480601), mRNA. | --- | Cluster0011 |
| BLNK | 14370194 | PREDICTED: Canis lupus familiaris B-cell linker (BLNK), transcript variant X1, mRNA. | --- | Cluster0011 |
| BLVRB | 14259926 | PREDICTED: Canis lupus familiaris biliverdin reductase B (BLVRB), mRNA. | heme catabolic process // oxidation-reduction process | Cluster0011 |

| | | | | |
|---|---|---|---|---|
| BTK | 14463137 | PREDICTED: Canis lupus familiaris Bruton agammaglobulinemia tyrosine kinase (BTK), transcript variant X2, mRNA. | negative regulation of cytokine production // adaptive immune response // protein phosphorylation // transmembrane receptor protein tyrosine kinase signaling pathway // I-kappaB kinase/NF-kappaB signaling // phosphorylation // peptidyl-tyrosine phosphorylation // cell differentiation // intracellular signal transduction // peptidyl-tyrosine autophosphorylation // regulation of cell proliferation // innate immune response // cell maturation // T cell receptor signaling pathway // B cell receptor signaling pathway | Cluster0011 |
| CCL13 | 14452716 | Canis lupus familiaris chemokine (C-C motif) ligand 13 (CCL13), mRNA. | inflammatory response // immune response // cell chemotaxis // chemotaxis | Cluster0011 |
| CD48 | 14401130 | PREDICTED: Canis lupus familiaris CD48 molecule (CD48), mRNA. | signal transduction // T cell activation | Cluster0011 |
| CD53 | 14427829 | PREDICTED: Canis lupus familiaris CD53 molecule (CD53), mRNA. | cell surface receptor signaling pathway // positive regulation of myoblast fusion | Cluster0011 |
| DRAM2 | 14422937 | PREDICTED: Canis lupus familiaris DNA-damage regulated autophagy modulator 2 (DRAM2), transcript variant X2, mRNA. | regulation of autophagy | Cluster0011 |
| EVI2B | 14447242 | PREDICTED: Canis lupus familiaris ecotropic viral integration site 2B (EVI2B), transcript variant X1, mRNA. | --- | Cluster0011 |
| FAM49B | 14288215 | PREDICTED: Canis lupus familiaris family with sequence similarity 49, member B (FAM49B), transcript variant X1, mRNA. | --- | Cluster0011 |
| FCGR1A | 14306037 | Canis lupus familiaris Fc fragment of IgG, high affinity Ia, receptor (CD64) (FCGR1A), mRNA. | --- | Cluster0011 |
| IGSF6 | 14421355 | PREDICTED: Canis lupus familiaris immunoglobulin superfamily, member 6 (IGSF6), transcript variant X1, mRNA. | --- | Cluster0011 |
| IL18 | 14409533 | Canis lupus familiaris interleukin 18 (IL18), mRNA. | extrinsic apoptotic signaling pathway via death domain receptors // interferon-gamma biosynthetic process // positive regulation of activated T cell proliferation | Cluster0011 |
| KCNJ15 | 14384937 | PREDICTED: Canis lupus familiaris potassium channel, inwardly rectifying subfamily J, member 15 (KCNJ15), transcript variant X1, mRNA. | transport // ion transport // potassium ion transport // potassium ion import // regulation of ion transmembrane transport // regulation of ion transmembrane transport | Cluster0011 |
| LOC478984 | 14400997 | PREDICTED: Canis lupus familiaris low affinity immunoglobulin gamma Fc region receptor III (LOC478984), transcript variant X1, mRNA. | --- | Cluster0011 |

| | | | | |
|---|---|---|---|---|
| LOC607205 | 14260868 | PREDICTED: Canis lupus familiaris serpin B8 (LOC607205), transcript variant X2, mRNA. | --- | Cluster0011 |
| LOC611446 | 14258321 | PREDICTED: Canis lupus familiaris leukocyte immunoglobulin-like receptor subfamily A member 6 (LOC611446), transcript variant X3, mRNA. | --- | Cluster0011 |
| LOC612564 | 14339519 | PREDICTED: Canis lupus familiaris membrane-spanning 4-domains subfamily A member 7 (LOC612564), mRNA. | --- | Cluster0011 |
| LRRC25 | 14329923 | PREDICTED: Canis lupus familiaris leucine rich repeat containing 25 (LRRC25), transcript variant X2, mRNA. | --- | Cluster0011 |
| OSBPL11 | 14390880 | PREDICTED: Canis lupus familiaris oxysterol binding protein-like 11 (OSBPL11), mRNA. | transport // lipid transport // positive regulation of sequestering of triglyceride // fat cell differentiation | Cluster0011 |
| PTPRC | 14433437 | PREDICTED: Canis lupus familiaris protein tyrosine phosphatase, receptor type, C (PTPRC), transcript variant X2, mRNA. | protein dephosphorylation // dephosphorylation // peptidyl-tyrosine dephosphorylation // T cell receptor signaling pathway | Cluster0011 |
| RGS18 | 14401730 | PREDICTED: Canis lupus familiaris regulator of G-protein signaling 18 (RGS18), mRNA. | G-protein coupled receptor signaling pathway // regulation of G-protein coupled receptor protein signaling pathway // positive regulation of GTPase activity | Cluster0011 |
| SHTN1 | 14371238 | PREDICTED: Canis lupus familiaris shootin 1 (SHTN1), transcript variant X3, mRNA. | --- | Cluster0011 |
| TLR8 | 14457347 | PREDICTED: Canis lupus familiaris toll-like receptor 8 (TLR8), transcript variant X2, mRNA. | MyD88-dependent toll-like receptor signaling pathway // inflammatory response // toll-like receptor 8 signaling pathway // positive regulation of innate immune response // regulation of cytokine secretion // defense response to virus // regulation of protein phosphorylation // immune system process // signal transduction // I-kappaB kinase/NF-kappaB signaling // response to virus // positive regulation of interferon-gamma biosynthetic process // innate immune response // positive regulation of interferon-alpha biosynthetic process // positive regulation of interferon-beta biosynthetic process // positive regulation of interleukin-8 biosynthetic process // cellular response to mechanical stimulus | Cluster0011 |
| TPD52 | 14373402 | PREDICTED: Canis lupus familiaris tumor protein D52 (TPD52), transcript variant X1, mRNA. | --- | Cluster0011 |
| TYROBP | 14260358 | Canis lupus familiaris TYRO protein tyrosine kinase binding protein (TYROBP), mRNA. | phosphorylation | Cluster0011 |

| | | | | |
|---|---|---|---|---|
| --- | 14468652 | PREDICTED: Canis lupus familiaris putative homeodomain transcription factor 2 (PHTF2), transcript variant X1, mRNA. | --- | Cluster0018 |
| --- | 14476981 | --- | --- | Cluster0018 |
| ADAM28 | 14356154 | PREDICTED: Canis lupus familiaris ADAM metallopeptidase domain 28 (ADAM28), transcript variant X2, mRNA. | --- | Cluster0018 |
| C10H12orf45 | 14272103 | PREDICTED: Canis lupus familiaris chromosome 10 open reading frame, human C12orf45 (C10H12orf45), mRNA. | --- | Cluster0018 |
| CD163 | 14364512 | Canis lupus familiaris CD163 molecule (CD163), transcript variant 1, mRNA. | receptor-mediated endocytosis // acute-phase response // inflammatory response | Cluster0018 |
| CFB | 14279522 | PREDICTED: Canis lupus familiaris complement factor B (CFB), mRNA. | proteolysis // complement activation | Cluster0018 |
| DKK1 | 14362217 | PREDICTED: Canis lupus familiaris dickkopf WNT signaling pathway inhibitor 1 (DKK1), mRNA. | --- | Cluster0018 |
| KCNK2 | 14429810 | PREDICTED: Canis lupus familiaris potassium channel, two pore domain subfamily K, member 2 (KCNK2), transcript variant X3, mRNA. | transport // ion transport // potassium ion transport // G-protein coupled receptor signaling pathway // stabilization of membrane potential // regulation of membrane potential // potassium ion transmembrane transport // potassium ion transmembrane transport // potassium ion transmembrane transport | Cluster0018 |
| KYNU | 14317645 | PREDICTED: Canis lupus familiaris kynureninase (KYNU), transcript variant X2, mRNA. | tryptophan catabolic process // NAD biosynthetic process // pyridine nucleotide biosynthetic process // quinolinate biosynthetic process // response to interferon-gamma // de novo NAD biosynthetic process from tryptophan // response to vitamin B6 // anthranilate metabolic process // L-kynurenine catabolic process | Cluster0018 |
| LYN | 14371880 | PREDICTED: Canis lupus familiaris LYN proto-oncogene, Src family tyrosine kinase (LYN), transcript variant X2, mRNA. | --- | Cluster0018 |
| NCF2 | 14433833 | Canis lupus familiaris neutrophil cytosolic factor 2 (NCF2), mRNA. | superoxide anion generation | Cluster0018 |
| NLRP1 | 14410018 | PREDICTED: Canis lupus familiaris NLR family, pyrin domain containing 1 (NLRP1), transcript variant X1, mRNA. | --- | Cluster0018 |

| | | | | |
|---|---|---|---|---|
| NLRP3 | 14436606 | PREDICTED: Canis lupus familiaris NLR family, pyrin domain containing 3 (NLRP3), transcript variant X2, mRNA. | --- | Cluster0018 |
| PIK3CG | 14313543 | PREDICTED: Canis lupus familiaris phosphatidylinositol-4,5-bisphosphate 3-kinase, catalytic subunit gamma (PIK3CG), transcript variant X1, mRNA. | regulation of protein phosphorylation // protein phosphorylation // G-protein coupled receptor signaling pathway // phosphatidylinositol 3-kinase signaling // phosphorylation // positive regulation of catalytic activity // positive regulation of MAP kinase activity // phosphatidylinositol phosphorylation // phosphatidylinositol-mediated signaling // positive regulation of protein kinase B signaling // cellular response to cAMP // regulation of calcium ion transmembrane transport // negative regulation of fibroblast apoptotic process | Cluster0018 |
| RGS4 | 14402028 | PREDICTED: Canis lupus familiaris regulator of G-protein signaling 4 (RGS4), transcript variant X2, mRNA. | --- | Cluster0018 |
| SHC3 | 14257755 | PREDICTED: Canis lupus familiaris SHC (Src homology 2 domain containing) transforming protein 3 (SHC3), mRNA. | --- | Cluster0018 |
| SPI1 | 14311483 | PREDICTED: Canis lupus familiaris Spi-1 proto-oncogene (SPI1), transcript variant X1, mRNA. | --- | Cluster0018 |
| TFPI2 | 14292597 | PREDICTED: Canis lupus familiaris tissue factor pathway inhibitor 2 (TFPI2), mRNA. | negative regulation of endopeptidase activity | Cluster0018 |
| TIMELESS | 14270105 | PREDICTED: Canis lupus familiaris timeless circadian clock (TIMELESS), mRNA. | --- | Cluster0018 |
| TJP2 | 14263041 | Canis lupus familiaris tight junction protein 2 (TJP2), mRNA. | --- | Cluster0018 |
| TNFSF8 | 14278977 | PREDICTED: Canis lupus familiaris tumor necrosis factor (ligand) superfamily, member 8 (TNFSF8), mRNA. | immune response // CD8-positive, alpha-beta T cell differentiation // positive regulation of transcription from RNA polymerase II promoter // defense response to Gram-positive bacterium | Cluster0018 |
| TVP23A | 14421876 | PREDICTED: Canis lupus familiaris trans-golgi network vesicle protein 23 homolog A (S. cerevisiae) (TVP23A), mRNA. | --- | Cluster0018 |
| VAV1 | 14336541 | PREDICTED: Canis lupus familiaris vav 1 guanine nucleotide exchange factor (VAV1), transcript variant X2, mRNA. | --- | Cluster0018 |

| | | | | |
|---|---|---|---|---|
| --- | 14339560 | Uncharacterized protein [gene_biotype:protein_coding transcript_biotype:protein_coding] | --- | Cluster0021 |
| --- | 14467610 | PREDICTED: Canis lupus familiaris HECT domain and ankyrin repeat containing E3 ubiquitin protein ligase 1 (HACE1), transcript variant X2, mRNA. | --- | Cluster0021 |
| --- | 14467810 | Canis lupus familiaris peroxiredoxin 1 (PRDX1), mRNA. | --- | Cluster0021 |
| --- | 14469444 | PREDICTED: Canis lupus familiaris HIG1 hypoxia inducible domain family, member 1A (HIGD1A), transcript variant X1, mRNA. | --- | Cluster0021 |
| --- | 14469446 | PREDICTED: Canis lupus familiaris HIG1 hypoxia inducible domain family, member 1A (HIGD1A), transcript variant X2, mRNA. | --- | Cluster0021 |
| --- | 14470374 | Canis lupus familiaris peroxiredoxin 1 (PRDX1), mRNA. | --- | Cluster0021 |
| ANXA1 | 14262925 | Canis lupus familiaris annexin A1 (ANXA1), mRNA. | neutrophil homeostasis // signal transduction // myoblast migration involved in skeletal muscle regeneration // peptide cross-linking // keratinocyte differentiation // positive regulation of vesicle fusion // positive regulation of neutrophil apoptotic process // regulation of cell proliferation // negative regulation of catalytic activity // alpha-beta T cell differentiation // arachidonic acid secretion // cellular response to glucocorticoid stimulus // neutrophil clearance // positive regulation of G1/S transition of mitotic cell cycle // negative regulation of interleukin-8 secretion | Cluster0021 |
| CCDC91 | 14366173 | PREDICTED: Canis lupus familiaris coiled-coil domain containing 91 (CCDC91), transcript variant X2, mRNA. | --- | Cluster0021 |
| CLEC7A | 14364312 | PREDICTED: Canis lupus familiaris C-type lectin domain family 7, member A (CLEC7A), transcript variant X2, mRNA. | --- | Cluster0021 |
| CMPK1 | 14297170 | PREDICTED: Canis lupus familiaris cytidine monophosphate (UMP-CMP) kinase 1, cytosolic (CMPK1), mRNA. | nucleobase-containing compound metabolic process // nucleoside diphosphate phosphorylation // de novo pyrimidine nucleobase biosynthetic process // pyrimidine nucleotide biosynthetic process // nucleoside triphosphate biosynthetic process // phosphorylation | Cluster0021 |

| | | | | |
|---|---|---|---|---|
| COMMD8 | 14289182 | PREDICTED: Canis lupus familiaris COMM domain containing 8 (COMMD8), mRNA. | --- | Cluster0021 |
| DNAJA1 | 14275171 | Canis lupus familiaris DnaJ (Hsp40) homolog, subfamily A, member 1 (DNAJA1), mRNA. | protein folding // response to heat | Cluster0021 |
| DPH5 | 14423221 | PREDICTED: Canis lupus familiaris diphthamide biosynthesis 5 (DPH5), transcript variant X1, mRNA. | --- | Cluster0021 |
| HSPA13 | 14385497 | PREDICTED: Canis lupus familiaris heat shock protein 70kDa family, member 13 (HSPA13), mRNA. | --- | Cluster0021 |
| IQGAP2 | 14377012 | PREDICTED: Canis lupus familiaris IQ motif containing GTPase activating protein 2 (IQGAP2), transcript variant X1, mRNA. | signal transduction // small GTPase mediated signal transduction // Arp2/3 complex-mediated actin nucleation // regulation of GTPase activity // positive regulation of GTPase activity // thrombin receptor signaling pathway | Cluster0021 |
| LYPLA1 | 14372923 | PREDICTED: Canis lupus familiaris lysophospholipase I (LYPLA1), mRNA. | --- | Cluster0021 |
| PRDX1 | 14294722 | Canis lupus familiaris peroxiredoxin 1 (PRDX1), mRNA. | oxidation-reduction process | Cluster0021 |
| SERPINI1 | 14392236 | PREDICTED: Canis lupus familiaris serpin peptidase inhibitor, clade I (neuroserpin), member 1 (SERPINI1), transcript variant X1, mRNA. | --- | Cluster0021 |
| ZEB1 | 14323581 | PREDICTED: Canis lupus familiaris zinc finger E-box binding homeobox 1 (ZEB1), transcript variant X1, mRNA. | negative regulation of transcription from RNA polymerase II promoter // pattern specification process // central nervous system development // negative regulation of cell proliferation // regulation of mesenchymal cell proliferation // regulation of transforming growth factor beta receptor signaling pathway // negative regulation of epithelial cell differentiation // regulation of T cell differentiation in thymus // positive regulation of neuron differentiation // negative regulation of transcription, DNA-templated // positive regulation of transcription from RNA polymerase II promoter // organ development // embryonic camera-type eye morphogenesis // embryonic morphogenesis // embryonic skeletal system morphogenesis // semicircular canal morphogenesis // regulation of smooth muscle cell differentiation // cartilage development // cellular response to amino acid stimulus // cochlea morphogenesis | Cluster0021 |

| | | | | |
|---|---|---|---|---|
| ANLN | 14291482 | PREDICTED: Canis lupus familiaris anillin actin binding protein (ANLN), transcript variant X2, mRNA. | --- | Cluster0026 |
| CCNA2 | 14317057 | PREDICTED: Canis lupus familiaris cyclin A2 (CCNA2), mRNA. | regulation of cyclin-dependent protein serine/threonine kinase activity // Ras protein signal transduction // regulation of G2/M transition of mitotic cell cycle // positive regulation of transcription, DNA-templated | Cluster0026 |
| CENPF | 14429784 | PREDICTED: Canis lupus familiaris centromere protein F, 350/400kDa (CENPF), mRNA. | --- | Cluster0026 |
| CEP55 | 14367893 | PREDICTED: Canis lupus familiaris centrosomal protein 55kDa (CEP55), mRNA. | --- | Cluster0026 |
| DTL | 14429668 | PREDICTED: Canis lupus familiaris denticleless E3 ubiquitin protein ligase homolog (Drosophila) (DTL), transcript variant X1, mRNA. | --- | Cluster0026 |
| ESCO2 | 14355933 | PREDICTED: Canis lupus familiaris establishment of sister chromatid cohesion N-acetyltransferase 2 (ESCO2), mRNA. | --- | Cluster0026 |
| KIF11 | 14367827 | PREDICTED: Canis lupus familiaris kinesin family member 11 (KIF11), mRNA. | --- | Cluster0026 |
| KIF15 | 14334859 | PREDICTED: Canis lupus familiaris kinesin family member 15 (KIF15), transcript variant X1, mRNA. | --- | Cluster0026 |
| KPNA2 | 14450590 | PREDICTED: Canis lupus familiaris karyopherin alpha 2 (RAG cohort 1, importin alpha 1) (KPNA2), transcript variant X2, mRNA. | protein import into nucleus // NLS-bearing protein import into nucleus // transport // protein transport // cytokine-mediated signaling pathway | Cluster0026 |
| MASTL | 14319201 | PREDICTED: Canis lupus familiaris microtubule associated serine/threonine kinase-like (MASTL), transcript variant X3, mRNA. | --- | Cluster0026 |
| MCM10 | 14323823 | PREDICTED: Canis lupus familiaris minichromosome maintenance 10 replication initiation factor (MCM10), transcript variant X2, mRNA. | DNA replication // DNA replication initiation // cell proliferation | Cluster0026 |
| NAMPT | 14310283 | PREDICTED: Canis lupus familiaris nicotinamide phosphoribosyltransferase (NAMPT), mRNA. | --- | Cluster0026 |
| NCAPG2 | 14302182 | PREDICTED: Canis lupus familiaris non-SMC condensin II complex, subunit G2 (NCAPG2), transcript variant X2, mRNA. | --- | Cluster0026 |

| | | | | |
|---|---|---|---|---|
| NDC80 | 14432674 | PREDICTED: Canis lupus familiaris NDC80 kinetochore complex component (NDC80), mRNA. | establishment of mitotic spindle orientation // establishment of mitotic spindle orientation // mitotic spindle organization // mitotic spindle organization // chromosome segregation // attachment of spindle microtubules to kinetochore // attachment of mitotic spindle microtubules to kinetochore | Cluster0026 |
| NDUFB2 | 14301602 | PREDICTED: Canis lupus familiaris NADH dehydrogenase (ubiquinone) 1 beta subcomplex, 2, 8kDa (NDUFB2), mRNA. | --- | Cluster0026 |
| PHTF2 | 14310545 | PREDICTED: Canis lupus familiaris putative homeodomain transcription factor 2 (PHTF2), transcript variant X1, mRNA. | --- | Cluster0026 |
| TOP2A | 14445750 | PREDICTED: Canis lupus familiaris topoisomerase (DNA) II alpha (TOP2A), transcript variant X1, mRNA. | resolution of meiotic recombination intermediates // sister chromatid segregation // DNA metabolic process // DNA topological change // DNA topological change // DNA unwinding involved in DNA replication // mitotic recombination // apoptotic chromosome condensation // mitotic DNA integrity checkpoint | Cluster0026 |
| --- | 14260285 | PREDICTED: Canis lupus familiaris zinc finger protein 790 (ZNF790), transcript variant X1, mRNA. | --- | Cluster0030 |
| --- | 14418124 | Small nucleolar RNA MBII-202 [gene_biotype:snoRNA transcript_biotype:snoRNA] | --- | Cluster0030 |
| --- | 14469142 | lamin B2 [gene_biotype:protein_coding transcript_biotype:protein_coding] | --- | Cluster0030 |
| --- | 14476827 | --- | --- | Cluster0030 |
| --- | 14481959 | --- | --- | Cluster0030 |
| DRAP1 | 14315880 | PREDICTED: Canis lupus familiaris DR1-associated protein 1 (negative cofactor 2 alpha) (DRAP1), mRNA. | --- | Cluster0030 |
| H2AFJ | 14366569 | PREDICTED: Canis lupus familiaris H2A histone family, member J (H2AFJ), mRNA. | chromatin organization // chromatin silencing | Cluster0030 |
| MED19 | 14311129 | PREDICTED: Canis lupus familiaris mediator complex subunit 19 (MED19), mRNA. | --- | Cluster0030 |
| NDUFB4 | 14389485 | PREDICTED: Canis lupus familiaris NADH dehydrogenase (ubiquinone) 1 beta subcomplex, 4, 15kDa (NDUFB4), mRNA. | response to oxidative stress // oxidation-reduction process | Cluster0030 |

| | | | | |
|---|---|---|---|---|
| PRADC1 | 14305581 | PREDICTED: Canis lupus familiaris protease-associated domain containing 1 (PRADC1), transcript variant X1, mRNA. | --- | Cluster0030 |
| RABAC1 | 14259815 | Canis lupus familiaris Rab acceptor 1 (prenylated) (RABAC1), mRNA. | --- | Cluster0030 |
| RPP25L | 14277903 | PREDICTED: Canis lupus familiaris ribonuclease P/MRP 25kDa subunit-like (RPP25L), transcript variant X2, mRNA. | --- | Cluster0030 |
| S100A6 | 14432045 | PREDICTED: Canis lupus familiaris S100 calcium binding protein A6 (S100A6), mRNA. | --- | Cluster0030 |
| SDAD1 | 14387630 | PREDICTED: Canis lupus familiaris SDA1 domain containing 1 (SDAD1), mRNA. | --- | Cluster0030 |
| SERPINC1 | 14430500 | PREDICTED: Canis lupus familiaris serpin peptidase inhibitor, clade C (antithrombin), member 1 (SERPINC1), mRNA. | negative regulation of endopeptidase activity // regulation of blood coagulation, intrinsic pathway | Cluster0030 |
| YIPF1 | 14411749 | PREDICTED: Canis lupus familiaris Yip1 domain family, member 1 (YIPF1), transcript variant X1, mRNA. | positive regulation of defense response to virus by host // mitophagy in response to mitochondrial depolarization | Cluster0030 |
| --- | 14410481 | CD68 molecule [gene_biotype:protein_coding transcript_biotype:protein_coding] | --- | Cluster0034 |
| --- | 14466558 | PREDICTED: Canis lupus familiaris legumain (LGMN), mRNA. | --- | Cluster0034 |
| AKIP1 | 14338842 | PREDICTED: Canis lupus familiaris A kinase (PRKA) interacting protein 1 (AKIP1), transcript variant X1, mRNA. | --- | Cluster0034 |
| BIN2 | 14362703 | PREDICTED: Canis lupus familiaris bridging integrator 2 (BIN2), transcript variant X1, mRNA. | --- | Cluster0034 |
| BTBD6 | 14440050 | PREDICTED: Canis lupus familiaris BTB (POZ) domain containing 6 (BTBD6), mRNA. | --- | Cluster0034 |
| CCL7 | 14452725 | Canis lupus familiaris chemokine (C-C motif) ligand 7 (CCL7), mRNA. | chemotaxis // immune response // cell chemotaxis | Cluster0034 |
| CNTNAP4 | 14418590 | PREDICTED: Canis lupus familiaris contactin associated protein-like 4 (CNTNAP4), mRNA. | regulation of synaptic transmission, dopaminergic // regulation of synaptic transmission, GABAergic // regulation of grooming behavior | Cluster0034 |
| IGFBP2 | 14398628 | PREDICTED: Canis lupus familiaris insulin-like growth factor binding protein 2, 36kDa (IGFBP2), partial mRNA. | --- | Cluster0034 |

| | | | | |
|---|---|---|---|---|
| IL10RB | 14384661 | PREDICTED: Canis lupus familiaris interleukin 10 receptor, beta (IL10RB), transcript variant X1, mRNA. | blood coagulation // cytokine-mediated signaling pathway | Cluster0034 |
| ITGA10 | 14309269 | PREDICTED: Canis lupus familiaris integrin, alpha 10 (ITGA10), transcript variant X1, mRNA. | --- | Cluster0034 |
| RGS16 | 14433802 | PREDICTED: Canis lupus familiaris regulator of G-protein signaling 16 (RGS16), transcript variant X1, mRNA. | positive regulation of GTPase activity | Cluster0034 |
| SLC11A1 | 14398682 | Canis lupus familiaris solute carrier family 11 (proton-coupled divalent metal ion transporters), member 1 (SLC11A1), mRNA. | manganese ion transport // iron ion homeostasis // manganese ion transmembrane transport // transport // ion transport | Cluster0034 |
| SLCO2A1 | 14346804 | Canis lupus familiaris solute carrier organic anion transporter family, member 2A1 (SLCO2A1), mRNA. | transport // ion transport | Cluster0034 |
| TMEM86A | 14339283 | PREDICTED: Canis lupus familiaris transmembrane protein 86A (TMEM86A), transcript variant X1, mRNA. | --- | Cluster0034 |
| TNFRSF11B | 14287959 | PREDICTED: Canis lupus familiaris tumor necrosis factor receptor superfamily, member 11b (TNFRSF11B), mRNA. | apoptotic process // inflammatory response // immune response // signal transduction // multicellular organismal development // extracellular matrix organization // response to lipopolysaccharide // tumor necrosis factor-mediated signaling pathway // regulation of cell proliferation // negative regulation of odontogenesis of dentin-containing tooth // regulation of apoptotic process // positive regulation of MAPK cascade // apoptotic signaling pathway | Cluster0034 |
| --- | 14400854 | U6 spliceosomal RNA [gene_biotype:snRNA transcript_biotype:snRNA] | --- | Cluster0042 |
| --- | 14464308 | family with sequence similarity 58, member A [gene_biotype:protein_coding transcript_biotype:protein_coding] | regulation of cyclin-dependent protein serine/threonine kinase activity // regulation of transcription, DNA-templated // activation of protein kinase activity // positive regulation of MAPK cascade // positive regulation of cyclin-dependent protein serine/threonine kinase activity // positive regulation of transcription from RNA polymerase II promoter // positive regulation of phosphorylation of RNA polymerase II C-terminal domain | Cluster0042 |
| --- | 14465384 | PREDICTED: Canis lupus familiaris ubiquitin specific peptidase 48 (USP48), transcript variant X1, mRNA. | --- | Cluster0042 |

| | | | | |
|---|---|---|---|---|
| CLIC1 | 14282497 | Canis lupus familiaris chloride intracellular channel 1 (CLIC1), mRNA. | transport // ion transport // chloride transport // regulation of ion transmembrane transport // regulation of cell cycle // chloride transmembrane transport | Cluster0042 |
| CREB3 | 14275399 | PREDICTED: Canis lupus familiaris cAMP responsive element binding protein 3 (CREB3), mRNA. | --- | Cluster0042 |
| CSRP2 | 14298017 | PREDICTED: Canis lupus familiaris cysteine and glycine-rich protein 2 (CSRP2), mRNA. | --- | Cluster0042 |
| IK | 14320143 | PREDICTED: Canis lupus familiaris IK cytokine, down-regulator of HLA II (IK), mRNA. | RNA metabolic process // ribosomal large subunit biogenesis | Cluster0042 |
| JAK2 | 14257638 | PREDICTED: Canis lupus familiaris Janus kinase 2 (JAK2), transcript variant X1, mRNA. | --- | Cluster0042 |
| MED10 | 14391298 | PREDICTED: Canis lupus familiaris mediator complex subunit 10 (MED10), mRNA. | --- | Cluster0042 |
| RTCA | 14428205 | PREDICTED: Canis lupus familiaris RNA 3-terminal phosphate cyclase (RTCA), transcript variant X1, mRNA. | --- | Cluster0042 |
| SEC61B | 14275773 | Canis lupus familiaris Sec61 translocon beta subunit (SEC61B), mRNA. | protein import into nucleus, translocation // ER-associated ubiquitin-dependent protein catabolic process // retrograde protein transport, ER to cytosol // protein transmembrane transport // transport // intracellular protein transport // protein transport | Cluster0042 |
| SMDT1 | 14271261 | PREDICTED: Canis lupus familiaris single-pass membrane protein with aspartate-rich tail 1 (SMDT1), mRNA. | mitochondrial calcium ion transport // calcium ion transmembrane import into mitochondrion // mitochondrial calcium ion homeostasis | Cluster0042 |
| SSR4 | 14460782 | Canis lupus familiaris signal sequence receptor, delta (SSR4), mRNA. | --- | Cluster0042 |
| USO1 | 14386466 | PREDICTED: Canis lupus familiaris USO1 vesicle transport factor (USO1), transcript variant X1, mRNA. | --- | Cluster0042 |
| --- | 14472398 | PREDICTED: Canis lupus familiaris collagen, type VI, alpha 5 (COL6A5), transcript variant X3, mRNA. | --- | Cluster0056 |
| --- | 14472400 | PREDICTED: Canis lupus familiaris collagen, type VI, alpha 5 (COL6A5), transcript variant X3, mRNA. | --- | Cluster0056 |

| | | | | |
|---|---|---|---|---|
| --- | 14473206 | PREDICTED: Canis lupus familiaris collagen, type VI, alpha 5 (COL6A5), transcript variant X3, mRNA. | --- | Cluster0056 |
| --- | 14473208 | PREDICTED: Canis lupus familiaris collagen, type VI, alpha 5 (COL6A5), transcript variant X3, mRNA. | --- | Cluster0056 |
| --- | 14474170 | PREDICTED: Canis lupus familiaris collagen, type VI, alpha 5 (COL6A5), transcript variant X3, mRNA. | --- | Cluster0056 |
| --- | 14474172 | PREDICTED: Canis lupus familiaris collagen, type VI, alpha 5 (COL6A5), transcript variant X3, mRNA. | --- | Cluster0056 |
| COL6A5 | 14345036 | PREDICTED: Canis lupus familiaris collagen, type VI, alpha 5 (COL6A5), transcript variant X3, mRNA. | --- | Cluster0056 |
| GREM1 | 14381696 | PREDICTED: Canis lupus familiaris gremlin 1, DAN family BMP antagonist (GREM1), transcript variant X2, mRNA. | cell morphogenesis // angiogenesis // branching involved in ureteric bud morphogenesis // cell migration involved in sprouting angiogenesis // positive regulation of receptor internalization // positive regulation of transcription from RNA polymerase II promoter involved in myocardial precursor cell differentiation // mesenchymal to epithelial transition involved in metanephros morphogenesis // signal transduction // activation of transmembrane receptor protein tyrosine kinase activity // cell-cell signaling // positive regulation of cell proliferation // organ morphogenesis // proximal/distal pattern formation // regulation of epithelial to mesenchymal transition // collagen fibril organization // embryonic limb morphogenesis // negative regulation of bone mineralization // negative regulation of BMP signaling pathway // negative regulation of chondrocyte differentiation // negative regulation of osteoblast proliferation // positive regulation of NF-kappaB import into nucleus // negative regulation of apoptotic process // endothelial cell migration // positive regulation of angiogenesis // negative regulation of transcription, DNA-templated // positive regulation of transcription, DNA-templated // positive regulation of transcription from RNA polymerase II promoter // negative regulation of bone remodeling // determination of dorsal identity // positive regulation of NF-kappaB transcription | Cluster0056 |

| | | | | |
|---|---|---|---|---|
| | | | factor activity // regulation of focal adhesion assembly // positive regulation of telomerase activity // limb development // negative regulation of pathway-restricted SMAD protein phosphorylation // ureteric bud formation // positive regulation of protein tyrosine kinase activity // negative regulation of canonical Wnt signaling pathway // positive regulation of branching involved in ureteric bud morphogenesis // negative regulation of branching involved in ureteric bud morphogenesis // negative regulation of osteoclast proliferation // positive regulation of peptidyl-tyrosine autophosphorylation // negative regulation of bone trabecula formation // negative regulation of bone mineralization involved in bone maturation // positive regulation of receptor activity // positive regulation of cardiac muscle cell differentiation | |
| HTR4 | 14404762 | PREDICTED: Canis lupus familiaris 5-hydroxytryptamine (serotonin) receptor 4, G protein-coupled (HTR4), transcript variant X2, mRNA. | --- | Cluster0056 |
| LOC484306 | 14263810 | PREDICTED: Canis lupus familiaris leukocyte immunoglobulin-like receptor subfamily B member 3 (LOC484306), transcript variant X1, mRNA. | --- | Cluster0056 |
| MICAL1 | 14285294 | PREDICTED: Canis lupus familiaris microtubule associated monooxygenase, calponin and LIM domain containing 1 (MICAL1), mRNA. | --- | Cluster0056 |
| SOD2 | 14262002 | PREDICTED: Canis lupus familiaris superoxide dismutase 2, mitochondrial (SOD2), mRNA. | response to reactive oxygen species // age-dependent response to oxidative stress // release of cytochrome c from mitochondria // liver development // detection of oxygen // vasodilation by acetylcholine involved in regulation of systemic arterial blood pressure // glutathione metabolic process // superoxide metabolic process // response to oxidative stress // mitochondrion organization // heart development // locomotory behavior // regulation of blood pressure // intrinsic apoptotic signaling pathway in response to DNA damage // intrinsic apoptotic signaling pathway in response to oxidative stress // apoptotic mitochondrial changes // post-embryonic development // response to gamma radiation // response to activity // removal of superoxide radicals // respiratory electron | Cluster0056 |

| | | | | |
|---|---|---|---|---|
| | | | transport chain // hemopoiesis // vasodilation // response to hydrogen peroxide // superoxide anion generation // positive regulation of nitric oxide biosynthetic process // negative regulation of fat cell differentiation // negative regulation of fibroblast proliferation // neuron development // response to axon injury // erythrophore differentiation // regulation of catalytic activity // regulation of mitochondrial membrane potential // iron ion homeostasis // response to hyperoxia // oxidation-reduction process | |
| ATOX1 | 14404489 | Canis lupus familiaris antioxidant 1 copper chaperone (ATOX1), mRNA. | cellular copper ion homeostasis // response to oxidative stress // intracellular copper ion transport // transport // ion transport // copper ion transport // cellular copper ion homeostasis // cellular copper ion homeostasis // response to oxidative stress // intracellular copper ion transport // metal ion transport | Cluster0065 |
| ATXN3 | 14440216 | PREDICTED: Canis lupus familiaris ataxin 3 (ATXN3), transcript variant X2, mRNA. | --- | Cluster0065 |
| CKAP2L | 14308165 | PREDICTED: Canis lupus familiaris cytoskeleton associated protein 2-like (CKAP2L), transcript variant X1, mRNA. | --- | Cluster0065 |
| GNPTAB | 14298561 | PREDICTED: Canis lupus familiaris N-acetylglucosamine-1-phosphate transferase, alpha and beta subunits (GNPTAB), mRNA. | --- | Cluster0065 |
| NOP10 | 14379480 | PREDICTED: Canis lupus familiaris NOP10 ribonucleoprotein (NOP10), mRNA. | pseudouridine synthesis // ribosome biogenesis | Cluster0065 |
| P4HA1 | 14406456 | PREDICTED: Canis lupus familiaris prolyl 4-hydroxylase, alpha polypeptide I (P4HA1), transcript variant X3, mRNA. | peptidyl-proline hydroxylation // oxidation-reduction process | Cluster0065 |
| RPS6KC1 | 14429748 | PREDICTED: Canis lupus familiaris ribosomal protein S6 kinase, 52kDa, polypeptide 1 (RPS6KC1), transcript variant X1, mRNA. | protein phosphorylation // signal transduction | Cluster0065 |
| SEC31A | 14387849 | PREDICTED: Canis lupus familiaris SEC31 homolog A, COPII coat complex component (SEC31A), transcript variant X1, mRNA. | --- | Cluster0065 |
| SNX14 | 14284664 | PREDICTED: Canis lupus familiaris sorting nexin 14 (SNX14), transcript variant X1, mRNA. | --- | Cluster0065 |
| THOC7 | 14328071 | PREDICTED: Canis lupus familiaris THO complex 7 (THOC7), transcript variant X1, mRNA. | --- | Cluster0065 |

| | | | | |
|---|---|---|---|---|
| --- | 14467368 | PREDICTED: Canis lupus familiaris tropomyosin 2 (beta) (TPM2), transcript variant X4, mRNA. | --- | Cluster0071 |
| --- | 14467372 | PREDICTED: Canis lupus familiaris tropomyosin 2 (beta) (TPM2), transcript variant X4, mRNA. | --- | Cluster0071 |
| ACTA2 | 14362290 | PREDICTED: Canis lupus familiaris actin, alpha 2, smooth muscle, aorta (ACTA2), mRNA. | regulation of blood pressure // response to virus // positive regulation of gene expression // vascular smooth muscle contraction // glomerular mesangial cell development // mesenchyme migration | Cluster0071 |
| CNN1 | 14335896 | PREDICTED: Canis lupus familiaris calponin 1, basic, smooth muscle (CNN1), transcript variant X1, mRNA. | --- | Cluster0071 |
| ITGA1 | 14408061 | PREDICTED: Canis lupus familiaris integrin, alpha 1 (ITGA1), mRNA. | cell adhesion // cell-matrix adhesion // integrin-mediated signaling pathway // negative regulation of cell proliferation // neutrophil chemotaxis // positive regulation of phosphoprotein phosphatase activity // negative regulation of epidermal growth factor receptor signaling pathway // cellular extravasation | Cluster0071 |
| LPP | 14392051 | PREDICTED: Canis lupus familiaris LIM domain containing preferred translocation partner in lipoma (LPP), transcript variant X2, mRNA. | --- | Cluster0071 |
| MRVI1 | 14341088 | PREDICTED: Canis lupus familiaris murine retrovirus integration site 1 homolog (MRVI1), transcript variant X3, mRNA. | --- | Cluster0071 |
| NPNT | 14387263 | PREDICTED: Canis lupus familiaris nephronectin (NPNT), transcript variant X1, mRNA. | --- | Cluster0071 |
| TAGLN | 14414808 | PREDICTED: Canis lupus familiaris transgelin (TAGLN), mRNA. | --- | Cluster0071 |
| TPM2 | 14278070 | PREDICTED: Canis lupus familiaris tropomyosin 2 (beta) (TPM2), transcript variant X4, mRNA. | --- | Cluster0071 |
| --- | 14465776 | PREDICTED: Canis lupus familiaris zinc finger, BED-type containing 8 (ZBED8), mRNA. | --- | Cluster0079 |
| DYNLL1 | 14358181 | PREDICTED: Canis lupus familiaris dynein, light chain, LC8-type 1 (DYNLL1), transcript variant X1, mRNA. | microtubule-based process // substantia nigra development // intraciliary retrograde transport // negative regulation of phosphorylation // cilium morphogenesis | Cluster0079 |
| EMC6 | 14447865 | PREDICTED: Canis lupus familiaris ER membrane protein complex subunit 6 (EMC6), mRNA. | autophagosome assembly // autophagosome assembly // protein folding in endoplasmic reticulum | Cluster0079 |
| HACE1 | 14285126 | PREDICTED: Canis lupus familiaris HECT domain and ankyrin repeat containing E3 ubiquitin | --- | Cluster0079 |

| | | | | |
|---|---|---|---|---|
| | | protein ligase 1 (HACE1), transcript variant X2, mRNA. | | |
| NAA20 | 14350394 | PREDICTED: Canis lupus familiaris N(alpha)-acetyltransferase 20, NatB catalytic subunit (NAA20), transcript variant X2, mRNA. | --- | Cluster0079 |
| NMNAT1 | 14412423 | PREDICTED: Canis lupus familiaris nicotinamide nucleotide adenylyltransferase 1 (NMNAT1), transcript variant X1, mRNA. | water-soluble vitamin metabolic process // biosynthetic process // NAD biosynthetic process // pyridine nucleotide biosynthetic process // de novo NAD biosynthetic process from aspartate | Cluster0079 |
| SSX2IP | 14423785 | PREDICTED: Canis lupus familiaris synovial sarcoma, X breakpoint 2 interacting protein (SSX2IP), transcript variant X1, mRNA. | --- | Cluster0079 |
| TMEM261 | 14277308 | PREDICTED: Canis lupus familiaris transmembrane protein 261 (TMEM261), transcript variant X2, mRNA. | --- | Cluster0079 |
| UAP1 | 14402061 | PREDICTED: Canis lupus familiaris UDP-N-acetylglucosamine pyrophosphorylase 1 (UAP1), transcript variant X1, mRNA. | --- | Cluster0079 |
| --- | 14472376 | Canis lupus familiaris hemoglobin subunit beta-like (LOC609402), mRNA. | --- | Cluster0080 |
| --- | 14472940 | Canis lupus familiaris hemoglobin subunit beta-like (LOC609402), mRNA. | --- | Cluster0080 |
| --- | 14474370 | Canis lupus familiaris hemoglobin subunit beta-like (LOC609402), mRNA. | --- | Cluster0080 |
| ALAS2 | 14462550 | PREDICTED: Canis lupus familiaris 5-aminolevulinate synthase 2 (ALAS2), mRNA. | --- | Cluster0080 |
| ATP6V1B2 | 14356438 | PREDICTED: Canis lupus familiaris ATPase, H+ transporting, lysosomal 56/58kDa, V1 subunit B2 (ATP6V1B2), mRNA. | transport // ion transport // ATP hydrolysis coupled proton transport // proton transport // ATP metabolic process | Cluster0080 |
| HPSE | 14387927 | heparanase [gene_biotype:protein_coding transcript_biotype:protein_coding] | carbohydrate metabolic process // cell-matrix adhesion // positive regulation of vascular endothelial growth factor production // positive regulation of blood coagulation // heparan sulfate proteoglycan catabolic process // positive regulation of osteoblast proliferation // wound healing // regulation of hair follicle development // positive regulation of hair follicle development // positive regulation of protein kinase B signaling // angiogenesis involved in wound healing // vascular wound healing | Cluster0080 |

| | | | | |
|---|---|---|---|---|
| SPIRE1 | 14436539 | PREDICTED: Canis lupus familiaris spire-type actin nucleation factor 1 (SPIRE1), mRNA. | --- | Cluster0080 |
| SUGCT | 14313394 | PREDICTED: Canis lupus familiaris succinyl-CoA:glutarate-CoA transferase (SUGCT), transcript variant X2, mRNA. | --- | Cluster0080 |
| TMEM251 | 14439109 | PREDICTED: Canis lupus familiaris transmembrane protein 251 (TMEM251), mRNA. | --- | Cluster0080 |
| ABCC4 | 14343463 | Canis lupus familiaris ATP-binding cassette, sub-family C (CFTR/MRP), member 4 (ABCC4), mRNA. | transport // metabolic process // transmembrane transport | Cluster0086 |
| BBX | 14389121 | PREDICTED: Canis lupus familiaris bobby sox homolog (Drosophila) (BBX), transcript variant X1, mRNA. | bone development | Cluster0086 |
| CCL2 | 14452728 | Canis lupus familiaris chemokine (C-C motif) ligand 2 (CCL2), mRNA. | inflammatory response // immune response // cell chemotaxis // chemotaxis | Cluster0086 |
| DBNDD2 | 14349404 | PREDICTED: Canis lupus familiaris dysbindin (dystrobrevin binding protein 1) domain containing 2 (DBNDD2), transcript variant X2, mRNA. | negative regulation of protein kinase activity | Cluster0086 |
| GPX1 | 14329003 | Canis lupus familiaris glutathione peroxidase 1 (GPX1), mRNA. | response to oxidative stress // oxidation-reduction process | Cluster0086 |
| LOC100856638 | 14456695 | PREDICTED: Canis lupus familiaris uridine phosphorylase 1-like (LOC100856638), transcript variant X2, mRNA. | --- | Cluster0086 |
| SLC38A5 | 14462027 | PREDICTED: Canis lupus familiaris solute carrier family 38, member 5 (SLC38A5), transcript variant X1, mRNA. | --- | Cluster0086 |
| VCAM1 | 14428176 | Canis lupus familiaris vascular cell adhesion molecule 1 (VCAM1), mRNA. | single organismal cell-cell adhesion // cell adhesion | Cluster0086 |
| WDR1 | 14375792 | Canis lupus familiaris WD repeat domain 1 (WDR1), mRNA. | actin cytoskeleton organization // positive regulation of actin filament depolymerization // sarcomere organization // regulation of ventricular cardiac muscle cell membrane repolarization | Cluster0086 |
| BPNT1 | 14401876 | PREDICTED: Canis lupus familiaris 3(2), 5-bisphosphate nucleotidase 1 (BPNT1), transcript variant X1, mRNA. | --- | Cluster0117 |

| | | | | |
|---|---|---|---|---|
| CAMK1 | 14327689 | PREDICTED: Canis lupus familiaris calcium/calmodulin-dependent protein kinase I (CAMK1), mRNA. | --- | Cluster0117 |
| CIB1 | 14377820 | PREDICTED: Canis lupus familiaris calcium and integrin binding 1 (calmyrin) (CIB1), transcript variant X2, mRNA. | --- | Cluster0117 |
| CRIP1 | 14440103 | PREDICTED: Canis lupus familiaris cysteine-rich protein 1 (intestinal) (CRIP1), mRNA. | --- | Cluster0117 |
| FAM188A | 14319591 | PREDICTED: Canis lupus familiaris family with sequence similarity 188, member A (FAM188A), mRNA. | --- | Cluster0117 |
| FAM210A | 14255450 | PREDICTED: Canis lupus familiaris family with sequence similarity 210, member A (FAM210A), mRNA. | --- | Cluster0117 |
| PSMD8 | 14265672 | PREDICTED: Canis lupus familiaris proteasome 26S subunit, non-ATPase 8 (PSMD8), mRNA. | proteolysis // regulation of protein stability // proteasome-mediated ubiquitin-dependent protein catabolic process // proteasome assembly // positive regulation of protein targeting to mitochondrion | Cluster0117 |
| --- | 14306389 | regulatory subunit of type II PKA R-subunit (RIIa) domain containing 1 [gene_biotype:protein_coding transcript_biotype:protein_coding] | --- | Cluster0122 |
| DYNC1LI1 | 14344657 | PREDICTED: Canis lupus familiaris dynein, cytoplasmic 1, light intermediate chain 1 (DYNC1LI1), mRNA. | --- | Cluster0122 |
| EPRS | 14401844 | PREDICTED: Canis lupus familiaris glutamyl-prolyl-tRNA synthetase (EPRS), transcript variant X1, mRNA. | --- | Cluster0122 |
| FAM149B1 | 14403451 | PREDICTED: Canis lupus familiaris family with sequence similarity 149, member B1 (FAM149B1), transcript variant X2, mRNA. | --- | Cluster0122 |
| TMEM263 | 14272013 | PREDICTED: Canis lupus familiaris transmembrane protein 263 (TMEM263), transcript variant X1, mRNA. | --- | Cluster0122 |
| TTC37 | 14374004 | PREDICTED: Canis lupus familiaris tetratricopeptide repeat domain 37 (TTC37), transcript variant X4, mRNA. | --- | Cluster0122 |

| | | | | |
|---|---|---|---|---|
| YIPF5 | 14324455 | PREDICTED: Canis lupus familiaris Yip1 domain family, member 5 (YIPF5), transcript variant X2, mRNA. | --- | Cluster0122 |
| --- | 14388993 | cdna:genscan chromosome:CanFam3.1:33:6868897:6958980:1 transcript_biotype:protein_coding | --- | Cluster0140 |
| --- | 14478451 | --- | --- | Cluster0140 |
| --- | 14481461 | --- | --- | Cluster0140 |
| CLN5 | 14342163 | Canis lupus familiaris ceroid-lipofuscinosis, neuronal 5 (CLN5), mRNA. | signal peptide processing // lysosomal lumen acidification // brain development // visual perception // neurogenesis // glycosylation // lysosome organization // neurogenesis | Cluster0140 |
| GTF2A2 | 14383296 | PREDICTED: Canis lupus familiaris general transcription factor IIA, 2, 12kDa (GTF2A2), transcript variant X3, mRNA. | transcription, DNA-templated // regulation of transcription, DNA-templated // transcription from RNA polymerase II promoter // transcription initiation from RNA polymerase II promoter // positive regulation of transcription from RNA polymerase II promoter // positive regulation of sequence-specific DNA binding transcription factor activity // RNA polymerase II transcriptional preinitiation complex assembly | Cluster0140 |
| PAPD4 | 14376885 | PREDICTED: Canis lupus familiaris PAP associated domain containing 4 (PAPD4), transcript variant X1, mRNA. | --- | Cluster0140 |
| TMEM106C | 14365873 | PREDICTED: Canis lupus familiaris transmembrane protein 106C (TMEM106C), transcript variant X2, mRNA. | --- | Cluster0140 |

**Table S2.** Functional analysis chart summary of clusters resulting from GCN analysis showing the top 10 GO terms for clusters associated with specific gene expression patterns (Table S1). Rows are ranked according to significance (lowest -value and FDR q-value). BP, biological process; CC, cellular component; MF, molecular function.

| | GOTERM | Term | Gene count |
|---|---|---|---|
| Down-regulated in CKCS | CC | Z disc | 17 |
| | BP | Sarcomere organization | 9 |
| | BP | Cardiac muscle contraction | 8 |
| | CC | Sarcolemma | 7 |
| | CC | Sarcoplasmic reticulum membrane | 5 |
| | CC | M band | 4 |
| | CC | Sarcomere | 4 |
| | CC | Actin cytoskeleton | 8 |
| | CC | I band | 4 |
| | CC | Myelin sheath | 8 |
| | | | |
| Down-regulated in all disease valves | CC | Extracellular matrix | 5 |
| | CC | Cell surface | 7 |
| | MF | Alpha-amylase activity | 2 |
| | MF | Cation binding | 2 |
| | MF | Heparin binding | 3 |
| | CC | Extracellular space | 7 |
| | CC | Integral component of plasma membrane | 6 |
| | BP | Atrial cardiac muscle cell action potential | 2 |
| | BP | Ventricular cardiac muscle cell action potential | 2 |
| | BP | Negative regulation of stress fiber assembly | 2 |

| | | | |
|---|---|---|---|
| Upregulated in all disease valves | BP | Inflammatory response | 12 |
| | CC | Extracellular exosome | 52 |
| | CC | Cytoplasmic dynein complex | 4 |
| | BP | Monocyte chemotaxis | 5 |
| | CC | Nuclear envelope | 4 |
| | BP | Cell-matrix adhesion | 5 |
| | CC | Smooth muscle contractile fiber | 2 |
| | CC | Box H/ACA snoRNP complex | 2 |
| | BP | Positive regulation of telomerase RNA localization to Cajal body | 3 |
| | CC | Endoplasmic reticulum | 13 |
