# Supplementary material for: Comparative transcriptomic profiling of myxomatous mitral valve disease in the cavalier King Charles spaniel
Source: BMC Vet Res. 2020 Sep 23;16:350. doi: 10.1186/s12917-020-02542-w (PMC7509937; doi:10.1186/s12917-020-02542-w)
Supplement: Supplementary file 2 — Additional file 2 Lists of differentially expressed genes (Table S3) and GO term enrichment analysis (Table S4) for comparisons between CKCS and normal valves. [file 12917_2020_2542_MOESM2_ESM.pdf]

Table S3. Gene list CKCS vs Normal (599 differentially expressed genes; 328 down, 271 up)

Fold Change	Gene Symbol	Description
-17.18	MB	myoglobin
-15.05	MYL4	myosin, light chain 4, alkali; atrial, embryonic
-14.26	CASQ2	calsequestrin 2 (cardiac muscle)
-12.94	ACTN2	actinin, alpha 2
-12.82	ACTA1	actin, alpha 1, skeletal muscle
-10.64	CKM	creatine kinase, muscle
-10.48	TTN	titin
-10.21	NRAP	nebulin-related anchoring protein
-10.1	MYH7	myosin, heavy chain 7, cardiac muscle, beta; myosin, heavy chain 6, cardiac muscle, alpha
-9.22	DSC2	desmocollin 2
-9.18	NKAIN2	Na+/K+ transporting ATPase interacting 2
-9.02	PGAM2	phosphoglycerate mutase 2 (muscle)
-7.65	NEBL	nebulette
-6.82	CILP	cartilage intermediate layer protein, nucleotide pyrophosphohydrolase
-6.78	CASQ2	calsequestrin 2 (cardiac muscle)
-6.04	TNMD	tenomodulin
-5.99	ALPK2	alpha-kinase 2
-5.36	COX6A2	cytochrome c oxidase subunit VIa polypeptide 2
-5.32	PALLD	palladin, cytoskeletal associated protein
-5.29	HHATL	hedgehog acyltransferase-like
-5.01	LAMA2	laminin, alpha 2
-5.01	C28H10orf71	chromosome 28 open reading frame, human C10orf71
-4.92	MEI4	meiotic double-stranded break formation protein 4
-4.91	GJB6	gap junction protein, beta 6, 30kDa
-4.84	WIF1	WNT inhibitory factor 1
-4.71	ATP1A3	ATPase, Na+/K+ transporting, alpha 3 polypeptide
-4.65	TNNI3	troponin I type 3 (cardiac)
-4.56	CMYA5	cardiomyopathy associated 5
-4.5	FSTL4	follistatin-like 4
-4.5	PPARGC1A	peroxisome proliferator-activated receptor gamma, coactivator 1 alpha
-4.33	LMO3	LIM domain only 3 (rhombotin-like 2)
-4.27	KLHL41	kelch-like family member 41
-4.25	LOC488818	fibroblast growth factor-binding protein 1

-4.23	ADRA1A	adrenoceptor alpha 1A
-4.09	SLITRK6	SLIT and NTRK-like family, member 6
-3.99	FREM1	FRAS1 related extracellular matrix 1
-3.92	PLA2G5	phospholipase A2, group V
-3.9	ADPRHL1	ADP-ribosylhydrolase like 1
-3.85	MYL3	myosin, light chain 3, alkali; ventricular, skeletal, slow
-3.84	MIR99A-1	microRNA mir-99a-1
-3.81	HIF3A	hypoxia inducible factor 3, alpha subunit
-3.74	FSTL4	follistatin-like 4
-3.55	SCN3B	sodium channel, voltage gated, type III beta subunit
-3.5	SCN4B	sodium channel, voltage gated, type IV beta subunit
-3.45	ADAMTS15	ADAM metallopeptidase with thrombospondin type 1 motif, 15
-3.41	LAMA2	laminin, alpha 2
-3.39	SYNPO2L	synaptopodin 2-like
-3.32	LRRC2	leucine rich repeat containing 2
-3.31	GRIN2A	glutamate receptor, ionotropic, N-methyl D-aspartate 2A
-3.27	FMO2	flavin containing monooxygenase 2 (non-functional)
-3.24	KCND2	potassium channel, voltage gated Shal related subfamily D, member 2
-3.22	NT5E	5-nucleotidase, ecto (CD73)
-3.22	CORIN	corin, serine peptidase
-3.2	LOC479934	lipid phosphate phosphatase-related protein type 5
-3.17	HAPLN1	hyaluronan and proteoglycan link protein 1
-3.16	MPZL2	myelin protein zero-like 2
-3.07	TXLNB	taxilin beta
-3.05	AMIGO2	adhesion molecule with Ig-like domain 2
-3.04	GFRA2	GDNF family receptor alpha 2
-3.03	ABCA6	ATP-binding cassette, sub-family A (ABC1), member 6
-2.99	DSP	desmoplakin
-2.99	ASB12	ankyrin repeat and SOCS box containing 12
-2.97	ADCY2	adenylate cyclase 2 (brain)
-2.96	POPDC3	popeye domain containing 3
-2.95	ADCY2	adenylate cyclase 2 (brain)
-2.93	TMEM132C	transmembrane protein 132C
-2.9	SLC2A12	solute carrier family 2 (facilitated glucose transporter), member 12
-2.87	ABCC9	ATP-binding cassette, sub-family C (CFTR/MRP), member 9
-2.85	SLC24A2	solute carrier family 24 (sodium/potassium/calcium exchanger), member 2
-2.85	TCAP	titin-cap

-2.83	MIR218-1	microRNA mir-218-1
-2.76	CA14	carbonic anhydrase XIV
-2.75	MYLK3	myosin light chain kinase 3
-2.73	PDZD2	PDZ domain containing 2
-2.72	TNXB	tenascin XB
-2.71	TRDN	triadin
-2.71	RGS6	regulator of G-protein signaling 6
-2.69	KERA	keratocan
-2.68	SLIT2	slit guidance ligand 2
-2.68	TMEFF2	transmembrane protein with EGF-like and two follistatin-like domains 2
-2.68	SDK1	sidekick cell adhesion molecule 1
-2.65	GPR37	G protein-coupled receptor 37 (endothelin receptor type B-like)
-2.64	NEBL	nebulette
-2.64	CILP2	cartilage intermediate layer protein 2
-2.63	PTP4A3	protein tyrosine phosphatase type IVA, member 3
-2.61	MYOC	myocilin, trabecular meshwork inducible glucocorticoid response
-2.6	F2RL2	coagulation factor II (thrombin) receptor-like 2
-2.6	RASGRF2	Ras protein-specific guanine nucleotide-releasing factor 2
-2.58	CDH22	cadherin 22, type 2
-2.57	KIAA1024L	KIAA1024-like ortholog
-2.57	KCNJ8	potassium channel, inwardly rectifying subfamily J, member 8
-2.57	SRL	sarcalumenin
-2.56	COLCA2	colorectal cancer associated 2
-2.52	WIPF3	WAS/WASL interacting protein family, member 3
-2.51	SLC22A23	solute carrier family 22, member 23
-2.51	LIFR	leukemia inhibitory factor receptor alpha
-2.5	ACKR1	atypical chemokine receptor 1 (Duffy blood group)
-2.5	NEGR1	neuronal growth regulator 1
-2.48	CDC42EP2	CDC42 effector protein (Rho GTPase binding) 2
-2.46	KCND2	potassium channel, voltage gated Shal related subfamily D, member 2
-2.46	LMOD2	leiomodin 2 (cardiac)
-2.45	HCN1	hyperpolarization activated cyclic nucleotide gated potassium channel 1
-2.42	CNTFR	ciliary neurotrophic factor receptor
-2.42	KDR	kinase insert domain receptor
-2.4	PDZD2	PDZ domain containing 2
-2.39	PYGM	phosphorylase, glycogen, muscle

-2.38	RYR2	ryanodine receptor 2 (cardiac)
-2.37	RGS7BP	regulator of G-protein signaling 7 binding protein
-2.36	LAMA1	laminin, alpha 1
-2.35	RNF128	ring finger protein 128, E3 ubiquitin protein ligase
-2.33	CAV3	caveolin 3
-2.31	PER2	period circadian clock 2
-2.31	PCSK6	proprotein convertase subtilisin/kexin type 6
-2.31	NPR3	natriuretic peptide receptor 3
-2.31	FAM20A	family with sequence similarity 20, member A
-2.3	DRP2	dystrophin related protein 2
-2.28	PI15	peptidase inhibitor 15
-2.28	S100B	S100 calcium binding protein B
-2.27	SLC1A3	solute carrier family 1 (glial high affinity glutamate transporter), member 3
-2.24	KCNQ5	potassium channel, voltage gated KQT-like subfamily Q, member 5
-2.24	ENPEP	glutamyl aminopeptidase (aminopeptidase A)
-2.22	LOC478001	phytanoyl-CoA hydroxylase-like
-2.22	ANGPTL5	angiopoietin-like 5
-2.21	GJB2	gap junction protein, beta 2, 26kDa
-2.21	ADRB1	adrenoceptor beta 1
-2.21	FAM13A	family with sequence similarity 13, member A
-2.21	TMEFF2	transmembrane protein with EGF-like and two follistatin-like domains 2
-2.2	APOBEC2	apolipoprotein B mRNA editing enzyme, catalytic polypeptide-like 2
-2.2	CACNA1H	calcium channel, voltage-dependent, T type, alpha 1H subunit
-2.19	SLC37A1	solute carrier family 37 (glucose-6-phosphate transporter), member 1
-2.19	MASP1	mannan-binding lectin serine peptidase 1 (C4/C2 activating component of Ra-reactive factor)
-2.17	TTYH1	tweety family member 1
-2.17	IGSF3	immunoglobulin superfamily, member 3
-2.17	NID1	nidogen 1
-2.16	RASIP1	Ras interacting protein 1
-2.15	SGCG	sarcoglycan, gamma (35kDa dystrophin-associated glycoprotein)
-2.15	MN1	meningioma (disrupted in balanced translocation) 1
-2.15	SDK1	sidekick cell adhesion molecule 1
-2.14	TRPM3	transient receptor potential cation channel, subfamily M, member 3
-2.14	HRC	histidine rich calcium binding protein
-2.14	DOK6	docking protein 6
-2.14	SERINC2	serine incorporator 2

-2.14	GPLD1	glycosylphosphatidylinositol specific phospholipase D1
-2.12	CHRM2	cholinergic receptor, muscarinic 2
-2.11	TOX	thymocyte selection-associated high mobility group box
-2.1	LAPTM4B	lysosomal protein transmembrane 4 beta
-2.1	ADGRL3	adhesion G protein-coupled receptor L3
-2.1	ADCK3	aarF domain containing kinase 3
-2.09	KCNJ2	potassium channel, inwardly rectifying subfamily J, member 2
-2.08	GNAO1	guanine nucleotide binding protein (G protein), alpha activating activity polypeptide O
-2.07	KANK3	KN motif and ankyrin repeat domains 3
-2.07	CYGB	cytoglobin
-2.06	SLC4A4	solute carrier family 4 (sodium bicarbonate cotransporter), member 4
-2.06	FAM171A1	family with sequence similarity 171, member A1
-2.06	DECR1	2,4-dienoyl CoA reductase 1, mitochondrial
-2.06	AFF2	AF4/FMR2 family, member 2
-2.05	SEMA3D	sema domain, immunoglobulin domain (Ig), short basic domain, secreted, (semaphorin) 3D
-2.05	SLIT2	slit guidance ligand 2
-2.04	NTN1	netrin 1
-2.03	FRMD3	FERM domain containing 3
-2.03	SLC10A6	solute carrier family 10 (sodium/bile acid cotransporter), member 6
-2.02	FAM159A	family with sequence similarity 159, member A
-2.02	SCN2B	sodium channel, voltage gated, type II beta subunit
-2.01	EDNRA	endothelin receptor type A
-2	PCLO	piccolo presynaptic cytomatrix protein
-2	KCNJ5	potassium channel, inwardly rectifying subfamily J, member 5
-1.99	ADCYAP1R1	adenylate cyclase activating polypeptide 1 (pituitary) receptor type I
-1.99	NID1	nidogen 1
-1.99	AMIGO1	adhesion molecule with Ig-like domain 1
-1.98	CCK	cholecystokinin
-1.98	FAM81A	family with sequence similarity 81, member A
-1.97	SNTB1	syntrophin, beta 1 (dystrophin-associated protein A1, 59kDa, basic component 1)
-1.97	VWDE	von Willebrand factor D and EGF domains
-1.97	WFIKKN2	WAP, follistatin/kazal, immunoglobulin, kunitz and netrin domain containing 2
-1.94	FAM19A4	family with sequence similarity 19 (chemokine (C-C motif)-like), member A4
-1.94	GRIA3	glutamate receptor, ionotropic, AMPA 3

-1.93	MPP6	membrane protein, palmitoylated 6 (MAGUK p55 subfamily member 6)
-1.93	SORL1	sortilin-related receptor, L(DLR class) A repeats containing
-1.92	RBPMS2	RNA binding protein with multiple splicing 2
-1.92	PTGFR	prostaglandin F receptor (FP)
-1.91	ANGPTL4	angiopoietin-like 4
-1.91	ARGLU1	arginine and glutamate rich 1
-1.91	TNFRSF19	tumor necrosis factor receptor superfamily, member 19
-1.91	FAM53B	family with sequence similarity 53, member B
-1.91	SLCO5A1	solute carrier organic anion transporter family, member 5A1
-1.91	LIX1	limb and CNS expressed 1
-1.9	ATP2A2	ATPase, Ca++ transporting, cardiac muscle, slow twitch 2
-1.9	NGFR	nerve growth factor receptor
-1.89	CRISPLD2	cysteine-rich secretory protein LCCL domain containing 2
-1.89	MAP2K6	mitogen-activated protein kinase kinase 6
-1.89	ABCA9	ATP-binding cassette, sub-family A (ABC1), member 9
-1.88	KIAA1755	KIAA1755 ortholog
-1.87	KIAA1671	KIAA1671 ortholog
-1.87	PROX1	prospero homeobox 1
-1.86	ACKR2	atypical chemokine receptor 2
-1.86	C37H2orf88	chromosome 37 open reading frame, human C2orf88
-1.86	RNF207	ring finger protein 207
-1.85	ADCY2	adenylate cyclase 2 (brain)
-1.85	ARHGAP32	Rho GTPase activating protein 32
-1.85	FPGT	fucose-1-phosphate guanylyltransferase; TNNI3 interacting kinase
-1.84	ABHD10	abhydrolase domain containing 10
-1.84	TMEM52	transmembrane protein 52
-1.83	CACNA2D2	calcium channel, voltage-dependent, alpha 2/delta subunit 2
-1.82	SNTB1	syntrophin, beta 1 (dystrophin-associated protein A1, 59kDa, basic component 1)
-1.82	C1QTNF4	C1q and tumor necrosis factor related protein 4
-1.82	TMEM63C	transmembrane protein 63C
-1.8	CACHD1	cache domain containing 1
-1.78	RFX2	regulatory factor X, 2 (influences HLA class II expression)
-1.78	GATA2	GATA binding protein 2
-1.77	KCNQ5	potassium channel, voltage gated KQT-like subfamily Q, member 5
-1.77	ENPP2	ectonucleotide pyrophosphatase/phosphodiesterase 2
-1.77	CEP126	centrosomal protein 126kDa

-1.77	WNT9B	wingless-type MMTV integration site family, member 9B
-1.76	SOX10	SRY (sex determining region Y)-box 10
-1.76	THSD7A	thrombospondin, type I, domain containing 7A
-1.76	SCARA5	scavenger receptor class A, member 5
-1.76	VAMP2	vesicle-associated membrane protein 2 (synaptobrevin 2)
-1.75	DYSF	dysferlin
-1.75	PPP1R16B	protein phosphatase 1, regulatory subunit 16B
-1.75	ADAMTS8	ADAM metallopeptidase with thrombospondin type 1 motif, 8
-1.75	PPAP2B	phosphatidic acid phosphatase type 2B
-1.74	ADHFE1	alcohol dehydrogenase, iron containing, 1
-1.74	CABYR	calcium binding tyrosine-(Y)-phosphorylation regulated
-1.73	SEMA6C	sema domain, transmembrane domain (TM), and cytoplasmic domain, (semaphorin) 6C
-1.73	SYT17	synaptotagmin XVII
-1.73	TANC2	tetratricopeptide repeat, ankyrin repeat and coiled-coil containing 2
-1.72	COL6A3	collagen, type VI, alpha 3
-1.72	CCDC65	coiled-coil domain containing 65
-1.72	LIPC	lipase, hepatic
-1.72	ST8SIA5	ST8 alpha-N-acetyl-neuraminide alpha-2,8-sialyltransferase 5
-1.71	NFATC1	nuclear factor of activated T-cells, cytoplasmic, calcineurin-dependent 1
-1.71	ANK1	ankyrin 1, erythrocytic
-1.71	WDR54	WD repeat domain 54
-1.71	ATP9A	ATPase, class II, type 9A
-1.71	DDX31	DEAD (Asp-Glu-Ala-Asp) box polypeptide 31
-1.7	SHANK3	SH3 and multiple ankyrin repeat domains 3
-1.7	RCAN2	regulator of calcineurin 2
-1.7	NRSN2	neurensin 2
-1.7	UBE2QL1	ubiquitin-conjugating enzyme E2Q family-like 1
-1.7	ZCCHC24	zinc finger, CCHC domain containing 24
-1.7	BAIAP2	BAI1-associated protein 2
-1.7	LAMC3	laminin, gamma 3
-1.69	OGN	osteoglycin
-1.69	DLG2	discs, large homolog 2 (Drosophila)
-1.69	GCNT4	glucosaminyl (N-acetyl) transferase 4, core 2
-1.69	FHOD3	formin homology 2 domain containing 3
-1.69	PTGDS	prostaglandin D2 synthase 21kDa (brain)
-1.69	ABL1	ABL proto-oncogene 1, non-receptor tyrosine kinase

-1.68	ESR1	estrogen receptor 1
-1.68	B4GAT1	beta-1,4-glucuronyltransferase 1
-1.68	MAML3	mastermind-like transcriptional coactivator 3
-1.68	CKMT2	creatine kinase, mitochondrial 2 (sarcomeric)
-1.67	LGI3	leucine-rich repeat LGI family, member 3
-1.67	FAM184B	family with sequence similarity 184, member B
-1.66	MDGA1	MAM domain containing glycosylphosphatidylinositol anchor 1
-1.66	TNIK	TRAF2 and NCK interacting kinase
-1.66	ABI3	ABI family, member 3
-1.65	TMOD1	tropomodulin 1
-1.65	TBX1	T-box 1
-1.65	CCND2	cyclin D2
-1.65	SETBP1	SET binding protein 1
-1.64	CACNA2D1	calcium channel, voltage-dependent, alpha 2/delta subunit 1
-1.64	MYH7B	myosin, heavy chain 7B, cardiac muscle, beta
-1.64	SEMA4B	sema domain, immunoglobulin domain (Ig), transmembrane domain (TM) and short cytoplasmic domain, (semaphorin) 4B
-1.64	NTN1	netrin 1
-1.64	WNK4	WNK lysine deficient protein kinase 4; vacuolar protein sorting 25 homolog (S. cerevisiae)
-1.63	ELOVL6	ELOVL fatty acid elongase 6
-1.62	MOB3B	MOB kinase activator 3B
-1.62	IL17RD	interleukin 17 receptor D
-1.62	ISM1	isthmin 1, angiogenesis inhibitor
-1.62	C5H11orf63	chromosome 5 open reading frame, human C11orf63
-1.62	TANC2	tetratricopeptide repeat, ankyrin repeat and coiled-coil containing 2
-1.61	NAPEPLD	N-acyl phosphatidylethanolamine phospholipase D
-1.61	MICAL3	microtubule associated monooxygenase, calponin and LIM domain containing 3
-1.61	CYP27A1	cytochrome P450, family 27, subfamily A, polypeptide 1
-1.61	MOK	MOK protein kinase
-1.61	LOC479922	pancreatic alpha-amylase; pancreatic alpha-amylase-like
-1.6	OLFML1	olfactomedin-like 1
-1.6	TTC21A	tetratricopeptide repeat domain 21A
-1.6	APOLD1	apolipoprotein L domain containing 1
-1.6	STARD9	StAR-related lipid transfer (START) domain containing 9
-1.6	ALDH5A1	aldehyde dehydrogenase 5 family, member A1
-1.6	NFIA	nuclear factor I/A
-1.6	TRIM4	tripartite motif containing 4

-1.6	DSG2	desmoglein 2
-1.59	ITGA2	integrin, alpha 2 (CD49B, alpha 2 subunit of VLA-2 receptor)
-1.58	KIAA0355	KIAA0355 ortholog
-1.58	ADAMTSL4	ADAMTS-like 4
-1.58	SIK3	SIK family kinase 3
-1.57	IDNK	idnK, gluconokinase homolog (E. coli)
-1.57	CFAP54	cilia and flagella associated 54
-1.57	INSR	insulin receptor
-1.57	PLSCR4	phospholipid scramblase 4
-1.57	CCDC92	coiled-coil domain containing 92
-1.57	C32H4orf36	chromosome 32 open reading frame, human C4orf36
-1.57	PER3	period circadian clock 3
-1.57	LOC607276	pancreatic alpha-amylase-like; pancreatic alpha-amylase
-1.56	HACD4	3-hydroxyacyl-CoA dehydratase 4
-1.56	PDGFRL	platelet-derived growth factor receptor-like
-1.56	LOC102152842	zinc finger protein 512-like
-1.56	SEL1L3	sel-1 suppressor of lin-12-like 3 (C. elegans)
-1.56	CACNA1G	calcium channel, voltage-dependent, T type, alpha 1G subunit
-1.56	KSR1	kinase suppressor of ras 1
-1.55	ECM1	extracellular matrix protein 1
-1.55	MAML3	mastermind-like transcriptional coactivator 3
-1.55	PDE3B	phosphodiesterase 3B, cGMP-inhibited
-1.55	DCLK1	doublecortin-like kinase 1
-1.55	MYLK	myosin light chain kinase
-1.55	PPP1R12B	protein phosphatase 1, regulatory subunit 12B
-1.55	SYNE3	spectrin repeat containing, nuclear envelope family member 3
-1.55	PHKA2	phosphorylase kinase, alpha 2 (liver)
-1.54	SALL3	spalt-like transcription factor 3
-1.54	FAM189A2	family with sequence similarity 189, member A2
-1.54	ADGRD1	adhesion G protein-coupled receptor D1
-1.53	SLC25A42	solute carrier family 25, member 42
-1.53	SLC8B1	solute carrier family 8 (sodium/lithium/calcium exchanger), member B1
-1.53	PIK3C2B	phosphatidylinositol-4-phosphate 3-kinase, catalytic subunit type 2 beta
-1.53	AURKB	aurora kinase B
-1.52	PDGFRA	platelet-derived growth factor receptor, alpha polypeptide
-1.52	LOC480788	T-cell receptor beta-1 chain C region

-1.52	C4H10orf54	chromosome 4 open reading frame, human C10orf54
-1.51	ZBTB45	zinc finger and BTB domain containing 45
-1.51	CLIC5	chloride intracellular channel 5
-1.51	SLC7A2	solute carrier family 7 (cationic amino acid transporter, y+ system), member 2
-1.51	TLE2	transducin-like enhancer of split 2
-1.51	FZD4	frizzled class receptor 4
-1.51	ZSWIM1	zinc finger, SWIM-type containing 1
-1.51	KCNA1	potassium channel, voltage gated shaker related subfamily A, member 1
-1.51	NCAM1	neural cell adhesion molecule 1
1.51	COX7A1	cytochrome c oxidase subunit VIIa polypeptide 1 (muscle)
1.51	SHMT2	serine hydroxymethyltransferase 2 (mitochondrial)
1.51	CORO2A	coronin, actin binding protein, 2A
1.51	RBM28	RNA binding motif protein 28
1.51	PRDX1	peroxiredoxin 1
1.51	ATP6V0B	ATPase, H+ transporting, lysosomal 21kDa, V0 subunit b
1.51	DUSP6	dual specificity phosphatase 6
1.51	PPP1R14B	protein phosphatase 1, regulatory (inhibitor) subunit 14B
1.51	NUDT22	nudix (nucleoside diphosphate linked moiety X)-type motif 22; DnaJ (Hsp40) homolog, subfamily C, member 4
1.51	CCDC159	coiled-coil domain containing 159
1.51	SPC25	SPC25, NDC80 kinetochore complex component
1.51	PRR7	proline rich 7 (synaptic)
1.51	RAB31	RAB31, member RAS oncogene family
1.51	NLRP3	NLR family, pyrin domain containing 3
1.51	GSPT2	G1 to S phase transition 2
1.51	ACSL4	acyl-CoA synthetase long-chain family member 4
1.52	GNE	glucosamine (UDP-N-acetyl)-2-epimerase/N-acetylmannosamine kinase
1.52	PRDM5	PR domain containing 5
1.53	DRAP1	DR1-associated protein 1 (negative cofactor 2 alpha)
1.53	DSTN	destrin (actin depolymerizing factor)
1.53	PLCB1	phospholipase C, beta 1 (phosphoinositide-specific)
1.53	PLCB1	phospholipase C, beta 1 (phosphoinositide-specific)
1.53	CRIP1	cysteine-rich protein 1 (intestinal)
1.53	SLC46A1	solute carrier family 46 (folate transporter), member 1
1.54	ADAM22	ADAM metallopeptidase domain 22
1.54	CHEK1	checkpoint kinase 1

1.54	POLR3K	polymerase (RNA) III (DNA directed) polypeptide K, 12.3 kDa
1.54	GORAB	golgin, RAB6-interacting
1.54	SKA2	spindle and kinetochore associated complex subunit 2
1.55	SUCLG1	succinate-CoA ligase, alpha subunit
1.55	ARMC6	armadillo repeat containing 6
1.55	LOC480571	septin-4
1.56	COMMD8	COMM domain containing 8
1.56	TMEM106C	transmembrane protein 106C
1.56	UBTD1	ubiquitin domain containing 1
1.56	ATOX1	antioxidant 1 copper chaperone
1.57	OSTM1	osteopetrosis associated transmembrane protein 1
1.57	AZIN2	antizyme inhibitor 2
1.57	MIR8824	microRNA mir-8824
1.57	GNPNAT1	glucosamine-phosphate N-acetyltransferase 1
1.57	SLC38A5	solute carrier family 38, member 5
1.58	CBWD2	COBW domain containing 2
1.58	ALDH1L2	aldehyde dehydrogenase 1 family, member L2
1.58	CASS4	Cas scaffolding protein family member 4
1.58	MED10	mediator complex subunit 10
1.58	PAM16	presequence translocase-associated motor 16 homolog (S. cerevisiae)
1.58	SSX2IP	synovial sarcoma, X breakpoint 2 interacting protein
1.58	CYR61	cysteine-rich, angiogenic inducer, 61
1.59	RNASET2	ribonuclease T2
1.59	ACYP2	acylphosphatase 2, muscle type
1.59	TAF9	TAF9 RNA polymerase II, TATA box binding protein (TBP)-associated factor, 32kDa; adenylate kinase 6
1.59	COL4A1	collagen, type IV, alpha 1
1.59	EAF1	ELL associated factor 1
1.6	CCND3	cyclin D3
1.6	NDUFB2	NADH dehydrogenase (ubiquinone) 1 beta subcomplex, 2, 8kDa
1.6	HSPA13	heat shock protein 70kDa family, member 13
1.6	H2AFZ	H2A histone family, member Z
1.6	LOC607207	myosin regulatory light polypeptide 9
1.61	GLIPR1	GLI pathogenesis-related 1
1.61	SLC31A2	solute carrier family 31 (copper transporter), member 2
1.61	PLXNC1	plexin C1
1.61	CALCA	calcitonin-related polypeptide alpha
1.61	ST8SIA4	ST8 alpha-N-acetyl-neuraminide alpha-2,8-sialyltransferase 4

1.61	BPNT1	3(2), 5-bisphosphate nucleotidase 1
1.61	PLA2G4A	phospholipase A2, group IVA (cytosolic, calcium-dependent)
1.62	CKAP4	cytoskeleton-associated protein 4
1.62	MGARP	mitochondria-localized glutamic acid-rich protein
1.62	MED21	mediator complex subunit 21
1.62	FST	follistatin
1.62	PARP8	poly (ADP-ribose) polymerase family, member 8
1.62	RBBP8	retinoblastoma binding protein 8
1.63	ZNF428	zinc finger protein 428
1.63	PDLIM4	PDZ and LIM domain 4
1.63	MAPK13	mitogen-activated protein kinase 13
1.63	MNF1	mitochondrial nucleoid factor 1
1.63	RND1	Rho family GTPase 1
1.63	SLC16A12	solute carrier family 16, member 12
1.63	TAGLN	transgelin
1.63	SPIRE1	spire-type actin nucleation factor 1
1.64	FKBP2	FK506 binding protein 2, 13kDa
1.64	HERC5	HECT and RLD domain containing E3 ubiquitin protein ligase 5
1.64	CD48	CD48 molecule
1.64	PTPRC	protein tyrosine phosphatase, receptor type, C
1.64	NME2	non-metastatic cells 2, protein (NM23B) expressed in; non-metastatic cells 1, protein (NM23A) expressed in
1.65	NANS	N-acetylneuraminic acid synthase
1.65	SUGCT	succinyl-CoA:glutarate-CoA transferase
1.65	UGGT2	UDP-glucose glycoprotein glucosyltransferase 2
1.65	TMEM256	transmembrane protein 256
1.65	RTCA	RNA 3-terminal phosphate cyclase
1.65	TCEAL4	transcription elongation factor A (SII)-like 4
1.66	FAM188A	family with sequence similarity 188, member A
1.66	DYNLRB1	dynein, light chain, roadblock-type 1
1.66	FAM174A	family with sequence similarity 174, member A
1.66	SATB2	SATB homeobox 2
1.66	AKAP5	A kinase (PRKA) anchor protein 5
1.67	GJB3	gap junction protein, beta 3, 31kDa
1.67	CA5B	carbonic anhydrase VB, mitochondrial
1.68	TMEM261	transmembrane protein 261
1.68	CLIC1	chloride intracellular channel 1
1.68	NGEF	neuronal guanine nucleotide exchange factor

1.68	ENOPH1	enolase-phosphatase 1
1.68	CD84	CD84 molecule
1.69	CDKN1A	cyclin-dependent kinase inhibitor 1A (p21, Cip1)
1.69	SEMA6B	sema domain, transmembrane domain (TM), and cytoplasmic domain, (semaphorin) 6B
1.69	MAPKAPK3	mitogen-activated protein kinase-activated protein kinase 3
1.69	CCDC115	coiled-coil domain containing 115
1.69	LAP3	leucine aminopeptidase 3
1.69	ALCAM	activated leukocyte cell adhesion molecule
1.7	NUAK1	NUAK family, SNF1-like kinase, 1
1.7	SOSTDC1	sclerostin domain containing 1
1.7	RAI14	retinoic acid induced 14
1.71	CFB	complement factor B; complement component 2
1.71	SYNC	syncoilin, intermediate filament protein
1.71	ELL2	elongation factor, RNA polymerase II, 2
1.72	PDE7B	phosphodiesterase 7B
1.72	KCNN4	potassium channel, calcium activated intermediate/small conductance subfamily N alpha, member 4
1.72	LOC102156311	uncharacterized LOC102156311
1.72	GNG11	guanine nucleotide binding protein (G protein), gamma 11
1.72	LBH	limb bud and heart development
1.73	C17H1orf162	chromosome 17 open reading frame, human C1orf162
1.73	RHNO1	RAD9-HUS1-RAD1 interacting nuclear orphan 1
1.73	PLAU	plasminogen activator, urokinase
1.73	KCNK1	potassium channel, two pore domain subfamily K, member 1
1.73	SLC45A1	solute carrier family 45, member 1
1.74	SEC11C	SEC11 homolog C, signal peptidase complex subunit
1.74	BNC2	basonuclin 2
1.74	EPHA2	EPH receptor A2
1.74	BLNK	B-cell linker
1.74	ANGPTL1	angiopoietin-like 1
1.75	LOC102154654	selenoprotein K-like
1.75	NCF2	neutrophil cytosolic factor 2
1.76	BANK1	B-cell scaffold protein with ankyrin repeats 1
1.76	LAMTOR2	late endosomal/lysosomal adaptor, MAPK and MTOR activator 2
1.77	NCAPG2	non-SMC condensin II complex, subunit G2
1.77	RNASEH2C	ribonuclease H2, subunit C
1.77	RGS18	regulator of G-protein signaling 18

1.78	COL4A2	collagen, type IV, alpha 2
1.78	C1RL	complement component 1, r subcomponent-like
1.79	LSM1	LSM1 homolog, mRNA degradation associated
1.79	PHPT1	phosphohistidine phosphatase 1
1.8	HDAC9	histone deacetylase 9
1.8	NKX3-1	NK3 homeobox 1
1.81	C24H20orf24	chromosome 24 open reading frame, human C20orf24
1.81	GAP43	growth associated protein 43
1.82	DZIP1	DAZ interacting zinc finger protein 1
1.82	PRC1	protein regulator of cytokinesis 1
1.82	ENO1	enolase 1, (alpha)
1.83	TYROBP	TYRO protein tyrosine kinase binding protein
1.83	LOXL3	lysyl oxidase-like 3
1.83	CENPF	centromere protein F, 350/400kDa
1.84	GREM1	gremlin 1, DAN family BMP antagonist
1.84	CD86	CD86 molecule
1.85	C17H1orf54	chromosome 17 open reading frame, human C1orf54
1.86	FBXO27	F-box protein 27
1.87	ATP8B1	ATPase, aminophospholipid transporter, class I, type 8B, member 1
1.87	ADAM22	ADAM metallopeptidase domain 22
1.87	PRR15	proline rich 15
1.87	NPAS3	neuronal PAS domain protein 3
1.88	OSBPL10	oxysterol binding protein-like 10
1.89	ETF1	eukaryotic translation termination factor 1
1.89	RNF19B	ring finger protein 19B
1.89	LOC100856200	histone H2A type 1
1.9	PDPN	podoplanin
1.9	SBSPON	somatomedin B and thrombospondin, type 1 domain containing
1.91	SEC61B	Sec61 translocon beta subunit
1.91	CKAP2L	cytoskeleton associated protein 2-like
1.91	ARNTL2	aryl hydrocarbon receptor nuclear translocator-like 2
1.91	BTK	Bruton agammaglobulinemia tyrosine kinase
1.93	LOC611446	leukocyte immunoglobulin-like receptor subfamily A member 6; leukocyte immunoglobulin-like receptor subfamily B member 4
1.93	CDKN2B	cyclin-dependent kinase inhibitor 2B (p15, inhibits CDK4)
1.93	MFSD2A	major facilitator superfamily domain containing 2A
1.93	CD70	CD70 molecule
1.94	VAV1	vav 1 guanine nucleotide exchange factor

1.95	SKAP2	src kinase associated phosphoprotein 2
1.96	FOXS1	forkhead box S1
1.97	NOV	nephroblastoma overexpressed
1.97	TBXAS1	thromboxane A synthase 1 (platelet)
1.97	USP18	ubiquitin specific peptidase 18
1.99	NME1	non-metastatic cells 1, protein (NM23A) expressed in
2	BNC2	basonuclin 2
2	LRRC25	leucine rich repeat containing 25
2	LTBP2	latent transforming growth factor beta binding protein 2
2.02	SPI1	Spi-1 proto-oncogene
2.02	MRVI1	murine retrovirus integration site 1 homolog
2.02	LYZF2	lysozyme C, milk isozyme-like
2.02	SLC5A3	solute carrier family 5 (sodium/myo-inositol cotransporter), member 3; sodium/myo-inositol cotransporter
2.04	UCHL1	ubiquitin carboxyl-terminal esterase L1 (ubiquitin thiolesterase)
2.05	PON3	paraoxonase 3
2.05	SYTL2	synaptotagmin-like 2
2.05	BMP6	bone morphogenetic protein 6
2.06	CH25H	cholesterol 25-hydroxylase
2.07	VCAM1	vascular cell adhesion molecule 1
2.09	DDC	dopa decarboxylase (aromatic L-amino acid decarboxylase)
2.11	BLVRB	biliverdin reductase B
2.11	SYNDIG1	synapse differentiation inducing 1
2.12	STK17B	serine/threonine kinase 17b
2.14	LOC100856638	uridine phosphorylase 1-like; uridine phosphorylase 1
2.15	IFITM10	interferon induced transmembrane protein 10
2.16	CLEC3A	C-type lectin domain family 3, member A
2.17	SERPINI1	serpin peptidase inhibitor, clade I (neuroserpin), member 1
2.17	CCL5	chemokine (C-C motif) ligand 5
2.18	ADAM28	ADAM metallopeptidase domain 28
2.2	ARAP2	ArfGAP with RhoGAP domain, ankyrin repeat and PH domain 2
2.21	TREM2	triggering receptor expressed on myeloid cells 2
2.21	CGREF1	cell growth regulator with EF-hand domain 1
2.21	TYSND1	trypsin domain containing 1
2.22	TNFRSF12A	tumor necrosis factor receptor superfamily, member 12A
2.24	ID3	inhibitor of DNA binding 3, dominant negative helix-loop-helix protein
2.25	HENMT1	HEN1 methyltransferase homolog 1 (Arabidopsis)

2.27	MT2A	metallothionein 1H
2.31	DAPP1	dual adaptor of phosphotyrosine and 3-phosphoinositides
2.32	GCSAM	germinal center-associated, signaling and motility
2.32	NLGN4X	neuroligin 4, X-linked
2.35	C10H2orf40	chromosome 10 open reading frame, human C2orf40
2.37	TNFRSF11B	tumor necrosis factor receptor superfamily, member 11b
2.38	NTRK3	neurotrophic tyrosine kinase, receptor, type 3
2.38	CXHXorf21	chromosome X open reading frame, human CXorf21
2.39	LOC487977	cell surface glycoprotein CD200 receptor 1
2.39	RGS2	regulator of G-protein signaling 2
2.39	HAVCR1	hepatitis A virus cellular receptor 1
2.4	EVI2B	ecotropic viral integration site 2B
2.5	ABCC4	ATP-binding cassette, sub-family C (CFTR/MRP), member 4
2.51	LOC100856577	1,2-dihydroxy-3-keto-5-methylthiopentene dioxygenase
2.52	C3AR1	complement component 3a receptor 1
2.53	EPHA3	EPH receptor A3
2.57	RXFP1	relaxin/insulin-like family peptide receptor 1
2.58	KCNMB1	potassium channel subfamily M regulatory beta subunit 1
2.59	HOXD8	homeobox D8
2.62	PTGS2	prostaglandin-endoperoxide synthase 2 (prostaglandin G/H synthase and cyclooxygenase)
2.69	HTR2B	5-hydroxytryptamine (serotonin) receptor 2B, G protein-coupled
2.7	CCL8	chemokine (C-C motif) ligand 8
2.74	HTR3B	5-hydroxytryptamine (serotonin) receptor 3B, ionotropic
2.75	CNN1	calponin 1, basic, smooth muscle
2.77	PAPPA2	pappalysin 2
2.79	FCGR1A	Fc fragment of IgG, high affinity Ia, receptor (CD64)
2.81	IL18	interleukin 18
2.87	KCNJ15	potassium channel, inwardly rectifying subfamily J, member 15
2.9	LOC612564	membrane-spanning 4-domains subfamily A member 7
2.97	LRRC3B	leucine rich repeat containing 3B
2.99	ANGPT1	angiopoietin 1
3.02	PLCXD3	phosphatidylinositol-specific phospholipase C, X domain containing 3
3.07	CLEC5A	C-type lectin domain family 5, member A
3.28	TPM2	tropomyosin 2 (beta)
3.34	TUBB3	tubulin, beta 3 class III
3.38	KCNK2	potassium channel, two pore domain subfamily K, member 2

3.43	CASP14	caspase 14, apoptosis-related cysteine peptidase
3.48	SLCO2A1	solute carrier organic anion transporter family, member 2A1
3.52	LOC611538	C-type lectin domain family 4 member E
3.58	MYH11	myosin, heavy chain 11, smooth muscle
4.03	IL1RL1	interleukin 1 receptor-like 1
4.06	ACTA2	actin, alpha 2, smooth muscle, aorta
4.18	IGFBP2	insulin-like growth factor binding protein 2, 36kDa
4.28	CRLF1	cytokine receptor-like factor 1
4.41	COL6A5	collagen, type VI, alpha 5
4.51	TNFSF15	tumor necrosis factor (ligand) superfamily, member 15
4.6	CCL7	chemokine (C-C motif) ligand 7
4.63	HSP70	heat shock protein 70
4.75	FGG	fibrinogen gamma chain
4.75	RGS4	regulator of G-protein signaling 4
4.92	COL6A5	collagen type VI alpha 5 chain
4.92	COL6A5	collagen type VI alpha 5 chain
4.92	COL6A5	collagen type VI alpha 5 chain
5.07	SERPINE1	serpin peptidase inhibitor, clade E (nexin, plasminogen activator inhibitor type 1), member 1
5.4	CLEC7A	C-type lectin domain family 7, member A
5.44	HSP70	heat shock protein 70
5.74	SFRP2	secreted frizzled-related protein 2
6.14	CDKN2A	cyclin-dependent kinase inhibitor 2A (melanoma, p16, inhibits CDK4)
6.24	CCL13	chemokine (C-C motif) ligand 13
7.46	LRRN1	leucine rich repeat neuronal 1
8.01	CDKN2A	cyclin-dependent kinase inhibitor 2A (melanoma, p16, inhibits CDK4)

Table S4. Functional analysis chart summary of CKCS samples compared to normal valves showing the top 10 GO terms. Rows are ranked according to significance (lowest p-value and FDR q-value). BP, biological process; CC, cellular component; MF, molecular function.

	GOTERM	Term	Gene count
Up-regulated	BP	Cell-cell signalling	7
	BP	Immune response	10
	BP	Inflammatory response	10
	CC	Extracellular space	25
	BP	Positive regulation of inflammatory response	5
	BP	Positive regulation of ERK1 and ERK2 cascade	8
	MF	CCR chemokine receptor binding	4
	BP	Lymphocyte chemotaxis	4
	BP	Neutrophil chemotaxis	5
	BP	Eosinophil chemotaxis	5
Down-Regulated	BP	Cardiac muscle contraction	8
	BP	Regulation of heart rate by cardiac conduction	8
	CC	Z disc	9
	BP	Sarcomere organisation	6
	CC	Voltage-gated calcium channel complex	6
	MF	Calcium ion binding	24
	BP	Regulation of ventricular cardiac muscle action potential	4
	BP	Regulation of force of heart contraction	4
	CC	Proteinaceous extracellular matrix	11
	CC	Integral component of plasma membrane	26
