# Supplementary material for: Comparative transcriptomic profiling of myxomatous mitral valve disease in the cavalier King Charles spaniel
Source: BMC Vet Res. 2020 Sep 23;16:350. doi: 10.1186/s12917-020-02542-w (PMC7509937; doi:10.1186/s12917-020-02542-w)
Supplement: Supplementary file 3 — Additional file 3 Lists of differentially expressed genes (Table S5) and GO term enrichment analysis (Table S6) for comparisons between CKCS and other breed diseased valves. [file 12917_2020_2542_MOESM3_ESM.pdf]

Table S5. DEG list for CKCS vs non-CKCS (161 differentially expressed genes; 134 down, 27 up).

Fold Change	Gene Symbol	Description
-25.48	CASQ2	calsequestrin 2 (cardiac muscle)
-22.59	ACTN2	actinin, alpha 2
-19.33	TTN	titin
-17	NRAP	nebulin-related anchoring protein
-15.91	ACTA1	actin, alpha 1, skeletal muscle
-15.85	MYH7	myosin, heavy chain 7, cardiac muscle, beta; myosin, heavy chain 6, cardiac muscle, alpha
-12.51	PGAM2	phosphoglycerate mutase 2 (muscle)
-12.14	NEBL	nebulette
-11.74	ALPK2	alpha-kinase 2
-11.38	CASQ2	calsequestrin 2 (cardiac muscle)
-10.64	HHATL	hedgehog acyltransferase-like
-8.97	C28H10orf71	chromosome 28 open reading frame, human C10orf71
-8.96	LMO3	LIM domain only 3 (rhombotin-like 2)
-8.75	PALLD	palladin, cytoskeletal associated protein
-8.74	TNNI3	troponin I type 3 (cardiac)
-8.62	DSC2	desmocollin 2
-8.57	ATP1A3	ATPase, Na+/K+ transporting, alpha 3 polypeptide
-7.97	CMYA5	cardiomyopathy associated 5
-6.86	COX6A2	cytochrome c oxidase subunit VIa polypeptide 2
-6.69	CORIN	corin, serine peptidase
-6.53	KLHL41	kelch-like family member 41
-6.22	ITGB6	integrin, beta 6
-5.99	PLA2G5	phospholipase A2, group V
-5.95	ADPRHL1	ADP-ribosylhydrolase like 1
-5.76	SYNPO2L	synaptopodin 2-like
-5.43	HPGDS	hematopoietic prostaglandin D synthase
-5.11	PPARGC1A	peroxisome proliferator-activated receptor gamma, coactivator 1 alpha
-5.05	TXLNB	taxilin beta
-5.05	TSHB	thyroid stimulating hormone, beta
-4.89	TCAP	titin-cap
-4.87	LAMA2	laminin, alpha 2
-4.82	MYLK3	myosin light chain kinase 3

-4.73	DSP	desmoplakin
-4.71	CA14	carbonic anhydrase XIV
-4.68	ASB12	ankyrin repeat and SOCS box containing 12
-4.62	LRRC2	leucine rich repeat containing 2
-4.51	SRL	sarcalumenin
-4.41	PYGM	phosphorylase, glycogen, muscle
-4.19	CACNA1H	calcium channel, voltage-dependent, T type, alpha 1H subunit
-4.1	TRDN	triadin
-4.02	ENSCAFG00000028066	Chromosome 1: 99,047,174-99,047,305 Novel snoRNA
-3.98	APOBEC2	apolipoprotein B mRNA editing enzyme, catalytic polypeptide-like 2
-3.96	PTP4A3	protein tyrosine phosphatase type IVA, member 3
-3.8	MLIP	muscular LMNA-interacting protein
-3.78	GNAO1	guanine nucleotide binding protein (G protein), alpha activating activity polypeptide O
-3.7	KCNE1	potassium channel, voltage gated subfamily E regulatory beta subunit 1
-3.7	RGS6	regulator of G-protein signaling 6
-3.53	LMOD2	leiomodin 2 (cardiac)
-3.53	CHRM2	cholinergic receptor, muscarinic 2
-3.49	NPR3	natriuretic peptide receptor 3
-3.44	ART3	ADP-ribosyltransferase 3
-3.29	LAMA2	laminin, alpha 2
-3.2	HSPB3	heat shock 27kDa protein 3
-3.13	FPGT	fucose-1-phosphate guanylyltransferase; TNNI3 interacting kinase
-3.08	LOC479934	lipid phosphate phosphatase-related protein type 5
-3.04	HRC	histidine rich calcium binding protein
-2.96	MYZAP	myocardial zonula adherens protein
-2.83	FABP3	fatty acid binding protein 3, muscle and heart
-2.76	SLC22A3	solute carrier family 22 (organic cation transporter), member 3
-2.74	CAV3	caveolin 3
-2.74	GPX3	glutathione peroxidase 3
-2.72	FREM1	FRAS1 related extracellular matrix 1
-2.72	RGS7BP	regulator of G-protein signaling 7 binding protein
-2.69	ADCK3	aarF domain containing kinase 3
-2.65	PCLO	piccolo presynaptic cytomatrix protein
-2.64	PLN	phospholamban

-2.64	HSPB7	heat shock 27kDa protein family, member 7 (cardiovascular)
-2.62	ENO3	enolase 3 (beta, muscle)
-2.58	XIRP2	xin actin binding repeat containing 2
-2.52	ADGRL3	adhesion G protein-coupled receptor L3
-2.52	KCNJ5	potassium channel, inwardly rectifying subfamily J, member 5
-2.51	ATP2A2	ATPase, Ca++ transporting, cardiac muscle, slow twitch 2
-2.44	DYSF	dysferlin
-2.43	KLHL31	kelch-like family member 31
-2.43	CXADR	coxsackie virus and adenovirus receptor
-2.4	CCDC85A	coiled-coil domain containing 85A
-2.4	ITGA7	integrin, alpha 7
-2.38	CPNE5	copine V
-2.33	FITM1	fat storage-inducing transmembrane protein 1
-2.32	KCNJ8	potassium channel, inwardly rectifying subfamily J, member 8
-2.31	DECR1	2,4-dienoyl CoA reductase 1, mitochondrial
-2.31	SLIT2	slit guidance ligand 2
-2.25	ADGRL3	adhesion G protein-coupled receptor L3
-2.23	SDK1	sidekick cell adhesion molecule 1
-2.17	RBPMS2	RNA binding protein with multiple splicing 2
-2.17	DES	desmin
-2.14	TNXB	tenascin XB
-2.14	ESRRG	estrogen-related receptor gamma
-2.13	DNAJC6	DnaJ (Hsp40) homolog, subfamily C, member 6
-2.08	TMEM132C	transmembrane protein 132C
-2.05	COBL	cordon-bleu WH2 repeat protein
-2.04	PER2	period circadian clock 2
-2.03	ADAMTS8	ADAM metallopeptidase with thrombospondin type 1 motif, 8
-2.01	FHOD3	formin homology 2 domain containing 3
-1.96	EDNRA	endothelin receptor type A
-1.96	SLC37A1	solute carrier family 37 (glucose-6-phosphate transporter), member 1
-1.96	NID1	nidogen 1
-1.93	SLC2A12	solute carrier family 2 (facilitated glucose transporter), member 12
-1.92	NGFR	nerve growth factor receptor

-1.91	HEYL	hes-related family bHLH transcription factor with YRPW motif-like
-1.89	LOC488818	fibroblast growth factor-binding protein 1
-1.86	PLTP	phospholipid transfer protein
-1.83	TOX	thymocyte selection-associated high mobility group box
-1.82	FAM13A	family with sequence similarity 13, member A
-1.82	PTGDS	prostaglandin D2 synthase 21kDa (brain)
-1.81	NID1	nidogen 1
-1.81	PPP1R12B	protein phosphatase 1, regulatory subunit 12B
-1.79	FITM2	fat storage-inducing transmembrane protein 2
-1.78	PPARA	peroxisome proliferator-activated receptor alpha
-1.76	RFX2	regulatory factor X, 2 (influences HLA class II expression)
-1.76	GOT1	glutamic-oxaloacetic transaminase 1, soluble
-1.75	BVES	blood vessel epicardial substance
-1.75	CASZ1	castor zinc finger 1
-1.74	MLLT11	myeloid/lymphoid or mixed-lineage leukemia; translocated to, 11
-1.72	RAB33A	RAB33A, member RAS oncogene family
-1.71	DRP2	dystrophin related protein 2
-1.7	NCAM1	neural cell adhesion molecule 1
-1.67	MCAM	melanoma cell adhesion molecule
-1.66	PROX1	prospero homeobox 1
-1.64	HACD4	3-hydroxyacyl-CoA dehydratase 4
-1.63	ATP9A	ATPase, class II, type 9A
-1.62	FAM160A1	family with sequence similarity 160, member A1
-1.62	CEP126	centrosomal protein 126kDa
-1.61	RCAN2	regulator of calcineurin 2
-1.6	FAM184B	family with sequence similarity 184, member B
-1.59	L1CAM	L1 cell adhesion molecule
-1.58	ACO2	aconitase 2, mitochondrial
-1.55	IQSEC1	IQ motif and Sec7 domain 1
-1.55	MDH2	malate dehydrogenase 2, NAD (mitochondrial)
-1.55	CACNA1G	calcium channel, voltage-dependent, T type, alpha 1G subunit
-1.54	TBC1D8	TBC1 domain family, member 8 (with GRAM domain)
-1.53	SEMA4D	sema domain, immunoglobulin domain (Ig), transmembrane domain (TM) and short cytoplasmic domain, (semaphorin) 4D

-1.53	SLC8B1	solute carrier family 8 (sodium/lithium/calcium exchanger), member B1
-1.51	CCDC92	coiled-coil domain containing 92
1.51	LOC106558240	formin-like protein 16; mitochondrial calcium uniporter regulator 1
1.51	MAT2B	methionine adenosyltransferase II, beta
1.52	ENOPH1	enolase-phosphatase 1
1.54	SWI5	SWI5 homologous recombination repair protein
1.56	CXHXorf21	chromosome X open reading frame, human CXorf21
1.57	C24H20orf24	chromosome 24 open reading frame, human C20orf24
1.57	TMEM106C	transmembrane protein 106C
1.59	DYNLRB1	dynein, light chain, roadblock-type 1
1.61	BLVRB	biliverdin reductase B
1.61	ETF1	eukaryotic translation termination factor 1
1.65	MASTL	microtubule associated serine/threonine kinase-like
1.67	SEC11C	SEC11 homolog C, signal peptidase complex subunit
1.67	NKX3-1	NK3 homeobox 1
1.68	SYNDIG1	synapse differentiation inducing 1
1.71	NOP10	NOP10 ribonucleoprotein
1.72	LOC102155956	putative uncharacterized protein ZNRD1-AS1
1.83	HENMT1	HEN1 methyltransferase homolog 1 (Arabidopsis)
1.84	EVI2B	ecotropic viral integration site 2B
1.86	ABCC4	ATP-binding cassette, sub-family C (CFTR/MRP), member 4
2.23	C3AR1	complement component 3a receptor 1
2.26	TMEM261	transmembrane protein 261
2.32	LOC612564	membrane-spanning 4-domains subfamily A member 7
2.46	LOC100856577	1,2-dihydroxy-3-keto-5-methylthiopentene dioxygenase
2.48	IL18	interleukin 18
2.52	RASL11A	RAS-like, family 11, member A
3.97	LOC476900	membrane-spanning 4-domains subfamily A member 4A
4.77	CLEC7A	C-type lectin domain family 7, member A

Table S6. Functional analysis chart summary of CKCS samples compared with non-CKCS samples showing the only GO term associated with up-regulated genes and the top 10 GO terms associated with down-regulated genes. Rows are ranked according to significance (lowest -value and FDR q-value). BP, biological process; CC, cellular component; MF, molecular function.

	GOTERM	Term	Gene count
Up-regulated	BP	L-methionine biosynthetic process from methylthioadenosine	2
Down-Regulated	CC	Z disc	9
	BP	Sarcomere organisation	6
	BP	Regulation of heart rate	5
	BP	Cardiac muscle contraction	5
	CC	Sarcoplasmic reticulum membrane	4
	BP	Skeletal muscle thin filament assembly	3
	CC	Voltage-gated calcium channel complex	4
	CC	Intercalated disc	4
	BP	Regulation of heart rate by cardiac conduction	4
	BP	Regulation of cardiac muscle contraction by regulation of the release of sequestered calcium ion	3
