# Supplementary material for: Comparative transcriptomic profiling of myxomatous mitral valve disease in the cavalier King Charles spaniel
Source: BMC Vet Res. 2020 Sep 23;16:350. doi: 10.1186/s12917-020-02542-w (PMC7509937; doi:10.1186/s12917-020-02542-w)
Supplement: Supplementary file 4 — Additional file 4 List of genes (Table S7) that were down-regulated in CKCS compared with both other breed diseased valves and normal valves and GO enrichment analysis (Table S8). [file 12917_2020_2542_MOESM4_ESM.pdf]

Table S7. List of differentially expressed genes that were downregulated in CKCS valves compared to both normal valves and diseased valves from other breeds (81 genes).

	**Gene symbol**	**Description**
Down-regulated	ACTA1	actin, alpha 1, skeletal muscle
	ACTN2	actinin, alpha 2
	ADAMTS8	ADAM metallopeptidase with thrombospondin type 1 motif, 8
	ADCK3	aarF domain containing kinase 3
	ADGRL3	adhesion G protein-coupled receptor L3
	ADPRHL1	ADP-ribosylhydrolase like 1
	ALPK2	alpha-kinase 2
	APOBEC2	apolipoprotein B mRNA editing enzyme, catalytic polypeptide-like 2
	ASB12	ankyrin repeat and SOCS box containing 12
	ATP1A3	ATPase, Na+/K+ transporting, alpha 3 polypeptide
	ATP2A2	ATPase, Ca++ transporting, cardiac muscle, slow twitch 2
	ATP9A	ATPase, class II, type 9A
	C28H10orf71	chromosome 28 open reading frame, human C10orf71
	CA14	carbonic anhydrase XIV
	CACNA1G	calcium channel, voltage-dependent, T type, alpha 1G subunit
	CASQ2	calsequestrin 2 (cardiac muscle)
	CCDC92	coiled-coil domain containing 92
	CMYA5	cardiomyopathy associated 5
	COBL	cordon-bleu WH2 repeat protein
	CORIN	corin, serine peptidase
	COX6A2	cytochrome c oxidase subunit VIa polypeptide 2
	DECR1	2,4-dienoyl CoA reductase 1, mitochondrial
	DRP2	dystrophin related protein 2
	DSC2	desmocollin 2
	DSP	desmoplakin
	DYSF	dysferlin
	EDNRA	endothelin receptor type A
	FAM13A	family with sequence similarity 13, member A
	FAM184B	family with sequence similarity 184, member B
	FHOD3	formin homology 2 domain containing 3
	FITM1	fat storage-inducing transmembrane protein 1
	FREM1	FRAS1 related extracellular matrix 1
	GNAO1	guanine nucleotide binding protein (G protein), alpha activating activity polypeptide O
	HACD4	3-hydroxyacyl-CoA dehydratase 4
	HHATL	hedgehog acyltransferase-like
	HRC	histidine rich calcium binding protein
	ITGB6	integrin, beta 6

	KCNJ5	potassium channel, inwardly rectifying subfamily J, member 5
	KCNJ8	potassium channel, inwardly rectifying subfamily J, member 8
	KLHL41	kelch-like family member 41
	LAMA2	laminin, alpha 2
	LMO3	LIM domain only 3 (rhombotin-like 2)
	LMOD2	leiomodin 2 (cardiac)
	LOC479934	lipid phosphate phosphatase-related protein type 5
	LOC488818	fibroblast growth factor-binding protein 1
	LRRC2	leucine rich repeat containing 2
	MYH7	myosin, heavy chain 7, cardiac muscle, beta; myosin, heavy chain 6, cardiac muscle, alpha
	MYLK3	myosin light chain kinase 3
	NCAM1	neural cell adhesion molecule 1
	NEBL	nebulette
	NGFR	nerve growth factor receptor
	NID1	nidogen 1
	NPR3	natriuretic peptide receptor 3
	NRAP	nebulin-related anchoring protein
	PALLD	palladin, cytoskeletal associated protein
	PCLO	piccolo presynaptic cytomatrix protein
	PER2	period circadian clock 2
	PGAM2	phosphoglycerate mutase 2 (muscle)
	PPARGC1A	peroxisome proliferator-activated receptor gamma, coactivator 1 alpha
	PPP1R12B	protein phosphatase 1, regulatory subunit 12B
	PROX1	prospero homeobox 1
	PTGDS	prostaglandin D2 synthase 21kDa (brain)
	PTP4A3	protein tyrosine phosphatase type IVA, member 3
	PYGM	phosphorylase, glycogen, muscle
	RBPMS2	RNA binding protein with multiple splicing 2
	RCAN2	regulator of calcineurin 2
	RFX2	regulatory factor X, 2 (influences HLA class II expression)
	RGS7BP	regulator of G-protein signaling 7 binding protein
	SDK1	sidekick cell adhesion molecule 1
	SLC2A12	solute carrier family 2 (facilitated glucose transporter), member 12
	SLC37A1	solute carrier family 37 (glucose-6-phosphate transporter), member 1
	SLC8B1	solute carrier family 8 (sodium/lithium/calcium exchanger), member B1
	SLIT2	slit guidance ligand 2
	SYNPO2L	synaptopodin 2-like
	TMEM132C	transmembrane protein 132C
	TNNI3	troponin I type 3 (cardiac)

	TNXB	tenascin XB
	TOX	thymocyte selection-associated high mobility group box
	TRDN	triadin
	TTN	titin
	TXLNB	taxilin beta
Up-regulated	ABCC4	ATP binding cassette subfamily C member 4
	BLVRB	biliverdin reductase B
	C24H20orf24	chromosome 24 open reading frame, human C20orf24
	C3AR1	complement C3a receptor 1
	CLEC7A	C-type lectin domain containing 7A
	CXHXorf21	chromosome X open reading frame, human CXorf21
	DYNLRB1	dynein light chain roadblock-type 1
	ENOPH1	enolase-phosphatase 1
	ETF1	eukaryotic translation termination factor 1
	EVI2B	ecotropic viral integration site 2B
	HENMT1	HEN methyltransferase 1
	IL18	interleukin 18
	LOC612564	unannotated gene
	LOC100856577	unannotated gene
	NKX3-1	NK3 homeobox 1
	SEC11C	SEC11 homolog C, signal peptidase complex subunit
	SYNDIG1	synapse differentiation inducing 1
	TMEM106C	transmembrane protein 106C
	TMEM261	transmembrane protein 261

Table S8. Functional analysis chart summary for differentially expressed genes that were down-regulated in CKCS valves compared to both normal valves and diseased valves from other breeds, showing the top 10 GO terms. Rows are ranked according to significance (lowest p-value and FDR q-value). BP, biological process; CC, cellular component.

	GOTERM	Term	Gene count
Up-regulated	BP	L-methionine biosynthetic process from methylthioadenosine	2
Down-Regulated	BP	Sarcomere organization	5
	CC	Z disc	6
	BP	Cardiac muscle contraction	4
	CC	Myofibril	3
	CC	Sarcoplasmic reticulum	3
	CC	Voltage gated calcium channel complex	3
	CC	Sarcoplasmic reticulum membrane	3
	CC	Stress fibre	3
	CC	Actin filament	3
	BP	Ventricular cardiac muscle tissue morphogenesis	3
