# Supplementary material for: Comparative transcriptomic profiling of myxomatous mitral valve disease in the cavalier King Charles spaniel
Source: BMC Vet Res. 2020 Sep 23;16:350. doi: 10.1186/s12917-020-02542-w (PMC7509937; doi:10.1186/s12917-020-02542-w)
Supplement: Supplementary file 5 — Additional file 5 Lists of differentially expressed genes (Table S9) and GO term enrichment analysis (Table S10) for comparisons between all diseased valves and normal valves. [file 12917_2020_2542_MOESM5_ESM.pdf]

Table S9. Differentially expressed gene list for comparison of all diseased valves and normal valves. Negative fold change indicates that gene expression was lower in diseased valves than normal valves. Only annotated genes are presented; there were a number of differentially expressed transcripts represented only by probeset ID. Some genes appear more than once because they have multiple probesets on the array. There were 50 down-regulated genes and 66 upregulated genes.

Fold Change	Gene Symbol	Description
-4.86	TNMD	tenomodulin
-4.65	NKAIN2	Na+/K+ transporting ATPase interacting 2
-4.44	CILP	cartilage intermediate layer protein, nucleotide pyrophosphohydrolase
-3.68	LOC102154136	protein MIS12 homolog
-3.46	LOC488818	fibroblast growth factor-binding protein 1
-3.27	NELL2	neural EGFL like 2
-3.05	NT5E	5-nucleotidase, ecto (CD73)
-2.94	GPR85	G protein-coupled receptor 85
-2.82	SCN3B	sodium channel, voltage gated, type III beta subunit
-2.81	TMEFF2	transmembrane protein with EGF-like and two follistatin-like domains 2
-2.77	FSTL4	follistatin-like 4
-2.62	ADAMTS15	ADAM metallopeptidase with thrombospondin type 1 motif, 15
-2.39	KCND2	potassium channel, voltage gated Shal related subfamily D, member 2
-2.37	TMEFF2	transmembrane protein with EGF-like and two follistatin-like domains 2
-2.36	SLC24A2	solute carrier family 24 (sodium/potassium/calcium exchanger), member 2
-2.23	CYP2B6	cytochrome P450 2B11
-2.1	KCNQ5	potassium channel, voltage gated KQT-like subfamily Q, member 5
-2.05	VWDE	von Willebrand factor D and EGF domains
-2.03		
-2.03	ALDH1A1	aldehyde dehydrogenase 1 family, member A1
-1.97	PNMT	phenylethanolamine N-methyltransferase
-1.9	SCIN	scinderin
-1.88	PTGFR	prostaglandin F receptor (FP)
-1.88	AFF2	AF4/FMR2 family, member 2
-1.86	CRISPLD2	cysteine-rich secretory protein LCCL domain containing 2
-1.81	TRPM3	transient receptor potential cation channel, subfamily M, member 3
-1.76	LGI2	leucine-rich repeat LGI family, member 2
-1.74	KCNQ5	potassium channel, voltage gated KQT-like subfamily Q, member 5

-1.72	PDZD2	PDZ domain containing 2
-1.68	ENOX1	ecto-NOX disulfide-thiol exchanger 1
-1.67	C1QTNF4	C1q and tumor necrosis factor related protein 4
-1.64	DLG2	discs, large homolog 2 (Drosophila)
-1.64	TRPC5	transient receptor potential cation channel, subfamily C, member 5
-1.63	SCARA5	scavenger receptor class A, member 5
-1.61	ENPP2	ectonucleotide pyrophosphatase/phosphodiesterase 2
-1.57	ITGA2	integrin, alpha 2 (CD49B, alpha 2 subunit of VLA-2 receptor)
-1.55	TANC2	tetratricopeptide repeat, ankyrin repeat and coiled-coil containing 2
-1.54	COL11A2	collagen, type XI, alpha 2
-1.54	GCNT4	glucosaminyl (N-acetyl) transferase 4, core 2
-1.51	OLFML1	olfactomedin-like 1
-1.51	PDE3B	phosphodiesterase 3B, cGMP-inhibited
1.51	MAPK13	mitogen-activated protein kinase 13
1.51	RAI14	retinoic acid induced 14
1.54	RNF19B	ring finger protein 19B
1.54	FAM174A	family with sequence similarity 174, member A
1.55	MAPKAPK3	mitogen-activated protein kinase-activated protein kinase 3
1.55	CCDC115	coiled-coil domain containing 115
1.56	MGARP	mitochondria-localized glutamic acid-rich protein
1.57	TAGLN	transgelin
1.59	SATB2	SATB homeobox 2
1.61	HDAC9	histone deacetylase 9
1.61	DZIP1	DAZ interacting zinc finger protein 1
1.61	LPP	LIM domain containing preferred translocation partner in lipoma
1.64	CDKN1A	cyclin-dependent kinase inhibitor 1A (p21, Cip1)
1.64	LBH	limb bud and heart development
1.67	SKAP2	src kinase associated phosphoprotein 2
1.67	CYR61	cysteine-rich, angiogenic inducer, 61
1.69	BNC2	basonuclin 2
1.7	ATP8B1	ATPase, aminophospholipid transporter, class I, type 8B, member 1
1.7	LOC100856200	histone H2A type 1
1.74	NOV	nephroblastoma overexpressed
1.77	BNC2	basonuclin 2
1.79	ADAM22	ADAM metallopeptidase domain 22
1.85	DAPP1	dual adaptor of phosphotyrosine and 3-phosphoinositides
1.86	GPER1	G protein-coupled estrogen receptor 1

1.88	GAP43	growth associated protein 43
1.94	BMP6	bone morphogenetic protein 6
1.96	CLEC3A	C-type lectin domain family 3, member A
2.06	TREM2	triggering receptor expressed on myeloid cells 2
2.06	MRVI1	murine retrovirus integration site 1 homolog
2.09	ARAP2	ArfGAP with RhoGAP domain, ankyrin repeat and PH domain 2
2.14	NTRK3	neurotrophic tyrosine kinase, receptor, type 3
2.18	KCNMB1	potassium channel subfamily M regulatory beta subunit 1
2.25	HOXD8	homeobox D8
2.38		
2.43	PAPPA2	pappalysin 2
2.48	PLCXD3	phosphatidylinositol-specific phospholipase C, X domain containing 3
2.5	ANGPT1	angiopoietin 1
2.65	HTR2B	5-hydroxytryptamine (serotonin) receptor 2B, G protein-coupled
2.86	LRRC3B	leucine rich repeat containing 3B
2.88	CNN1	calponin 1, basic, smooth muscle
3.04	EPHA3	EPH receptor A3
3.18	TPM2	tropomyosin 2 (beta)
3.57	CRLF1	cytokine receptor-like factor 1
3.86	SERPINE1	serpin peptidase inhibitor, clade E (nexin, plasminogen activator inhibitor type 1), member 1
3.91	MYH11	myosin, heavy chain 11, smooth muscle
4.09	ACTA2	actin, alpha 2, smooth muscle, aorta
4.37	CDKN2A	cyclin-dependent kinase inhibitor 2A (melanoma, p16, inhibits CDK4)
4.51	ACTG2	actin, gamma 2, smooth muscle, enteric
4.81	LRRN1	leucine rich repeat neuronal 1
5.19	CNTNAP4	contactin associated protein-like 4
5.79	CDKN2A	cyclin-dependent kinase inhibitor 2A (melanoma, p16, inhibits CDK4)
5.86	SFRP2	secreted frizzled-related protein 2

Table S10. Functional analysis chart summary for differentially expressed genes in all diseased valves compared to normal valves showing the top 10 GO terms. Rows are ranked according to significance (lowest p-value and FDR q-value). BP, biological process; CC, cellular component; MF, molecular function. Only four GO terms were found for the down-regulated genes.

	GOTERM	Term	Gene count
Up-regulated	MF	Integrin binding	3
	BP	Positive regulation of osteoblast differentiation	3
	BP	Negative regulation of cell death	3
	BP	Mesenchyme migration	2
	BP	Cell signalling	3
	BP	Positive regulation of ERK1 and ERK2 cascade	4
	BP	Negative regulation of cell growth	3
	BP	Positive regulation of cell proliferation	4
	CC	Filopodium	2
	BP	Angiogenesis	3
Down-Regulated	CC	Cell surface	4
	MF	Calcium ion binding	5
	MF	Scavenger receptor activity	2
	BP	Negative regulation of angiogenesis	2
