# Supplementary material for: Comparative transcriptomic profiling of myxomatous mitral valve disease in the cavalier King Charles spaniel
Source: BMC Vet Res. 2020 Sep 23;16:350. doi: 10.1186/s12917-020-02542-w (PMC7509937; doi:10.1186/s12917-020-02542-w)

**Figure S1.** Graphical representations of predicted down-stream regulator effects identified by IPA.

**A.** TGFβ1 in non-CKCS diseased valves compared to normal valves. TGFβ1 is shown to have its effect from the extracellular space on the genes in their cellular location. Genes are coloured red and green to represent up- or down-regulation in the dataset. Dotted lines connecting TGFβ1 to these genes show the expected effect of TGFB signalling: orange - activation, blue - inhibition, yellow -result inconsistent and grey – effect not predicted.


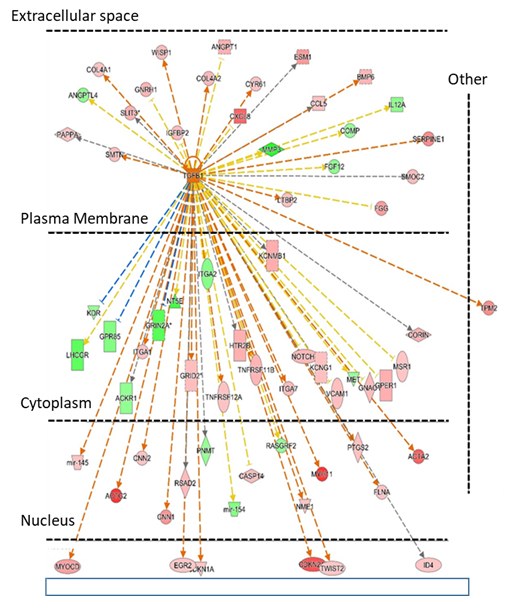


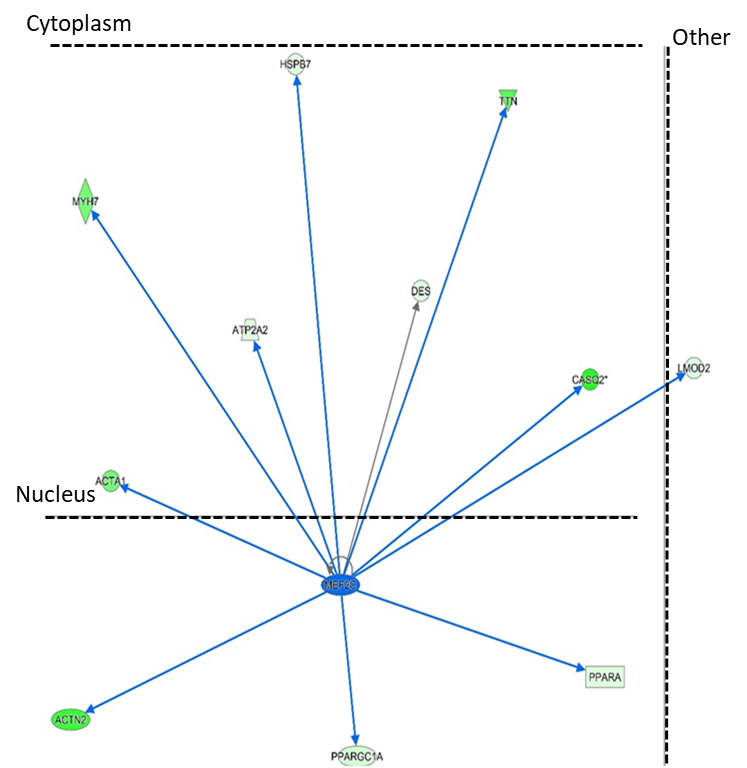
**B.** Myocyte-specific enhancer factor 2C (MEF2C) in CKCS compared to non-CKCS. MEF2C is shown to have its effect from the nucleus on the genes in the cytoplasm. Genes are coloured red and green to represent up- or down-regulation in the dataset. Dotted lines connecting MEF2C to these genes show the expected effect of MEF2C signalling: orange - activation, blue - inhibition, yellow -result inconsistent and grey – effect not predicted.

**C.** F2 (prothrombin) is shown to have its effect from the extracellular space on the genes in their cellular location. Genes are coloured red and green to represent up- or down-regulation in the dataset. Dotted lines connecting F2 to these genes show the expected effect of F2 signalling: orange - activation, blue - inhibition, yellow -result inconsistent and grey – effect not predicted.


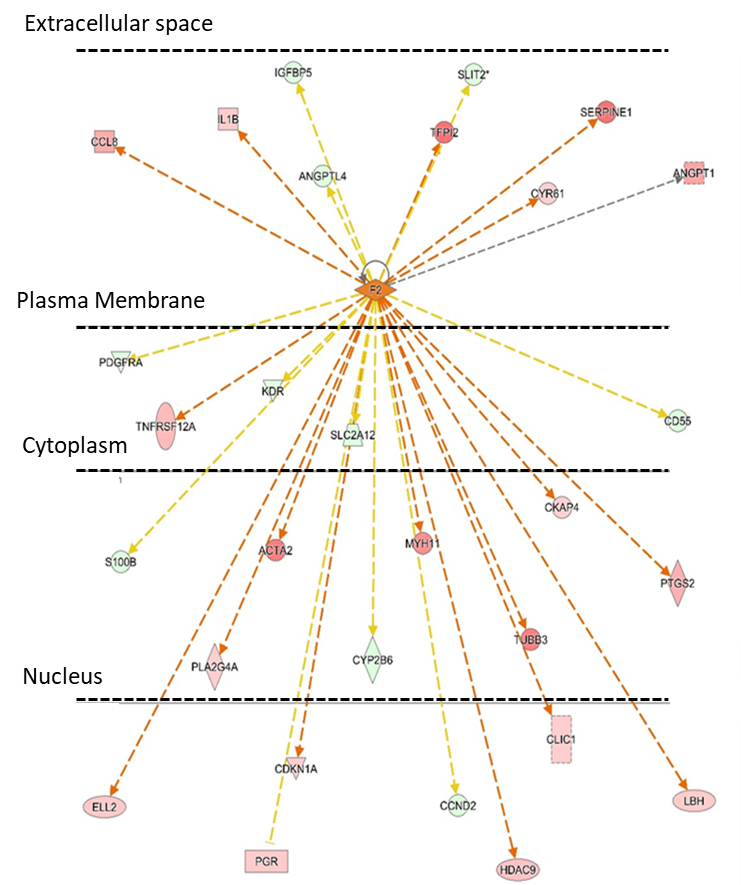

Supplement: Supplementary file 6 — Additional file 6 Graphical representations of predicted down-stream regulator effects identified by IPA (Figure S1). [file 12917_2020_2542_MOESM6_ESM.docx]
