# Supplementary material for: Comparative transcriptomic profiling of myxomatous mitral valve disease in the cavalier King Charles spaniel
Source: BMC Vet Res. 2020 Sep 23;16:350. doi: 10.1186/s12917-020-02542-w (PMC7509937; doi:10.1186/s12917-020-02542-w)

Table S11. Disease and function networks identified by IPA.

Top five disease and function networks associated with differentially expressed genes as identified by IPA in the comparisons of datasets.

**CKCS vs Normal**
1. Cell death and survival, cell cycle, cellular movement<br>2. Cardiovascular development and function, cell morphology, organ morphology<br>3. Cardiovascular disease, cell death and survival, connective tissue disorders<br>4. DNA replication, recombination and repair, cellular movement, cellular assembly and organisation<br>5. Connective tissue disorders, organismal injury and abnormalities, skeletal and muscular disorders
**CKCS vs non-CKCS**
1. Skeletal and muscle disorders, developmental disorder, hereditary disorder<br>2. Carbohydrate metabolism, energy production, small molecule biochemistry<br>3. Lipid metabolism, small molecule biochemistry, molecular transport<br>4. Cellular development, cellular growth and proliferation, organ development<br>5. Cellular function and maintenance, cardiovascular health, hereditary disorder
**All diseased vs Normal**
1. Cancer, organismal injury and abnormalities, reproductive system disease<br>2. Dermatological diseases and conditions<br>3. Gastrointestinal disease<br>4. Skeletal and Muscular System Development and Function<br>5. Endocrine System Disorders
**Non-CKCS vs Normal**
1. Organismal injury and abnormalities, renal and urologic, behaviour<br>2. Cancer, organismal injury and abnormalities, reproductive system disease<br>3. Cell morphology, cellular movement, hair and skin development and function<br>4. Skeletal and muscular system development, cardiovascular system development and function, organ development<br>5. Cell-to-cell signalling and interaction, tissue development, cardiovascular disease

Figure S2. Graphical representation of a disease and function network detected by IPA. Diagram shows the *Skeletal and Muscular Disorders and Developmental Disorder and Hereditary Disorder* network detected when comparing CKCS and non-CKCS. Genes are shown in their protein cellular location with red indicating up-regulation, green down-regulation and un-coloured showing no change in the dataset.

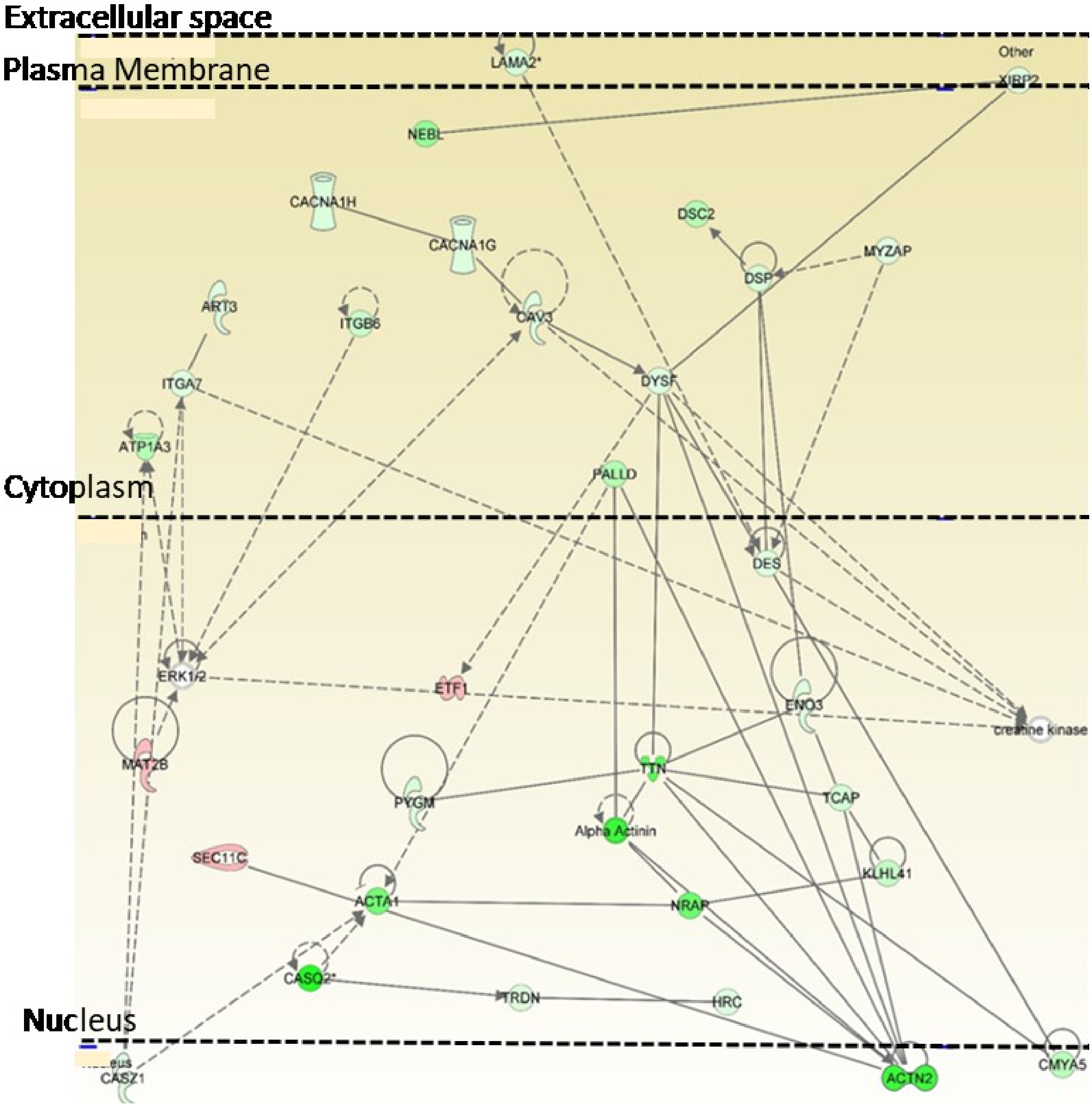

Supplement: Supplementary file 7 — Additional file 7 Disease and function networks identified by IPA (Table S11) and illustrative graphical representation (Figure S2). [file 12917_2020_2542_MOESM7_ESM.pdf]
